# Antibacterial activity of Au(I), Pt(II), and Ir(III) biotin conjugates prepared by the iClick reaction: Influence of the metal coordination sphere on the biological activity

**DOI:** 10.1007/s00775-024-02073-x

**Published:** 2024-08-29

**Authors:** Dominik Moreth, Lars Stevens-Cullinane, Thomas W. Rees, Victoria V.L. Müller, Adrien Pasquier, Ok-Ryul Song, Scott Warchal, Michael Howell, Jeannine Hess, Ulrich Schatzschneider

**Affiliations:** a Institut für Anorganische Chemie, Julius-Maximilians-Universität Würzburg, Am Hubland, D-97074 Würzburg, Germany; b Biological Inorganic Chemistry Laboratory, The Francis Crick Institute, London NW1 1AT, United Kingdom; c Department of Chemistry, King’s College London, Britannia House, 7 Trinity Street, London SE1 1DB, United Kingdom; d High Throughput Screening Science and Technology Platform, The Francis Crick Institute, London NW1 1AT, United Kingdom

## Abstract

A series of biotin-functionalized transition metal complexes was prepared by iClick reaction from the corresponding azido complexes with a novel alkyne-functionalized biotin derivative ([Au(triazolato^R,R’^)(PPh_3_)], [Pt(dpb)(triazolato^R,R’^)], [Pt(triazolato^R,R’^)(terpy)]PF_6_, and [Ir(ppy)(triazolato^R,R’^)(terpy)]PF_6_ with dpb = 1,3-di(2-pyridyl)benzene, ppy = 2-phenylpyridine, and terpy = 2,2’:6’,2’’-terpyridine and R = C_6_H_5_, R’ = biotin). The complexes were compared to reference compounds lacking the biotin moiety. The binding affinity towards avidin and streptavidin was evaluated with the HABA assay as well as isothermal titration calorimetry (ITC). All compounds exhibit the same binding stoichiometry of complex-to-avidin of 4:1, but the ITC results show that the octahedral Ir(III) compound exhibits a higher binding affinity than the square planar Pt(II) complex. The antibacterial activity of the compounds was evaluated on a series of Gram-negative and Gram-positive bacterial strains. In particular, the neutral Au(I) and Pt(II) complexes showed significant antibacterial activity against *Staphylococcus aureus* and *Enterococcus faecium* at very low micromolar concentrations. The cytotoxicity against a range of eukaryotic cell lines was studied and revealed that the octahedral Ir(III) complex was non-toxic, while the square-planar Pt(II) and linear Au(I) complexes displayed non-selective micromolar activity.

## Introduction

**Chart 1:**
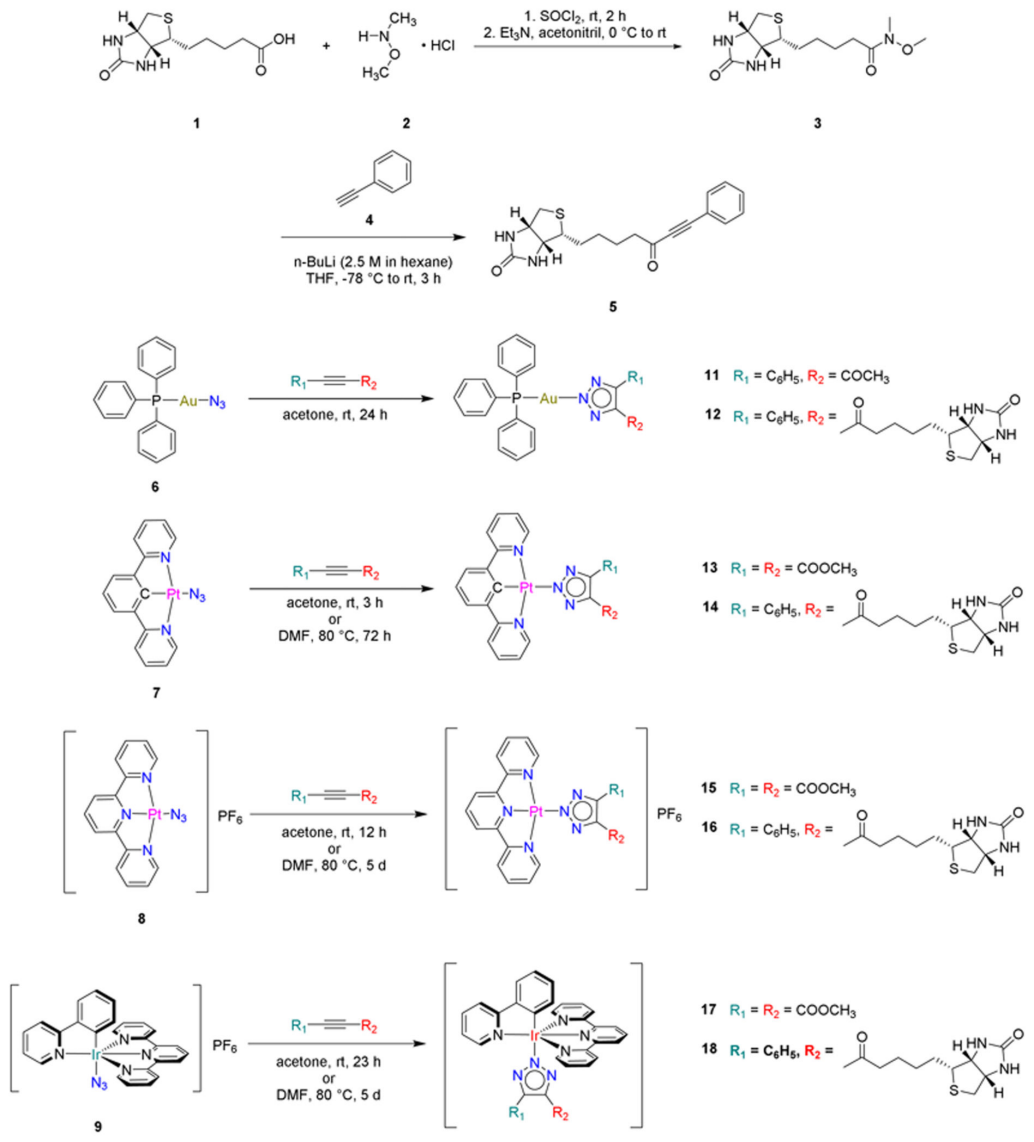
Examples of biotin-functionalized metal complexes for **(A)** bioimaging and **(B)** anticancer and antibacterial activity.

The steadily increasing emergence of drug-resistant bacterial strains requires novel approaches for antibacterial drug discovery [1, 2]. In recent years, there has been a surge in research directed at the identification of metal-based antibacterial drug candidates [3–8], a process which has been accelerated by the application of machine learning (ML) approaches to identify novel metal-ligand combinations overlooked so far by other approaches [9]. Although some large-scale screening efforts have determined the antibacterial potential of libraries composed of several hundred thousand metal complexes [7, 10], a systematic variation of metal and metal oxidation state, coordination sphere, and ligand periphery has rarely been carried out, with a good part of the available work focused on metal tricarbonyl complexes [7, 9, 11–15]. A particularly interesting modification, which has seen only very limited exploration for antibacterial drug discovery so far, is the modification of metal complexes with biotin, a biological carrier molecule that is best known for its remarkable affinity to avidin, with an exceptionally high first dissociation constant (*K*_D_) of approximately 10^-15^ M, which has led to the widespread use of the biotin-avidin system in bioanalytical applications [16–18]. Moreover, *in vitro* and *in vivo* studies indicate that the cellular uptake of biotin is facilitated by sodium-dependent multivitamin transporters (SMVTs), which are overexpressed for example in certain cancer cell lines [19–21]. So far, biotin functionalization has mostly been used for cellular targeting of Re(I), Ru(II), Rh(III), and Ir(III) luminescent markers (Chart 1A) [22–27]. In addition, heterobimetallic Fe(II)-Pt(II) compounds based on the combination of a ferrocene moiety with a square-planar Pt(alkynyl)(terpy) group with terpy = 2,2’:6’:2’’-terpyridine as well as BODIPY-Ru(terpy)_2_ conjugates have been investigated as potential photocytotoxic drug candidates (Chart 1B) [28, 29]. Furthermore, heteroleptic Ru(II) complexes with two unmodified 1,10-phenanthroline (phen) ligands and one biotin-functionalized phen were found to primarily accumulate in mitochondria [30]. Under 460 nm light irradiation, the complex generates reactive oxygen species (ROS) and deplete NADH, thus disrupting intracellular redox homeostasis in A549 cells and activating the mitochondrial apoptosis pathway. *In vivo* anti-tumor experiments demonstrated that biotinylated ruthenium(II) polypyridyl photosensitizers effectively inhibited tumor growth in A549 tumor-bearing mice under 460 nm light irradiation conditions. A biotin-functionalized 2,2’-bipyridine (bpy) ligand was also used by Garcia and Valente for conjugation to a CpRu(phosphane) half-sandwich fragment and the biological activity of the resulting compounds tested *in vitro* and *in vivo*, including zebrafish embryos [31]. On the other hand, there is only a very limited number of reports on the antibacterial activity of metal complex biotin conjugates. The first such report we could identify was published by Counter in 1960, who reported on the antimicrobial screening of a “*copper complex of biotin*” that was prepared by mixing of biotin and copper(II) sulfate under basic aqueous conditions [32]. With a sum formula of (C_10_H_15_N_2_O_3_S)_3_Cu_2_(H_2_O)_4_ proposed based on the elemental analysis, this is possibly some kind of dinuclear copper(II) paddlewheel complex [Cu_2_(µ-OOCR)_3_(H_2_O)_4_] [33], but in the absence of any further spectroscopic information, a proper structural assignment cannot be made and the antibacterial activity was only indicated by “zone of inhibition”. Only very recently, Valente and coworkers reported on Ru(II) arene half-sandwich complex with a pendant biotin group coordinated to the metal by a modified triphenylphosphine linker [34]. The type of linker between the phosphine moiety and the biotin was found to be crucial for significant antibacterial activity, with the most potent compound showing MIC values in the range of 1.5 to 12.5 µM on two different *S. aureus* strains but unfortunately differentiation between bacterial and eukaryotic cells was low, as the authors also found IC_50_ values on human cell lines in the range of 6–15 µM [34].

As highlighted by Frei, discovery of novel antibacterial metal-based drug candidates requires, beyond machine learning (ML) for the identification of novel promising substituent patterns on the ligands, a quick access to a large chemical space [9]. In that context, we have pioneered the use of the iClick (inorganic click) reaction of metal azido building blocks with functionalized alkynes [35–42] and developed a method for automated iClick synthesis based on a re-purposed HPLC autosampler [43]. In this contribution, we present a novel synthetic approach to an alkyne-functionalized biotin *via* its Weinreb amide, the preparation of metal-triazolato complexes with different functionalities in the 4- and 5-position of the five-membered ring with variation of the metal coordination sphere from linear two-coordinated Au(I) and neutral as well as monocationic square-planar [3+1] Pt(II) complexes to octahedral [3+2+1] Ir(III) compounds and compare the biological activity of compounds featuring biotin groups or not.

## Results and discussion

**Scheme 1:**
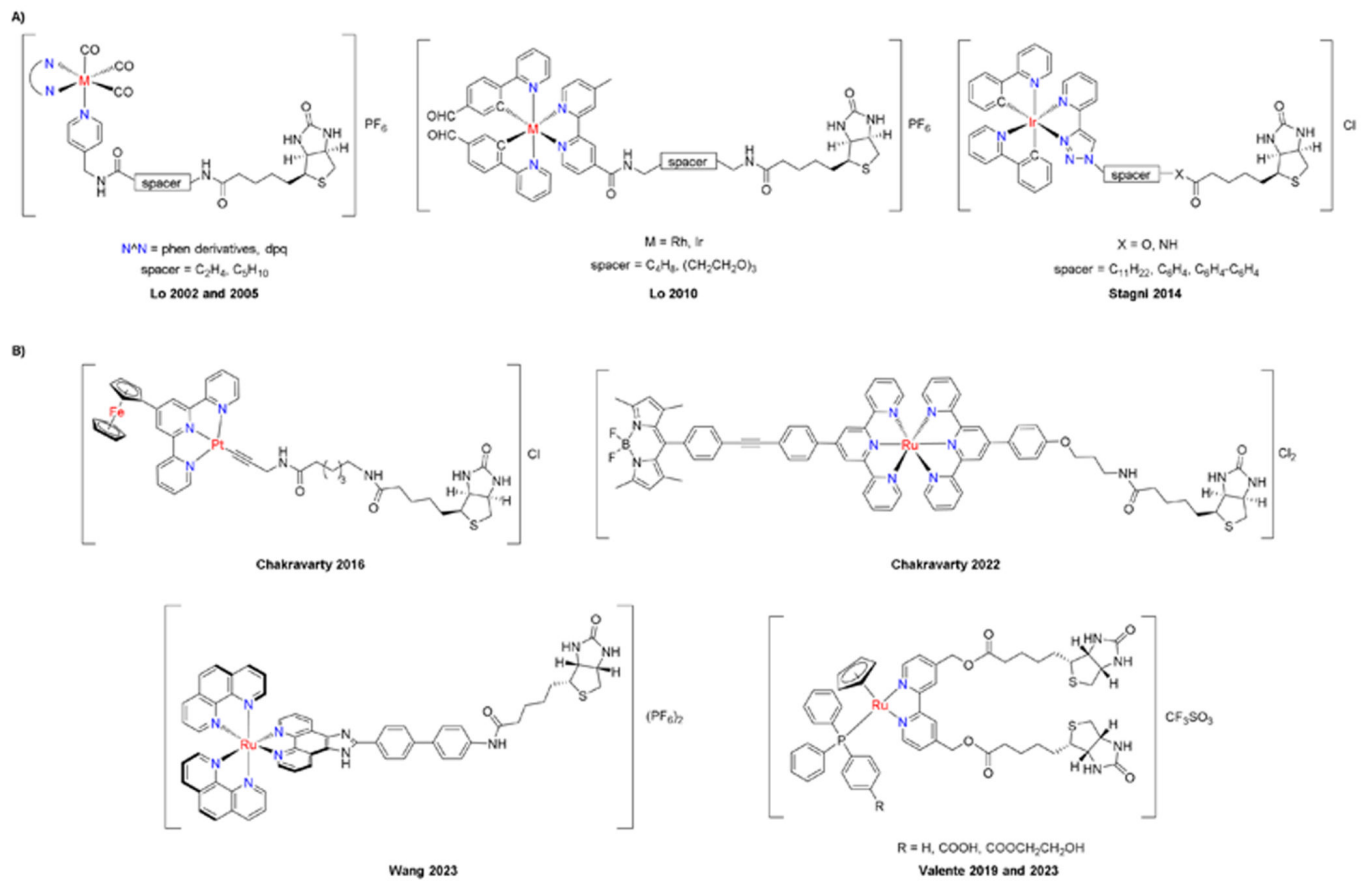
(Top) Synthesis of biotin alkyne **5** from the corresponding Weinreb amide **3**. (Bottom) iClick reaction of azido complexes **6**–**9** with electron-poor alkynes leads to triazolato complexes **11**–**18**.

The alkyne-functionalized biotin derivative **5** was synthesized from the corresponding Weinreb amide **3** by reaction with lithium phenylacetylide in tetrahydrofuran at low temperature (Scheme 1 and Scheme S1+S2) [44]. The iClick reaction partners **6**–**9** were prepared by heating a solution of the corresponding metal chlorido complexes with an excess of sodium azide in a water/acetone mixture [41, 42, 45]. Neutral gold(I) azido complex **6** reacted smoothly with biotin alkyne **5** at room temperature over 24 h, while complete conversion of the platinum(II) azido complexes **6** and **7** as well as Ir(III) azido complex **8** required stirring at 80 °C for 3–5 d.

For comparison, the azido complexes **6**–**9** were also reacted with either 4-phenylbut-3-yn-2-one or dimethyl acetylenedicarboxylate (DMAD) to give triazolato compounds **11, 13, 15**, and **17**, in which the biotin moiety is replaced by a simple methyl group. All compounds were characterized by IR and NMR spectroscopy, mass spectrometry, and CHN analysis and found to be of the highest possible purity required for biological testing (Figure S1 to S34). For the triazolato complexes, ^1^H, ^13^C, ^31^P, and ^195^Pt NMR spectra show only one set of signals, indicating that only the N2-coordinated triazolato ligand is present in the complex, as previously reported [41, 42]. In the ^31^P NMR spectra of Au(I) complexes **11** and **12**, there is a characteristic signal observed at 30.56 and 30.52 ppm, respectively, indicating the successful formation of the triazolato ring and its rearrangement to an exclusively N2-coordinated complex, as reported previously [45]. Similar, in the ^1^H NMR spectra of Pt(II) complexes **13**–**16**, there is a characteristic shift observed for the outer pyridine wing protons H6/H6’’ towards higher values upon triazolato ring formation. Additionally, the ^195^Pt NMR spectra show distinct differences in the chemical shifts of neutral Pt(II) dpb complexes **13** and **14** at -3661 [40] and -3686 ppm compared to the monocationic Pt(II) terpy complexes **15** and **16** with peaks at -2671 [42] and -2699 ppm, respectively. These results are in line with earlier studies on complexes **13** and **15**, highlighting the crucial influence of the substituents in 4- and 5-position of the triazolato ring in determining the formation of the N2-coordinated heterocyclic ligand. Likewise, upon iClick reaction, the characteristic ppy-H6 signal shifts from 9.32 ppm in Ir(III) azido complex **9** to 8.88 ppm for triazolato compound **17**, while in biotin-functionalized complex **18**, the ppy-H6 is observed at 9.26 ppm, indicating a distinct chemical environment compared to **17** as previously reported [46].

### Lipophilicity of the biotin complexes

As the lipophilicity of a compound significantly influences its cellular uptake and pharmacokinetic properties, the distribution coefficient log*P* was determined by the shake-flask method for organic biotin-alkyne **5** as well as metal complexes **11**–**18** (Table 1) [47, 48]. The charged triazolato complexes **15**–**18** are quite hydrophilic, with log*P* values in the range of 0.14 to –1.80, irrespective of the nature of the metal center – square planar Pt(II) *vs*. octahedral Ir(III). In the case of both metals, introduction of the biotin moiety to the periphery of the triazolato ligand led to an increase in the log*P* value by about one order of magnitude, thus making the biotin conjugates more lipophilic relative to the control compounds. Due to their low solubility and high lipophilicity, log*P* values could not be determined for neutral complexes **11**–**14**, as significant UV/Vis absorption was only observed in the *n*-octanol but not the aqueous phase. Consequently, a quantitative determination of log*P* values is not possible for these compounds.

### Avidin/streptavidin binding

**Figure 1:**
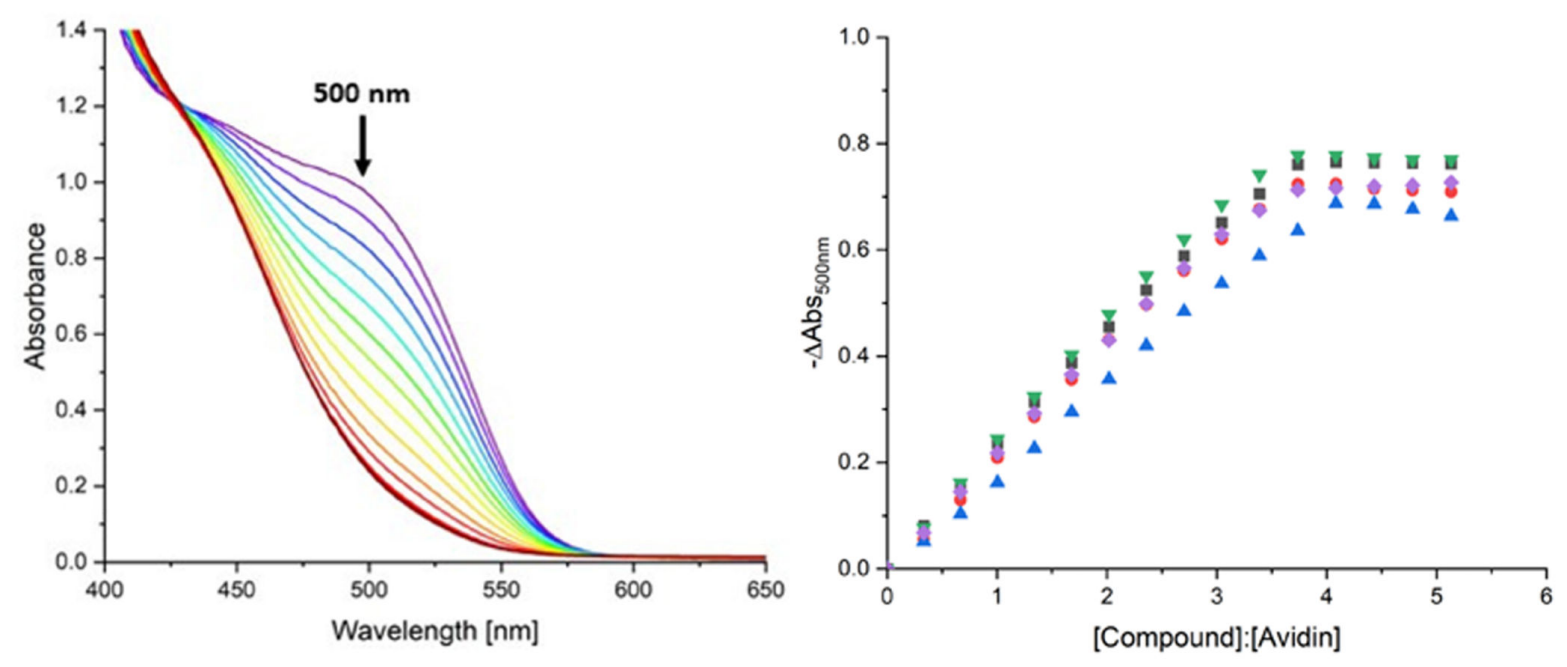
(Left) Changes in the UV/Vis absorption spectrum upon titration of the HABA/avidin adduct with **12**. (Right) -ΔAbs_500nm_ *vs*. ratio of c(compound):c(avidin) for: biotin (black trace), [Au(PPh_3_)(triazolato^C6H5,biotin^)] **12** (red trace), [Pt(dpb)(triazolato^C6H5,biotin^)] **14** (blue trace), [Pt(triazolato^C6H5,biotin^)(terpy)]PF_6_ **16** (green trace), and [Ir(ppy)(triazolato^C6H5,biotin^)(terpy)]PF_6_ **18** (purple trace).

The avidin binding of biotin-alkyne **5** as well as triazolato complexes **11**–**18** was studied with the commercial HABA assay [16, 24]. Since the affinity of avidin for HABA (*K*_*d*_ = 6 × 10^-6^ M) is much lower than that for biotin (*K*_d_ = ~10^-15^ M), the competitive binding leads to displacement of HABA from the protein upon titration with biotin, resulting in a decrease in the absorbance at 500 nm. An example of these changes in the UV/Vis absorption spectrum are shown for the titration of a HABA/avidin mixture with the neutral biotin-functionalized Au(I) triazolato complex **12** (Figure 1 left). Similar changes were also observed for unmodified biotin, biotin-alkyne conjugate **5**, and metal compounds **12, 14, 16**, and **18**. On the other hand, complexes **11, 13, 15**, and **17** lacking the biotin moiety only showed signs of non-specific protein binding (Figure S35–S45). From a plot of the change in absorbance at 500 nm *vs*. the compound-to-avidin ratio, a clear 4:1 stoichiometry can be deduced, as expected due to the non-cooperative binding to avidin (Figure 1 right).

In addition, binding to the streptavidin monomer, which only features a single biotin binding site, was studied by isothermal titration calorimetry (ITC). Due to the low aqueous solubility of the neutral complexes in terms of the required biotin concentration, only the hydrophilic cationic Pt(II) and Ir(III) complexes **15, 16**, and **18** could be investigated. Furthermore, due to a lower limit of the molar concentration that can be used in the assay, the dissociation constant of the functionalized biotin-alkyne **5** could only be estimated to be < 1 × 10^-9^ M. However, this value correlates well with literature data for unsubstituted biotin, which has a dissociation constant of 1.0 × 10^-15^ M [17]. The biotin-functionalized Ir(III) complex **18** exhibited a dissociation constant of (4.5 ± 1.8) × 10^-8^ M, whereas Pt(II) conjugate **16** binds less tightly, with *K*_d_ = (1.3 ± 0.4) × 10^-7^ M. In contrast, the non-functionalized Pt(II) complex **15** did not bind to streptavidin due to a lack of the biotin moiety, as expected (Figure S46–S49).

### Cell viability and bacterial growth inhibition studies

The antibacterial activity of biotin-alkyne **5** as well as triazolato complexes **11**–**18** was studied in *Acinetobacter baumannii, Escherichia coli, Klebsiella pneumoniae, Pseudomonas aeruginosa, Enterococcus faecium, Staphylococcus aureus* by a rapid screen broth microdilution assay (Figure S50 and S51). Activity was observed in *E. faecium*, and *S. aureus* and the minimum inhibitory concentrations (MICs) for these strains were determined with a MIC broth microdilution assay (Table 2). No significant antibacterial activity was observed in the Gram-negative bacterial strains for any of the tested compounds. However, neutral Au(I) complexes **11** and **12** as well as uncharged Pt(II) compound **14** showed MIC values in the 0.5–10 µM range in the Gram-positive *E. faecium* and *S. aureus*, irrespective of the presence of a biotin moiety. Generally, the Au(I) compounds appear to be slightly more potent than the Pt(II) compound, but differences are at the limit of the method. The charged complexes **15**–**18** did not show any antibacterial effect, irrespective of metal coordination sphere and triazolato substituent pattern, while the lack of potency of neutral Pt(II) compound **13** currently cannot be rationalized, as it has a similar lipophilicity as the other three active complexes. The lack of susceptibility of the Gram-negative bacterial strains is typical due to their extensive defences. For example, the additional outer membrane in the Gram-negative cell wall which is difficult to penetrate for most compounds [49, 50].

To further evaluate the potential cytotoxicity of the title compounds, their antiproliferative activity was tested against non-malignant BEASB2 (non-cancer lung), HaCat (non-cancer skin), and RPE1 (retinal pigment epithelial cells) as well as cancerous HCT116 (colorectal cancer) and HeLa (cervical cancer) cell lines (Table 3). To determine the relative viability, after incubation cells were fixed and stained with DAPI and the number of nuclei per well determined using a CeligoTM Image Cytometer (Figure S52 to S60). Generally, there was little differentiation between cell lines for a single compound, as they were mostly either responsive (IC_50_ < 15 µM) or essentially non-cytotoxic (IC_50_ > 50 µM). The two Au(I) compounds **11** and **12** turned out to be highly toxic to all cell lines studied, with IC_50_ values in the range of 2–8 µM, irrespective of the presence of the biotin moiety. The neutral Pt(II) compound **13**, with the two methyl ester substituents on the triazolato ligand, did not show any biological activity. This is interestingly also reflected in the antibacterial activity. The other three Pt(II) complexes (**14–16**) mostly showed moderate cytotoxicity, with IC_50_ values in the range of 5–15 µM, except against BEAS2B cells, which did not respond to **15** even at 50 µM. The two Ir(III) complexes **17** and **18**, although also bearing a positive charge, were essentially inactive, with IC_50_ values consistently above 35 µM, irrespective of the functional groups on the triazolato ligand. The alkyne-functionalized biotin **5**, on the other hand, turned out to be highly cytotoxic, with IC_50_ values in a narrow range of 2–4 µM, depending on the cell line. However, the compounds **13, 17**, and **18** which are the least cytotoxic on eukaryotic cells are also the ones which do not exhibit any antimicrobial activity (Table 2), while complexes **11** and **12** with low to sub-micromolar MIC values are also highly cytotoxic in all five eukaryotic cell lines tested.

## Conclusion

A series of biotin-functionalized metal triazolato complexes, including linear Au(I), square-planar Pt(II), and octahedral Ir(III) complexes was prepared by iClick reaction from the corresponding azido compounds and different internal alkynes. Among the Pt(II) compounds, a neutral *vs*. cationic charge was adjusted by use of a 1,3-di(2-pyridyl)phenide (dpb) *vs*. 2,2’:6’,2’’-terpyridine (terpy) tridentate ligand. While the Au(I) azido starting compound underwent smooth iClick reaction at room temperature with the new biotin-alkyne, the Pt(II) and Ir(III) precursors required extended heating, although the biotin moiety was not affected by the rather high reaction temperature. The avidin/streptavidin binding of the title compounds was compared to those lacking the biotin moiety using the HABA assay as well as isothermal titration calorimetry (ITC) and was not influenced by the presence of the metal moiety. Although a lack of differentiation between bacterial and eukaryotic cell lines was observed, some important conclusions can still be drawn from the bioactivity data on general structure-activity relationships. First, the biotin substituent increases the lipophilicity of the conjugates by about one order of magnitude but does not have a significant influence on the biological activity, as no effect is seen on neither MIC nor IC_50_ values. Second, the linear Au(I) compounds are highly active, at times with sub-micromolar MIC values, but their activity is quite non-specific, as they are toxic on both bacterial and eucaryotic cells. The octahedral Ir(III) complexes, on the other hand, lack any biological activity but might be useful as luminescent probes [46]. Finally, the square-planar Pt(II) complexes are intermediate between the two other classes of compounds and their antibacterial *vs*. anticancer activity seems to be controlled by a fine balance of charge and triazolato substitution pattern, which will require further examination to work out clear trends.

## Supplementary Material

Figure S1

## Figures and Tables

**Figure 2:**
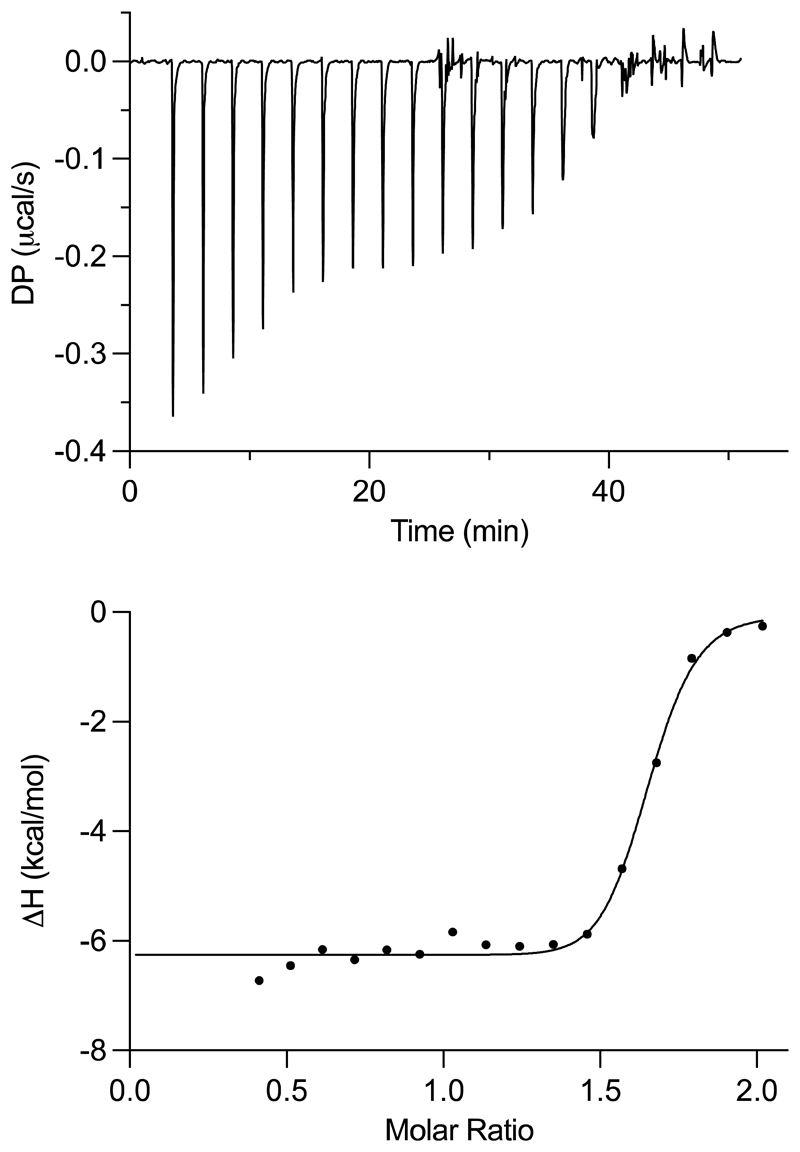
(Top) Unprocessed ITC thermogram and (bottom) binding isotherm from the integrated thermogram fit for the binding of Ir(III) compound **18** (200 µM) to streptavidin (20 µM). Circles indicate the integrated heat, and the curve represents the best fit.

**Table 1:** Log*P* values of compounds **5** and **11**–**18** determined with the shake-flask method.

Compound	11	12	13	14	15	16	17	18	5
**Metal**	Au	Au	Pt	Pt	Pt	Pt	Ir	Ir	n/a
**Charge**	0	0	0	0	+1	+1	+1	+1	---
**Biotin**	-	+	-	+	-	+	-	+	+
**LogP**	highly lipophilic^a^	highly lipophilic^a^	highly lipophilic^a^	highly lipophilic^a^	-1.80	-0.78	-1.02	0.14	highly lipophilic^a^

a No UV/Vis signal of the complexes was detectable in the aqueous phase; therefore, no concentration ratio could be calculated.

**Table 2:** Minimum inhibitory concentration (MIC) in μM for compounds **5** and **11**–**18**.

Compound	11	12	13	14	15	16	17	18	5	Control
**Metal**	Au	Au	Pt	Pt	Pt	Pt	Ir	Ir	n/a	n/a
**Charge**	0	0	0	0	+1	+1	+1	+1	0	n/a
**Biotin**	-	+	-	+	-	+	-	+	+	n/a
***A. baumannii*** ***(Gram negative)***	100	50	>200	>200	>200	>200	>200	>200	>200	0.31^a^
***E. coli*** ***(Gram negative)***	>200	>200	>200	>200	>200	>200	>200	>200	>200	12.5^b^
***K. pnuemoniae*** ***(Gram negative)***	>200	>200	>200	>200	>200	>200	>200	>200	>200	<0.16^a^
***P. aeruginosa*** ***(Gram negative)***	>200	>200	>200	>200	>200	>200	>200	>200	>200	100^a^
***E. faecium*** ***(Gram positive)***	0.625–5	0.625–2.5	>200	5–10	>200	>200	>200	100	200	0.5^c^
***S. aureus*** ***(Gram positive)***	2.5	1.25	200	12.5	>200	>200	>200	100	>200	12.5^b^

a Ciprofloxacin

b Carbenicillin

c Vancomycin

The data for the Gram-negative bacterial strains is from a rapid screen without replicates while exact MIC values were from a broth microdilution assay.

**Table 3:** IC_50_ values for **5** and **11**–**18** in µM determined after 48 h.

Compound	11	12	13	14	15	16	17	18	5
**Metal**	Au	Au	Pt	Pt	Pt	Pt	Ir	Ir	n/a
**Charge**	0	0	0	0	+1	+1	+1	+1	0
**Biotin functionalization**	-	+	-	+	-	+	-	+	+
***BEAS2B***	2.5	2.7	>50	6.7	>50	8.3	>50	>50	3.6
***HACAT***	6.1	6.6	>50	10.2	15.4	14.1	>50	>50	3.3
**RPE1**	2.7	3.6	>50	7.4	4.9	5.2	35.0	39.7	2.4
***HCT116***	5.5	7.2	>50	9.4	6.0	6.8	>50	>50	4.0
***HeLa***	3.5	3.8	>50	8.2	15.9	13.3	>50	>50	3.3

## Data Availability

All data relevant to this publication is included in the manuscript or the supporting information.

## List of abbreviations

BODIPY: 4,4-difluoro-4-bora-3*a*,4*a*-diaza-*s*-indacen
bpy: 2,2’-bipyridine
DAPI: 4′,6-diamidino-2-phenylindole
DMAD: dimethyl acetylenedicarboxylate
dpb: 1,3-di(2-pyridyl)benzene
HABA: 4’-hydroxyazobenzol-2-carboxylic acid
IR: infrared
ITC: isothermal titration calorimetry
MIC: minimum inhibitory concentration
NADH: nicotinamide adenine dinucleotide
NMR: nuclear magnetic resonance
ppy: 2-phenylpyridine
ROS: reactive oxygen species
SMVT: sodium-dependent multivitamin transporters
terpy: 2,2’:6’,2’’-terpyridine
UV/Vis: ultraviolet/visible
