# Supplementary material for: Antibacterial activity of Au(I), Pt(II), and Ir(III) biotin conjugates prepared by the iClick reaction: Influence of the metal coordination sphere on the biological activity
Source: J Biol Inorg Chem. Author manuscript; Available in PMC 2024 Oct 10. (PMC7616682; doi:10.1007/s00775-024-02073-x)
Supplement: Figure S1 [file EMS199144-supplement-Figure_S1.pdf]

Antibacterial activity of Au(I), Pt(II), and Ir(III) biotin conjugates prepared by the iClick reaction: Influence of the metal coordination sphere on the biological activity

Dominik Moreth,[a] Lars Stevens-Cullinane,[b,c] Thomas W. Rees,[b] Victoria V.L. Müller,[a] Adrien Pasquier,[d] Ok-Ryul Song,[d] Scott Warchal,[d] Michael Howell,[d] Jeannine Hess,[b,c] and Ulrich Schatzschneider[a*]

[a] Institut für Anorganische Chemie, Julius-Maximilians-Universität Würzburg, Am Hubland, D-97074 Würzburg, Germany

[b] Biological Inorganic Chemistry Laboratory, The Francis Crick Institute, London NW1 1AT, United Kingdom

[c] Department of Chemistry, King's College London, Britannia House, 7 Trinity Street, London SE1 1DB, United Kingdom

[d] High Throughput Screening Science and Technology Platform, The Francis Crick Institute, London NW1 1AT, United Kingdom

* Corresponding author: ulrich.schatzschneider@uni-wuerzburg.de;
Tel: +49 931 31 83636; Fax: +49 931 31 84605

Supporting Information

TABLE OF CONTENTS

Experimental Section 3

Synthesis of *N*-methoxy-*N*-methyl-5-[(3aS,4S,6aR)-2-oxohexahydro-1*H*-thieno-[3,4-d]imidazol-4-yl]pentanamide 3 .... Fehler! Textmarke nicht definiert.

Synthesis of 7-[(3aS,4S,6aR)-2-oxohexahydro-1H-thieno-[3,4-d]imidazol-4-yl]-1-phenyl-1-heptyn-3-one 5 .... Fehler! Textmarke nicht definiert.

Synthesis of [AuCl(PPh_3)] 19[4] 5

Synthesis of [Au(N_3)(PPh_3)] 6 8

Synthesis of [Au(triazolato^{C6H5,COCH_3})(PPh_3)] 11 11

Synthesis of [Au(triazolato^{C6H5,biotin})(PPh_3)] 12 14

Synthesis of [Pt(triazolato^{C6H5,biotin})(dpb)] 14 18

Synthesis of [Pt(triazolato^{C6H5,biotin})(terpy)]PF_6 16 23

Synthesis of [Ir(triazolato^{C6H5,biotin})(ppy)(terpy]PF_6 18 27

Log*P* measurements 31

HABA/Avidin Assay 32

Isothermal titration calorimetry 37

Antibacterial activity assay 40

Cell viability studies 43

References 53

Experimental Section

General remarks. Unless mentioned otherwise, all chemicals were purchased from commercial vendors such as SIGMA-ALDRICH, STREM, ABCR, and ROTH. Reactions which require the exclusion of moisture and/or oxygen were conducted under argon in oven-dried Schlenk glassware. Thin layer chromatography (TLC) was performed on TLC plates ALUGRAM® Xtra SIL G/UV254 with a thickness of 200 µm from *MACHEREY-NAGEL.* For column chromatography, silica gel 60 with a particle size of 40-63 µm was used. **Instrumentation.** NMR spectra were recorded on a BRUKER Avance 400 Nanobay spectrometer (^{1}H: 400.47 MHz; ^{13}C: 100.7 MHz, ^{19}F: 376.7 MHz, ^{31}P: 162.11 MHz ^{195}Pt: 85.79 MHz) or a BRUKER Avance Neo I 500 spectrometer (^{1}H: 500.13 MHz; ^{13}C: 125.67 MHz). Chemical shifts δ in ppm represent a downfield shift relative to tetramethylsilane (TMS) and were referenced relative to the signal of residual non-deuterated solvent.[1] The ^{195}Pt NMR shifts are reported relative to 1.2 M $Na_2[PtCl_6]$ in D_2O, respectively. Coupling constants J are given in Hz. Peak multiplicities are indicated as singlet (s), doublet (d), doublet of doublet (dd), doublet of doublet of doublet (ddd), doublet of triplet (dt), triplet (t), and multiplet (m), respectively. Spectra were analysed with BRUKER TopSpin version 4.09. Mass spectra were recorded on a THERMOFISCHER *Exactive Plus* spectrometer with an *Orbitrap* mass analyser and an ESI (resolution R = 70000, sheath gas flow rate 15 L min^{-1}, aux gas flow rate 5 L min^{-1}, spray voltage 3.80 kV, capillary temperature 320 °C, S-lens level 50.0, and aux gas heater temperature 50 °C) or APCI (resolution R = 140000, sheath gas flow rate 2 L min^{-1}, aux gas flow rate 10 L min^{-1}, spray voltage 3.80 kV, capillary temperature 320 °C, S-lens level 50.0, and aux gas heater temperature 400 °C) ion source. IR spectra of pure solid samples were collected on a *Jasco FT/IR-4100 FT-IR spectrometer* fitted with a smart iTR ATR accessory in the range of 4000 to 700 cm^{-1} with 32 scans per sample. Signal intensities are reported as very strong (vs), strong (s), medium (m), weak (w), or broad (br). The elemental composition of the compounds was determined with *an Elementar Vario MicroCube* CHN analyser. Addition of V_2O_5 was usually required to obtain proper results. UV/Vis absorption spectra were recorded using an Agilent 8453 diode array spectrophotometer with quartz cuvettes (d = 1 cm). Bacterial assay results were determined by measuring the absorption at 600 nm on a BMG Clariostar plate reader. An Echo 550 Acoustic Liquid Handler, Integra® Viafill Bulk dispenser and Celigo™ Image Cytometer were used for human cell viability assays.

Synthesis procedures. Azido complexes **6–9** and triazolato complexes **13**, **15** and **17** were prepared as recently published.[2-5]

Caution! Heavy metal azido complexes are potentially explosive. Although no problems were encountered during the preparations reported in this work, due care and attention with appropriate precautions should be taken in their synthesis and handling. Generally, not more than 100 mg of compound should be prepared, and heating of the solid compounds avoided.

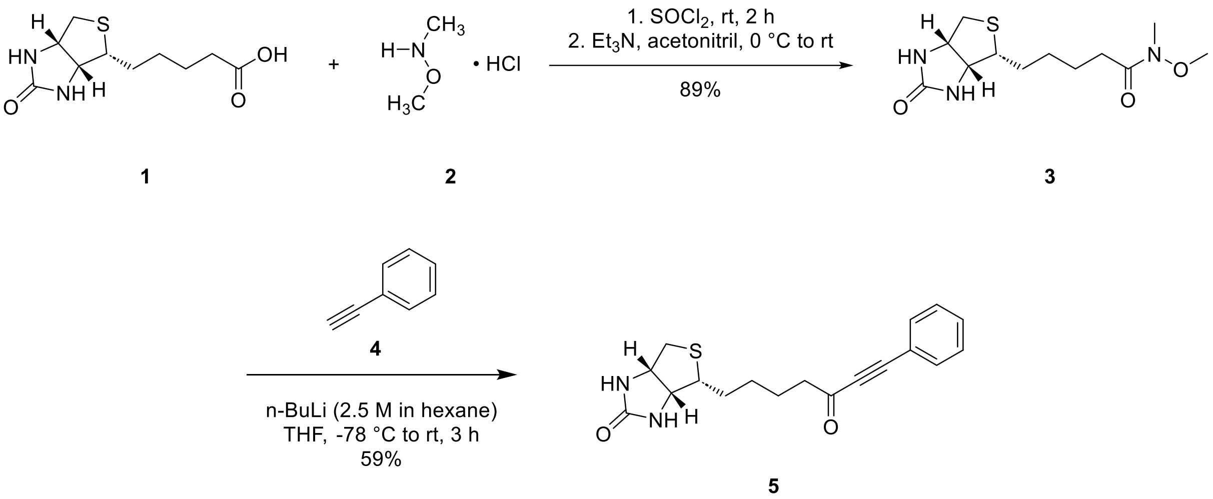

Scheme S1: Synthesis of alkynone-functionalized biotin **5.**

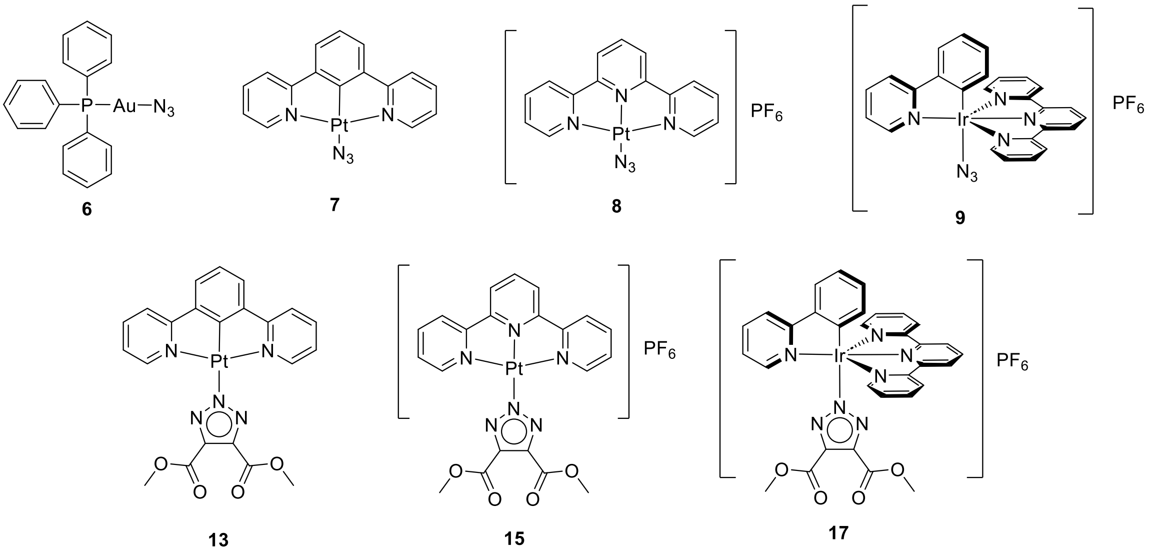

Scheme S2: Metal azido complexes **6**–**9** and triazolato compounds **13, 15** and **17** studied in this work prepared by literature procedures.[3, 4]

Synthesis of [AuCl(PPh_3)] 19 [2]

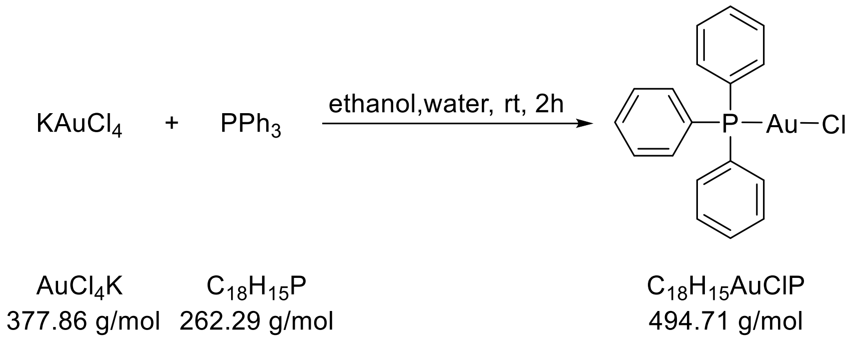

Potassium tetrachloroaurate(III), (252 mg, 0.67 mmol) was dissolved in a mixture of ethanol (10 mL) and water (1 mL) to give a clear yellow solution. Then, triphenylphosphine (354 mg, 1.35 mmol) was dissolved in a mixture of ethanol (10 mL) and water (1 mL). The two solutions were mixed to afford a yellow precipitate immediately. The suspension was stir at room temperature for 2 h, during which the yellow precipitate turned white, and the solution became colorless. The white solid was then filtered off, washed with diethyl ether (4 × 3 mL), and dried under vacuum. Yield: 89% (296 mg, 0.60 mmol). **IR** (ATR): $\tilde{\nu}$ = 3073 (w), 1479 (m), 1443 (s), 1312 (w), 1180 (w), 1101 (s), 747 (s), 713 (s) cm^{-1}; **^{1}H NMR** (400.47 MHz, DMSO-d_6): δ = 7.67–7.51 (m, 15H, C_6H_5) ppm; **^{13}C NMR** (100.71 MHz, DMSO-d_6): δ = 133.80 (d, $^{2}J_{C2/C6,P}$ = 13.7 Hz, phenyl-C2/C6), 132.32 (d, $^{4}J_{C4,P}$ = 2.6 Hz, phenyl-C4), 129.67 (d, $^{3}J_{C3/C5,P}$ = 11.8 Hz, phenyl-C3,C5), 128.21 (d, $^{1}J_{C1,P}$ = 62.2 Hz, phenyl-C1) ppm; **^{31}P NMR** (162.11 MHz, DMSO-d_6): δ = 32.68 ppm; **Elemental analysis**(%) calcd. for $C_{18}H_{15}AuClP$: C 43.70, H 3.06; found: C 44.06, H 2.90.

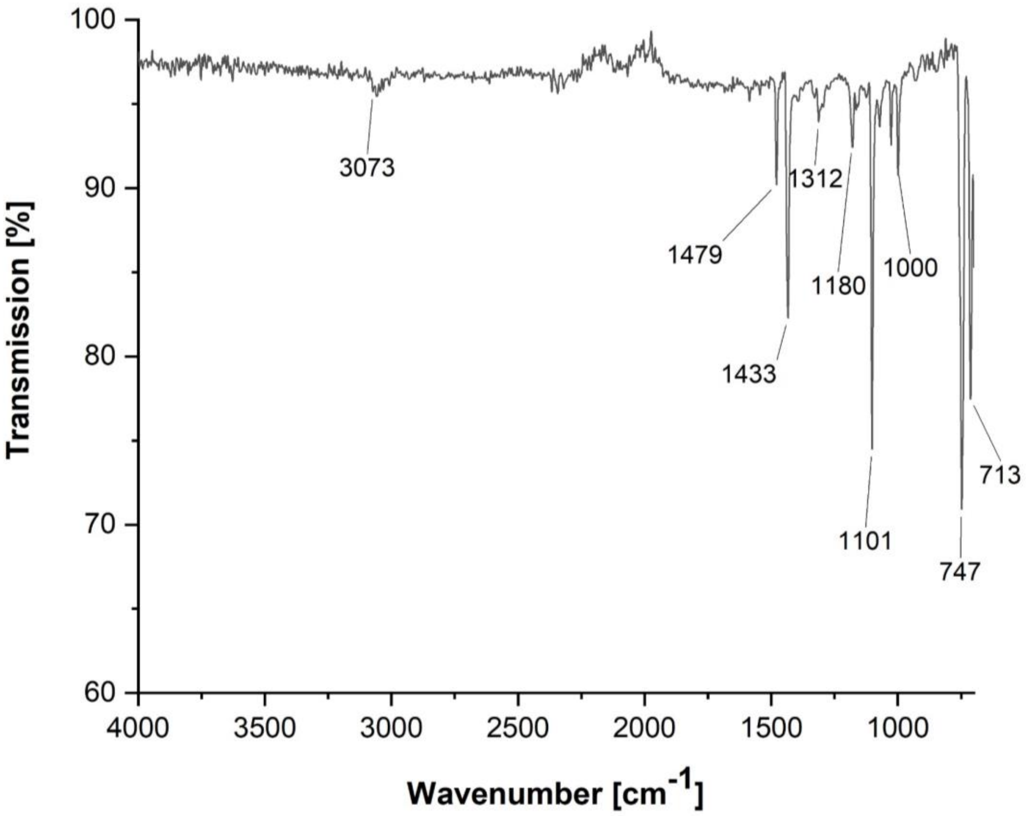

Figure S1: ATR IR spectrum of **20.**

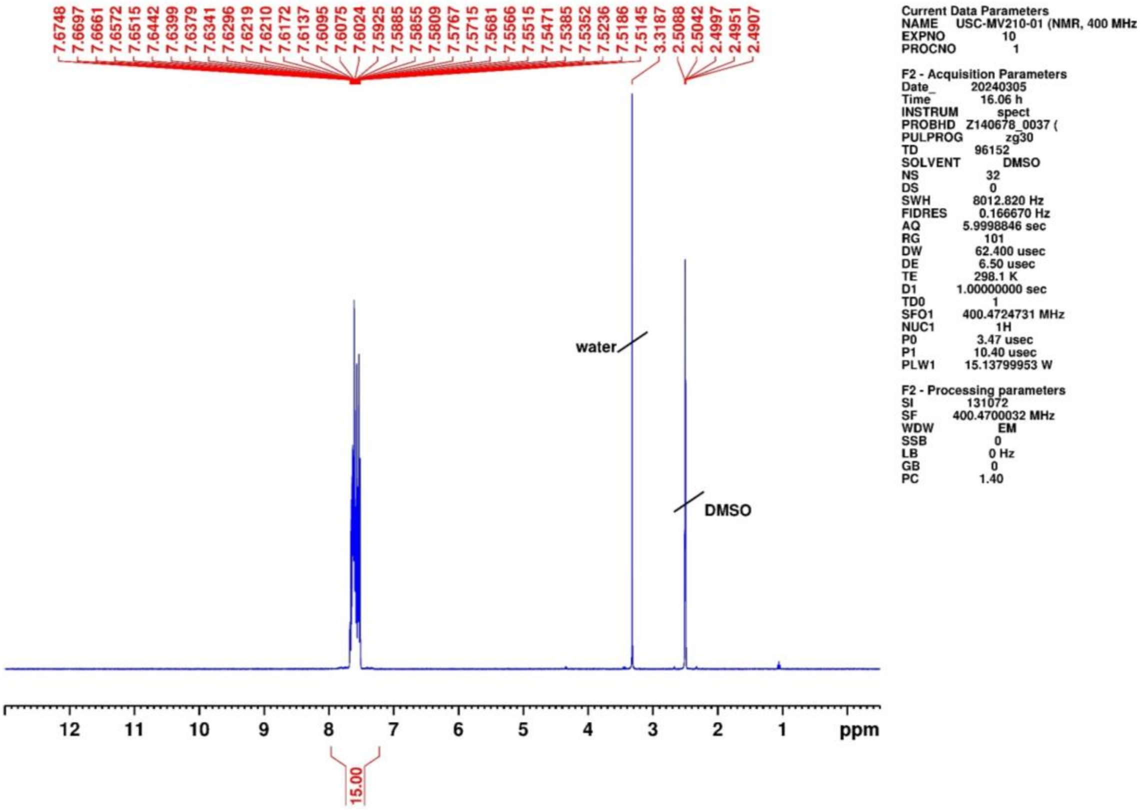

Figure S2: 400.47 MHz ^{1}H NMR spectrum of **20** in DMSO-d_{6}.

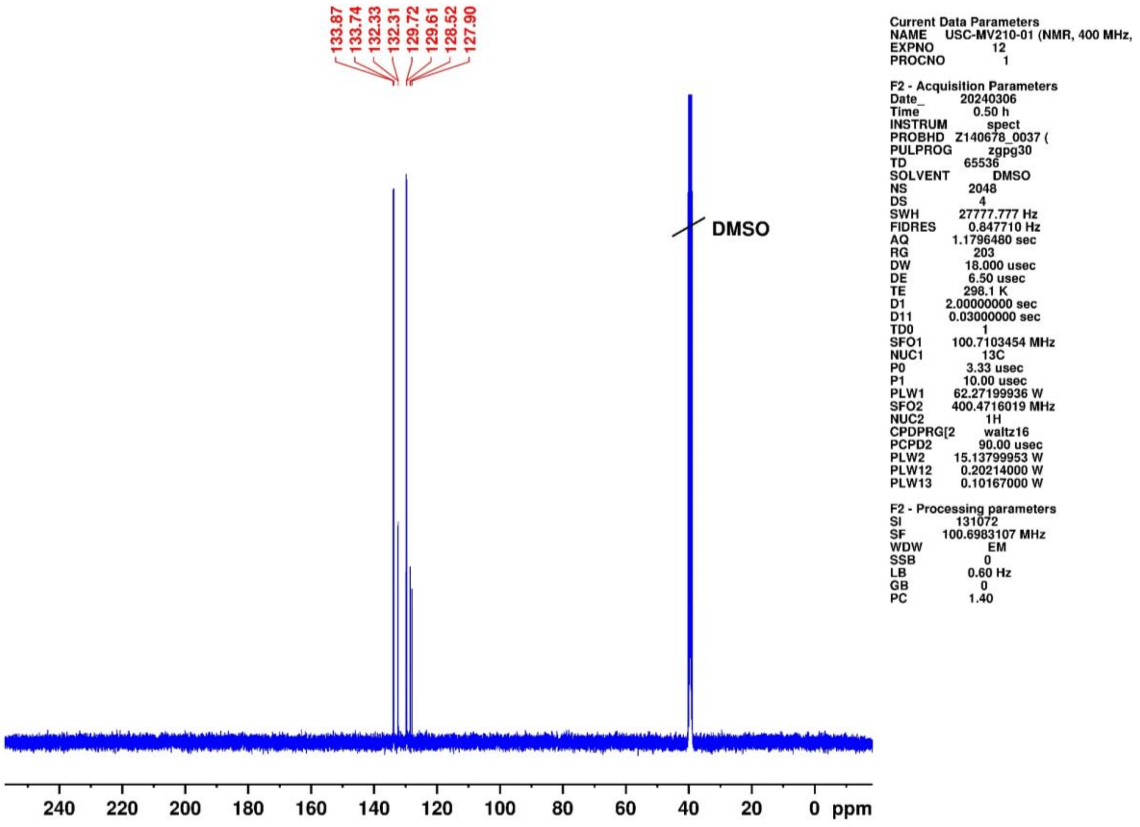

Figure S3: 100.71 MHz ^{13}C NMR spectrum of **20** in DMSO-d_6.

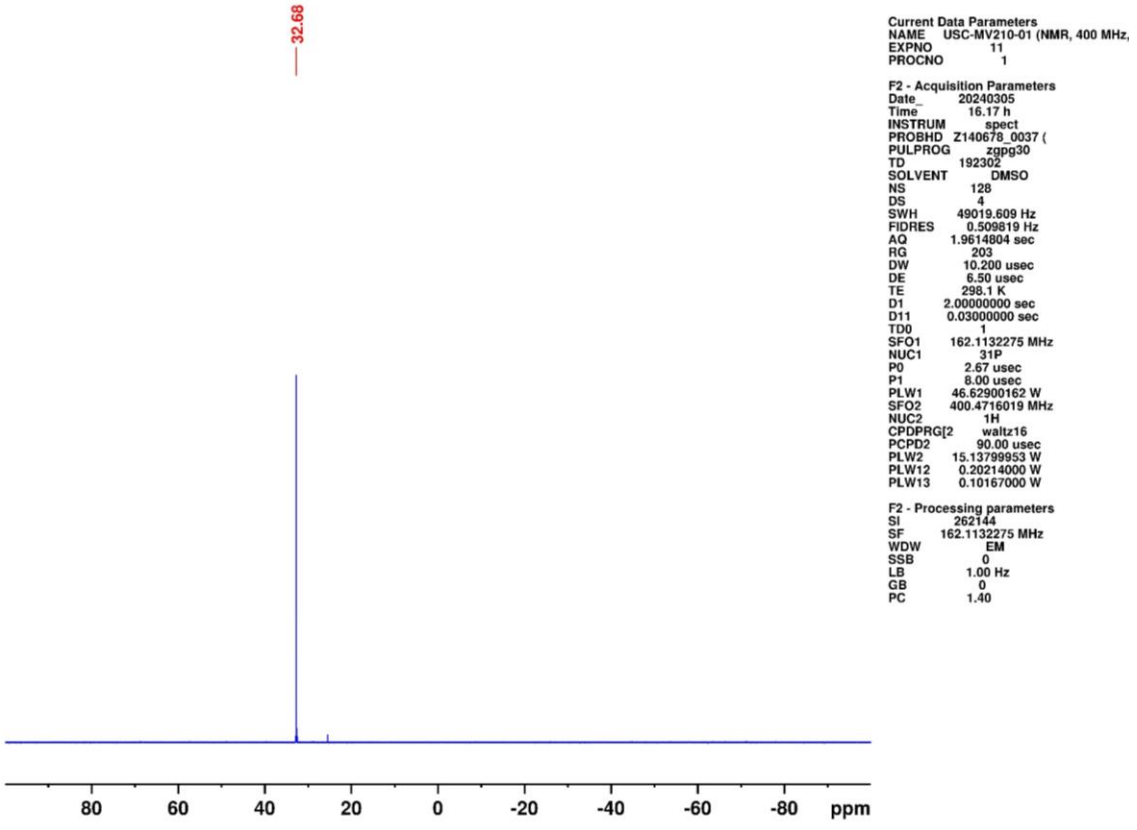

Figure S4: 162.11 MHz ^{31}P NMR spectrum of **20** in DMSO-d_6.

Synthesis of $[Au(N_3)(PPh_3)]$ 6[2]

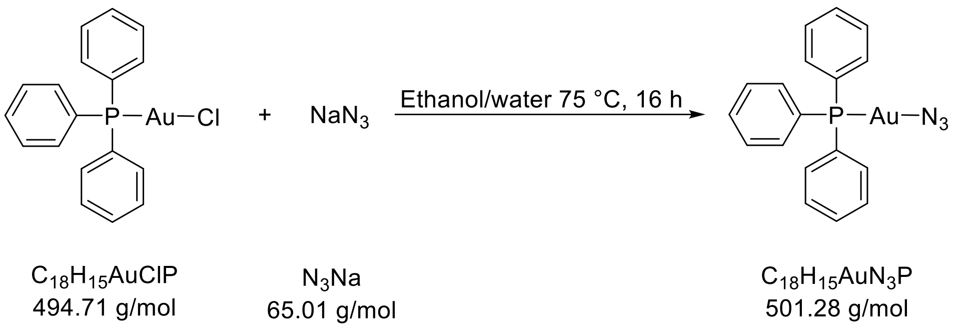

$[AuCl(PPh_3)]$ (174 mg, 0.35 mmol) was dissolved in ethanol (100 mL) at 75 °C to give a clear colorless solution. Then, sodium azide (228 mg, 3.50 mmol) dissolved in water (5 mL) was added into the colorless solution. After stirring at 75 °C for 16 h, the solvent was removed from the mixture to give the white solid, which was filtered off, washed with water (3 × 10 mL), and dried under vacuum. Yield: 92% (162 mg, 0.32 mmol). **IR** (ATR): $\tilde{\nu}$ = 3057 (w), 2049 (s), 1476 (m), 1434(s), 1279 (m), 1100 (s), 998 (w), 749 (s), 711 (s) cm^{-1}; **^{1}H NMR** (400.47 MHz, DMSO-d_6): δ = 7.67–7.51 (m, 15H, C_6H_5) ppm; **^{13}C NMR** (100.71 MHz, DMSO-d_6): δ = 133.85 (d, $^{2}J_{C2/C6,P}$ = 13.6 Hz, phenyl-C2/C6), 132.30 (d, $^{4}J_{C4,P}$ = 2.5 Hz, phenyl-C4), 129.63 (d, $^{3}J_{C3/C5,P}$ = 11.7 Hz, phenyl-C3/C5), 128.09 (d, $^{1}J_{C1,P}$ = 62.5 Hz, phenyl-C1) ppm; **^{31}P NMR** (162.11 MHz, DMSO-d_6): δ = 30.30 ppm; **Elemental analysis**(%) calcd. for $C_{18}H_{15}AuN_3P$: C 43.13, H 3.02, N 8.38; found: C 43.33, H 2.81, N 8.75.

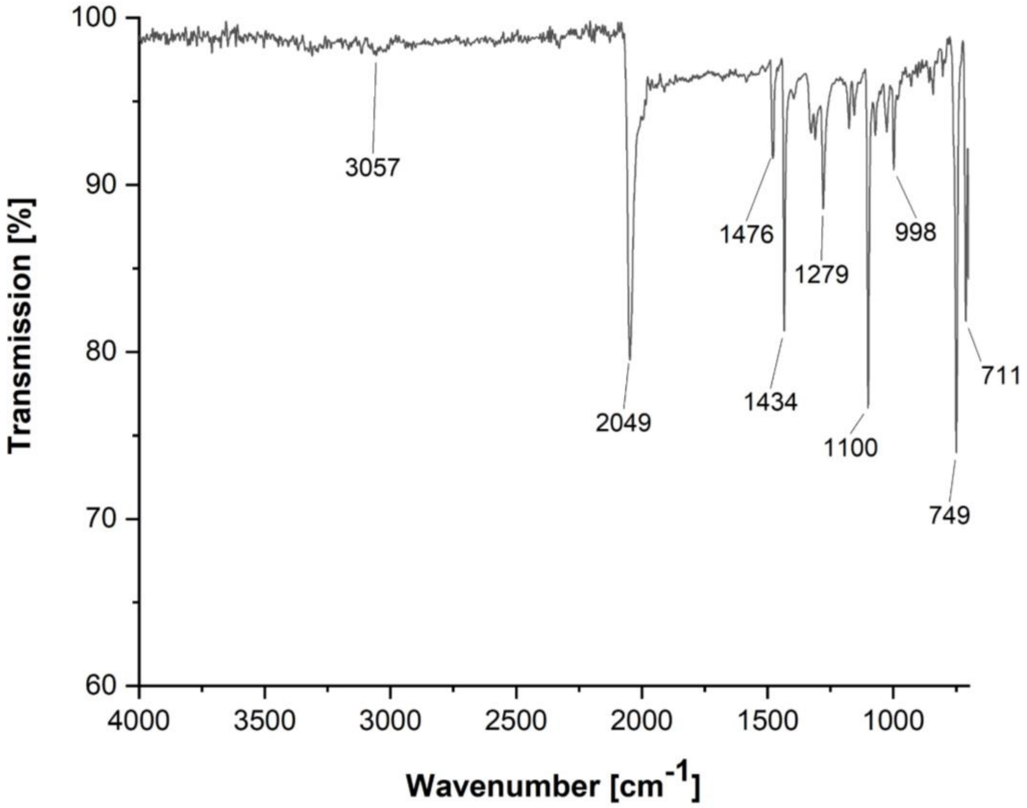

Figure S5: ATR IR spectrum of **6**.

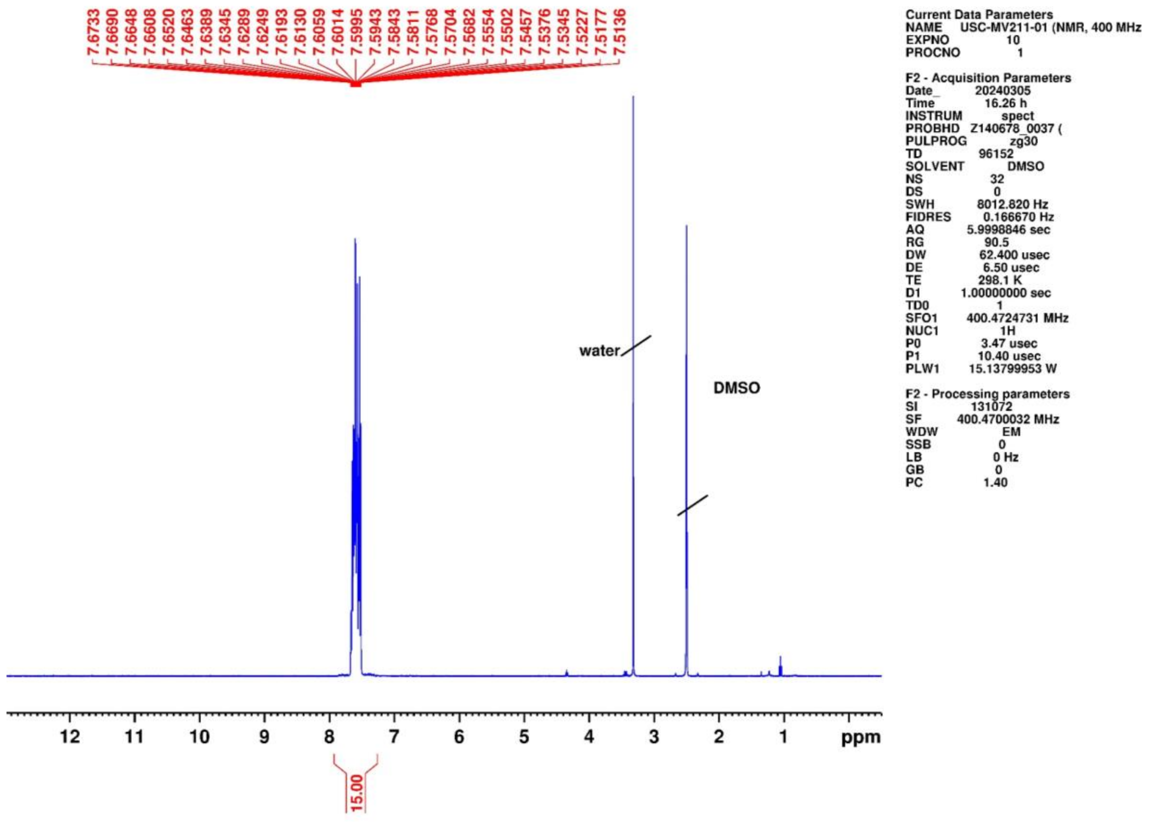

Figure S6: 400.47 MHz ^{1}H NMR spectrum of **6** in DMSO-d_6.

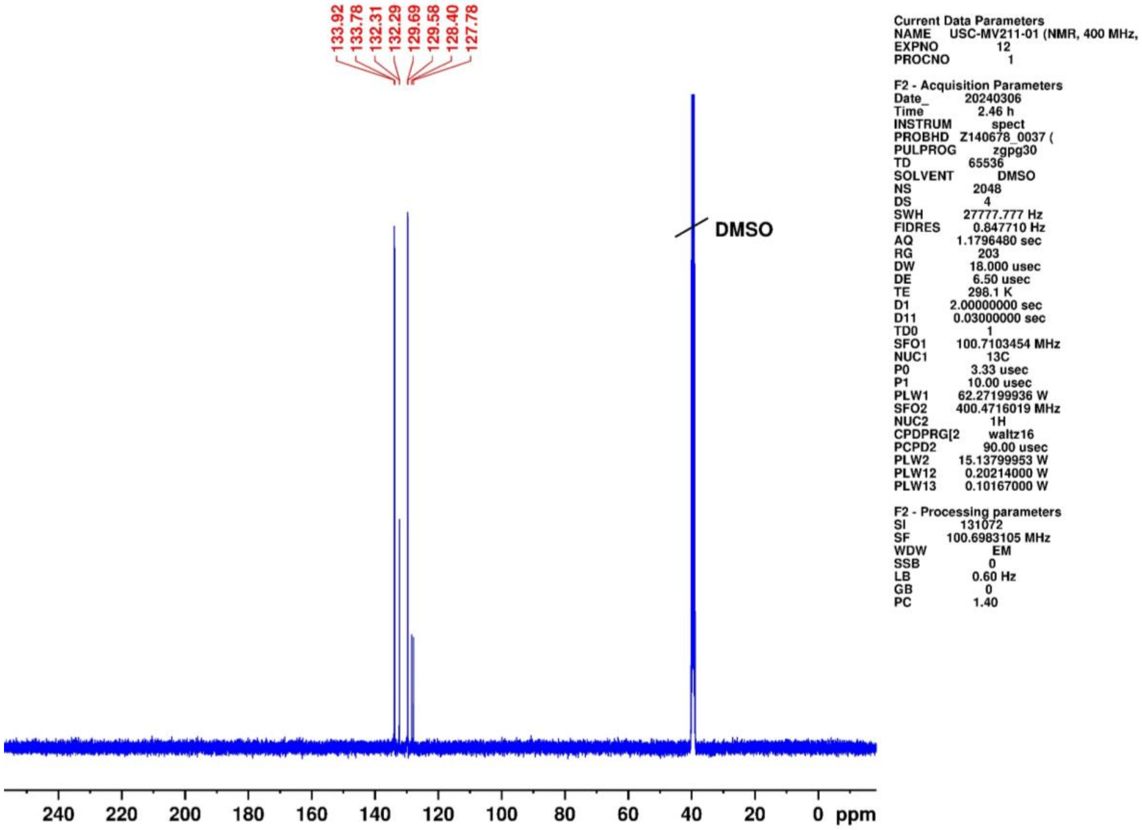

Figure S7: 100.71 MHz ^{13}C NMR spectrum of **6** in DMSO-d_6.

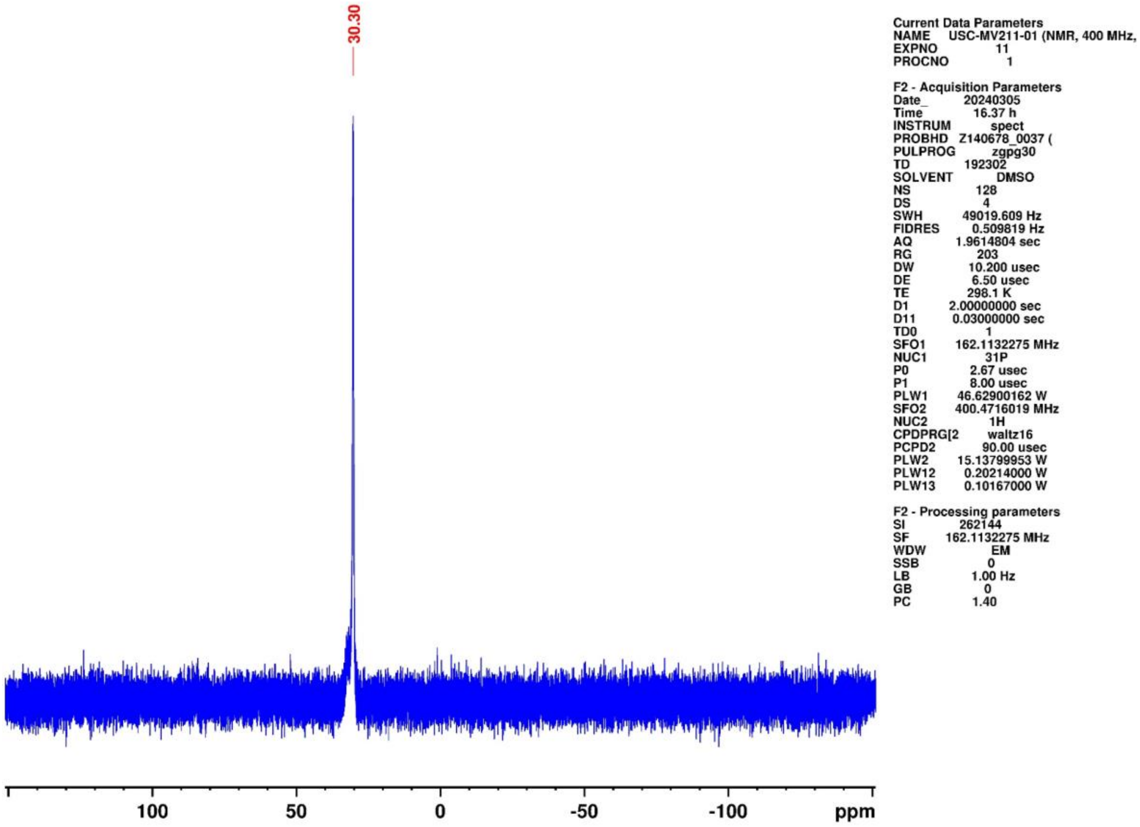

Figure S8: 162.11 MHz ^{31}P NMR spectrum of **6** in DMSO-d_6.

Synthesis of [Au(triazolato^{C6H5,COCH_3})(PPh_3)] 11

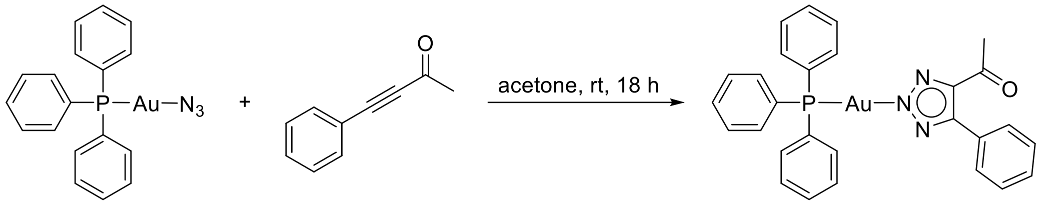

$C_{18}H_{15}AuN_3P$ 501.28 g/mol | $C_{10}H_8O$ 144.17 g/mol | $C_{28}H_{23}AuN_3OP$ 645.45 g/mol

[Au(N_3)(PPh_3)] (60.0 mg, 0.12 mmol) was dissolved in acetone (20 mL), 4-phenyl-3-butyn-2-one (19 μL, 0.13 mmol) added, and the mixture stirred at room temperature for 16 h. Then, the solvent was removed under reduced pressure to approx. 2 mL. The remaining solution was added dropwise to *n*-pentane (50 mL), the resulting precipitate filtered off, and washed with *n*-pentane (2 × 10 mL) to obtain the product as an off-white solid. Yield: 58% (45.1 mg, 0.07 mmol). **IR** (ATR): $\tilde{\nu}$ = 3072 (w), 1666 (s), 1471 (m), 1435 (s), 1100 (s), 996 (m), 955 (m), 747 (s) cm^{-1}; **^{1}H NMR** (400.47 MHz, DMSO-d_6): δ = 7.81–7.41 (m, 20H , C_6H_5), 2.57 (s, 3H, CH_3) ppm; **^{13}C NMR** (100.71 MHz, DMSO-d_6): δ = 133.88 (d, $^2J_{C2/C6,P}$ = 13.6 Hz, PPh_3-C2/C6), 132.43 (d, $^4J_{C4,P}$ = 2.3 Hz, PPh_3-C4), 129.69 (d, $^3J_{C3/C5,P}$ = 12.0 Hz, PPh_3-C3/C5), 129.34 (triazolato-phenyl-C4), 129.34 (triazolato-phenyl-C2/C6), 128.50 (triazolato-phenyl-C4), 128.11 (triazolato-phenyl-C1), 127.96 (triazolato-phenyl-C3/C5), 127.67 (d, $^1J_{C1,P}$ = 55.0 Hz, PPh3-C1), 28.20 (s, CH_3) ppm; **^{31}P NMR** (162.11 MHz, DMSO-d_6): δ = 30.56 ppm; **Elemental analysis**(%) calcd. for $C_{28}H_{23}AuN_3OP$: C 52.10, H 3.59, N 6.51; found: C 51.35, H 3.43, N 6.46.

The weak signals of the *C*O and triazolato-C4/C5 carbon atoms were not observed in the ^{13}C NMR spectrum.

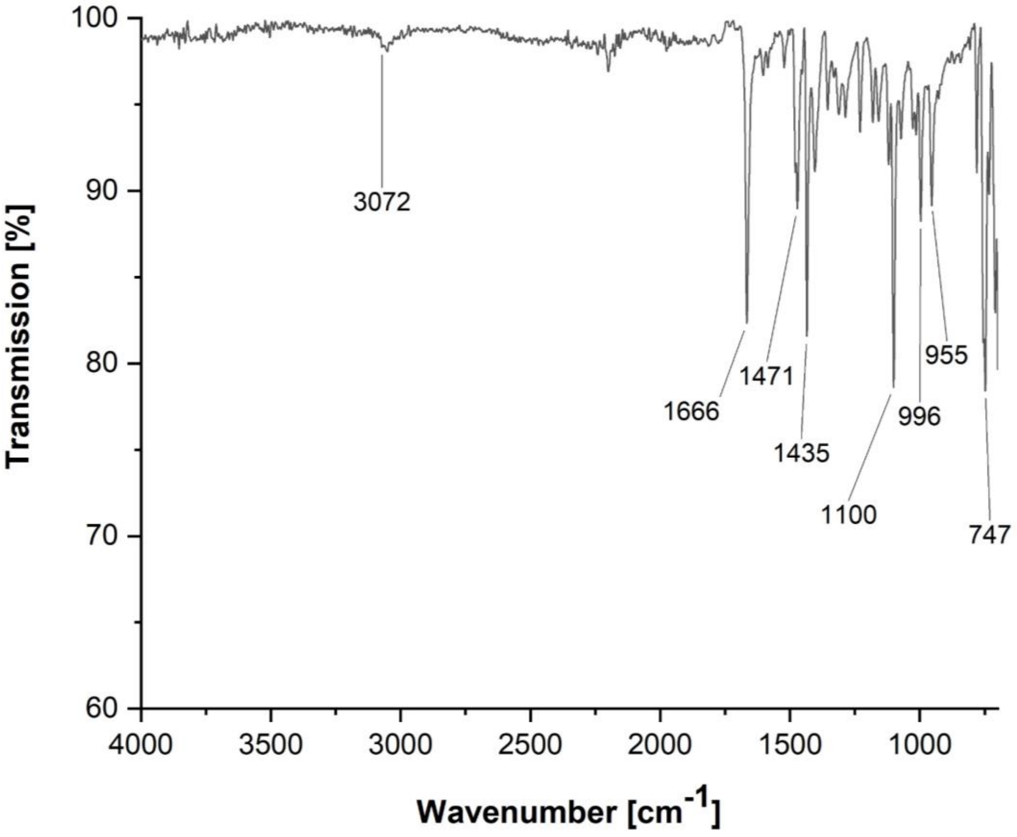

Figure S9: ATR IR spectrum of **11**.

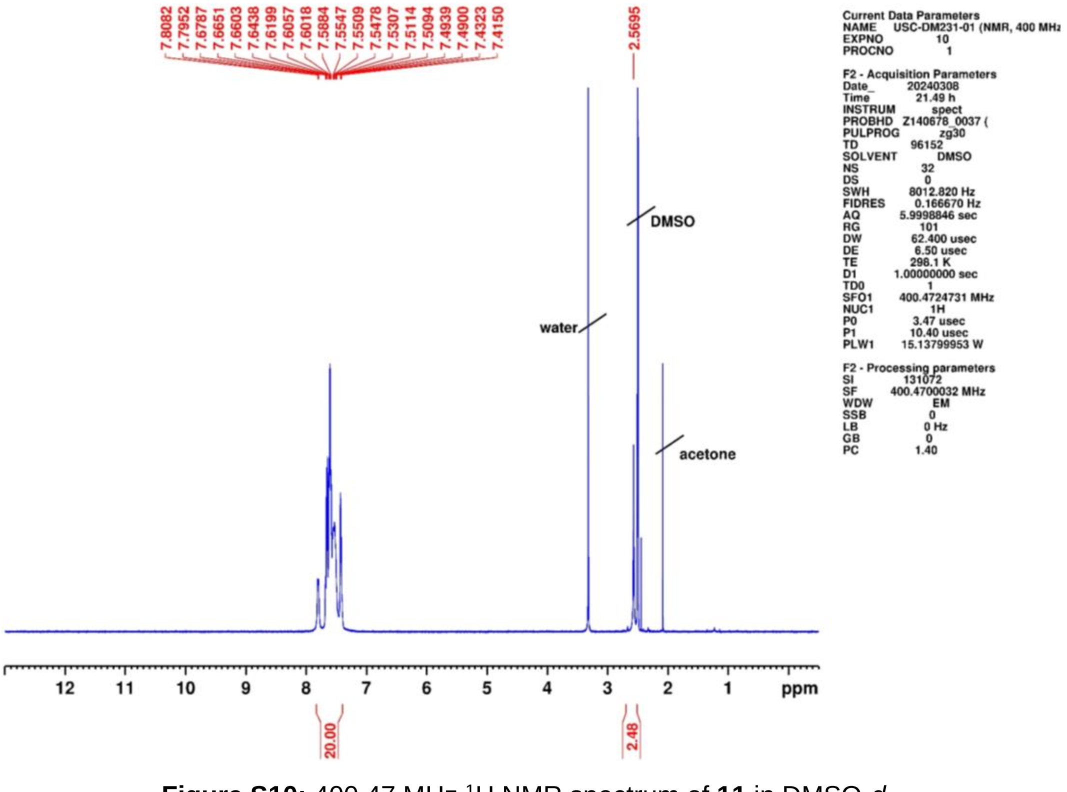

Figure S10: 400.47 MHz ^{1}H NMR spectrum of **11** in DMSO-d_6.

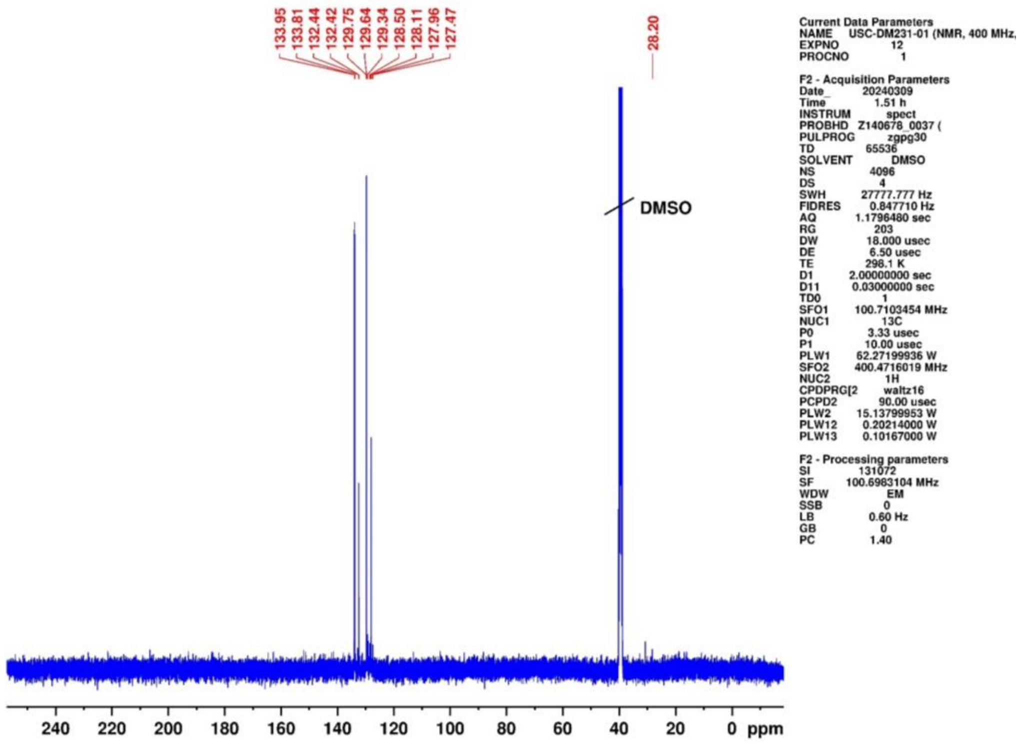

Figure S11: 100.71 MHz ^{13}C NMR spectrum of **11** in DMSO-d_6.

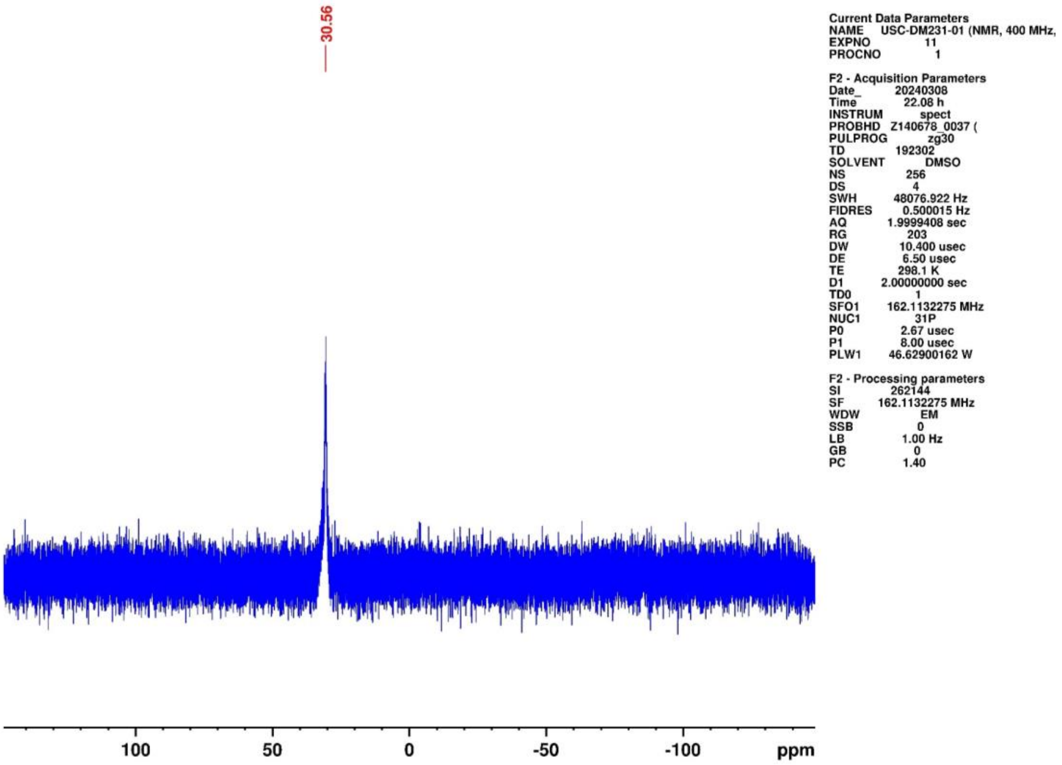

Figure S12: 162.11 MHz ^{31}P NMR spectrum of **11** in DMSO-d_6.

Synthesis of [Au(triazolato^{C6H5,biotin})(PPh$_3$)] 12

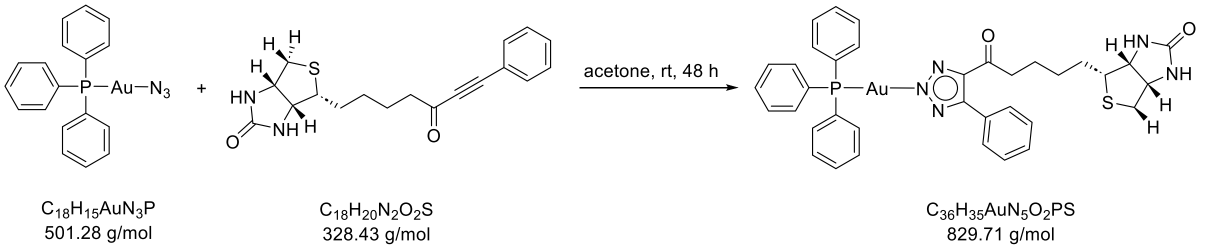

[Au(N$_3$)(PPh$_3$)] (60.0 mg, 0.12 mmol) was dissolved in acetone (20 mL), 7-[(3aS,4S,6aR)-2-oxohexahydro-1H-thieno-[3,4-d]imidazol-4-yl]-1-phenyl-1-heptyn-3-one (42.7 mg, 0.13 mmol) added, and the mixture stirred at room temperature for 48 h. Then, the solvent was removed under reduced pressure to approx. 2 mL and the mixture added dropwise to *n*-pentane (50 mL). The crude material filtered off and recrystallized from ethyl acetate (10 mL) to obtain the product as an off-white solid. Yield: 62% (66.7 mg, 0.08 mmol). **IR** (ATR): $\tilde{\nu}$ = 2917 (w), 1702 (s), 1671 (s), 1436 (m), 1264 (w), 1101 (m), 745 (s) cm^{-1}; **^{1}H NMR** (400.47 MHz, DMSO-d_6): δ = 7.80–7.41 (m, 20H, C$_6$*H*$_5$), 6.44 (s, 1H, biotin-N*H*), 6.34 (s, 1H , biotin-N*H*), 4.31–4.27 (m, 1H, biotin-H6a), 4.15–4.11 (m, 1H, biotin-H3a), 3.13–3.03 (m, 3H, biotin-H4/sidechain-H7), 2.81 (dd, 1H, $^2J_{H6,H6'}$ = 12.5 Hz, $^3J_{H6,H6a}$ = 5.1 Hz, biotin-H6), 2.57 (d, 1H, $^2J_{H6',H6}$ = 12.5 Hz, biotin-H6'), 1.70–1.30 (m, 6H, biotin-H4/H5/H6) ppm; **^{13}C NMR** (100.71 MHz, DMSO-d_6): δ = 162.69 (NH*C*OCH), 133.91 (d, $^2J_{C2/C6,P}$ = 13.6 Hz, PPh$_3$-C2/C6), 132.46 (d, $^4J_{C4,P}$ = 1.8 Hz, PPh$_3$-C4), 129.70 (d, $^3J_{C3/C5,P}$ = 11.8 Hz, PPh$_3$-C3/C5), 129.35 (triazolato-phenyl-C2/C6), 128.52 (triazolate-phenyl-C4), 127.96 (triazolato-phenyl-C3/C5), 127.65 (d, $^1J_{C1,P}$ = 63.0 Hz, PPh$_3$-C1), 61.04 (biotin-C3a), 59.17 (biotin-C6a), 55.44 (biotin-C4), 40.19 (sidechain-C4), 39.85 (biotin-C6), 28.31 (sidechain-C5), 28.18 (sidechain-C7), 23.98 (sidechain-C6) ppm; **^{31}P NMR** (162.11 MHz, DMSO-d_6): δ = 30.52 ppm; **MS** (ESI$^+$, CH$_3$OH): *m*/*z* = 852.1785 [M+H]$^+$, **Elemental analysis**(%) calcd. for C$_{36}$H$_{35}$AuN$_5$O$_2$PS: C 52.11, H 4.25, N 8.44, S 3.86; found: C 52.06, H 4.46, N 8.19, S 4.01.

The weak signals of the *C*O, triazolato-phenyl-C1, and triazolato-C4/C5 carbon atoms were not observed in the ^{13}C NMR spectrum.

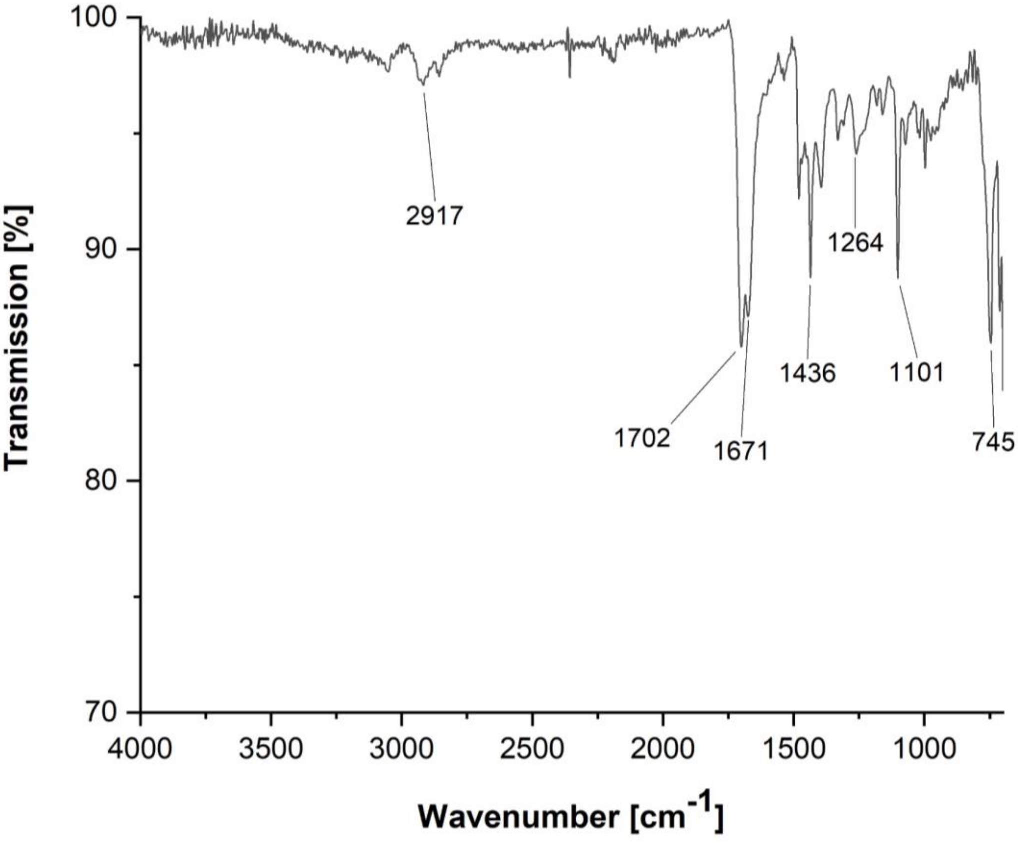

Figure S13: ATR IR spectrum of **12**.

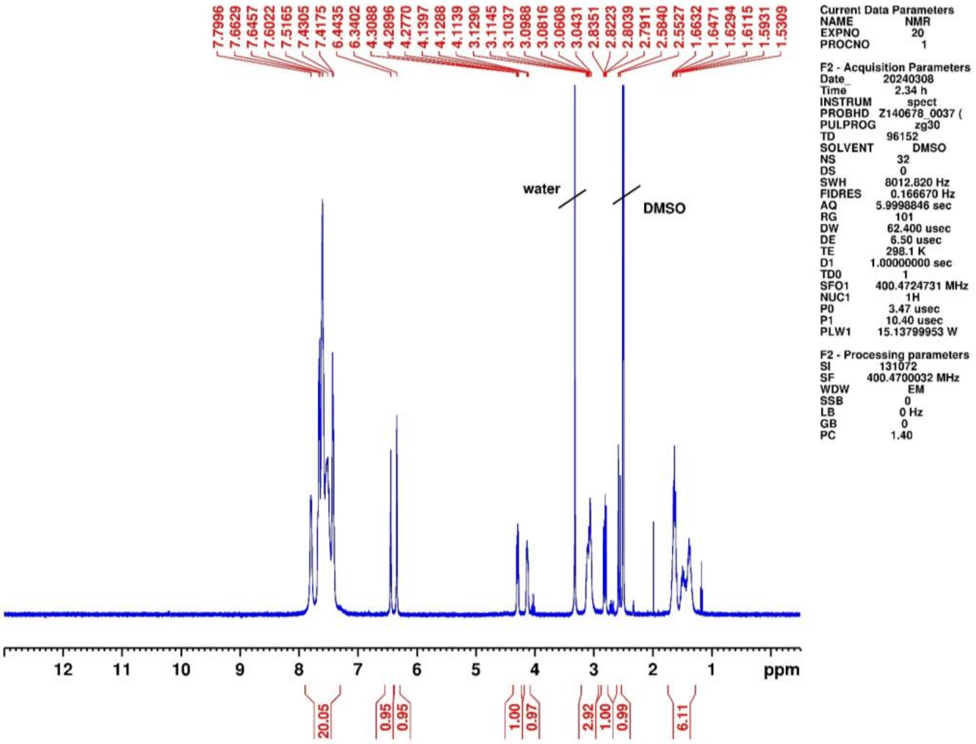

Figure S14: 400.47 MHz ^{1}H NMR spectrum of **12** in DMSO-d_6.

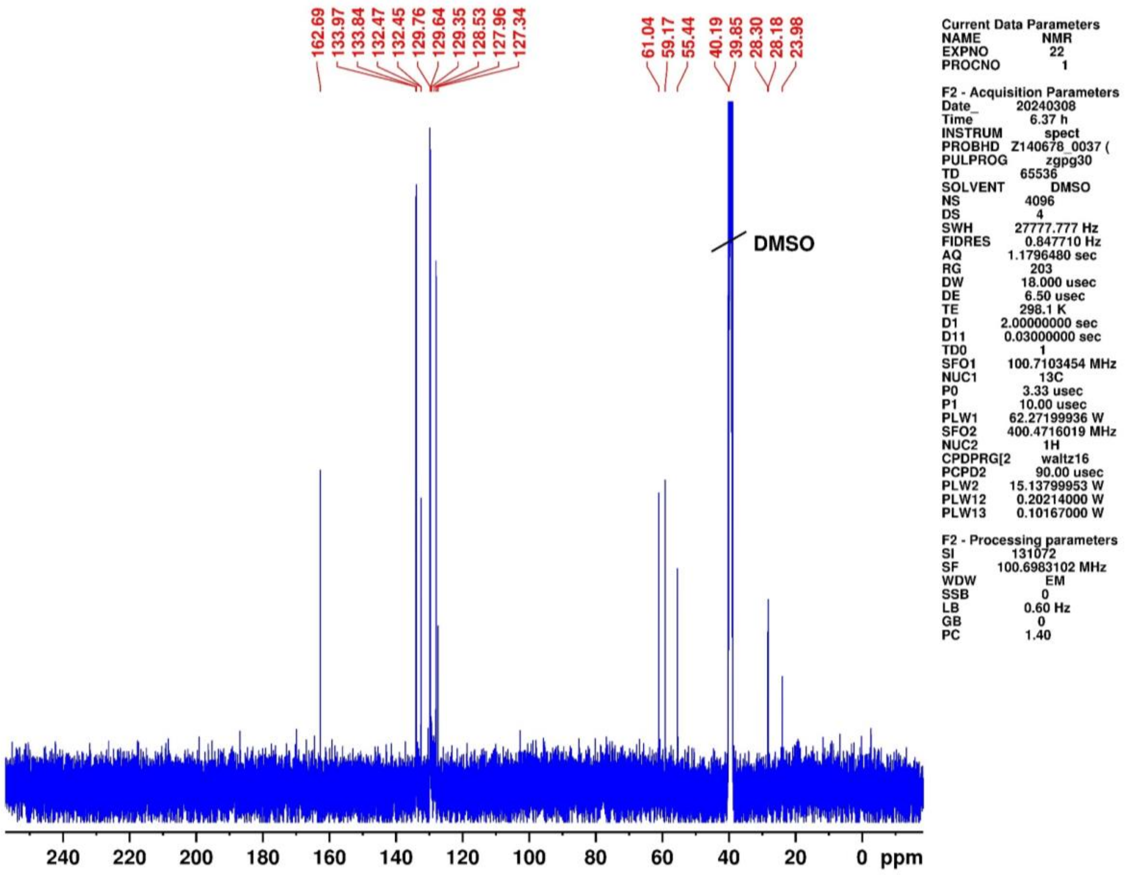

Figure S15: 100.71 MHz ^{13}C NMR spectrum of **12** in DMSO-d_6.

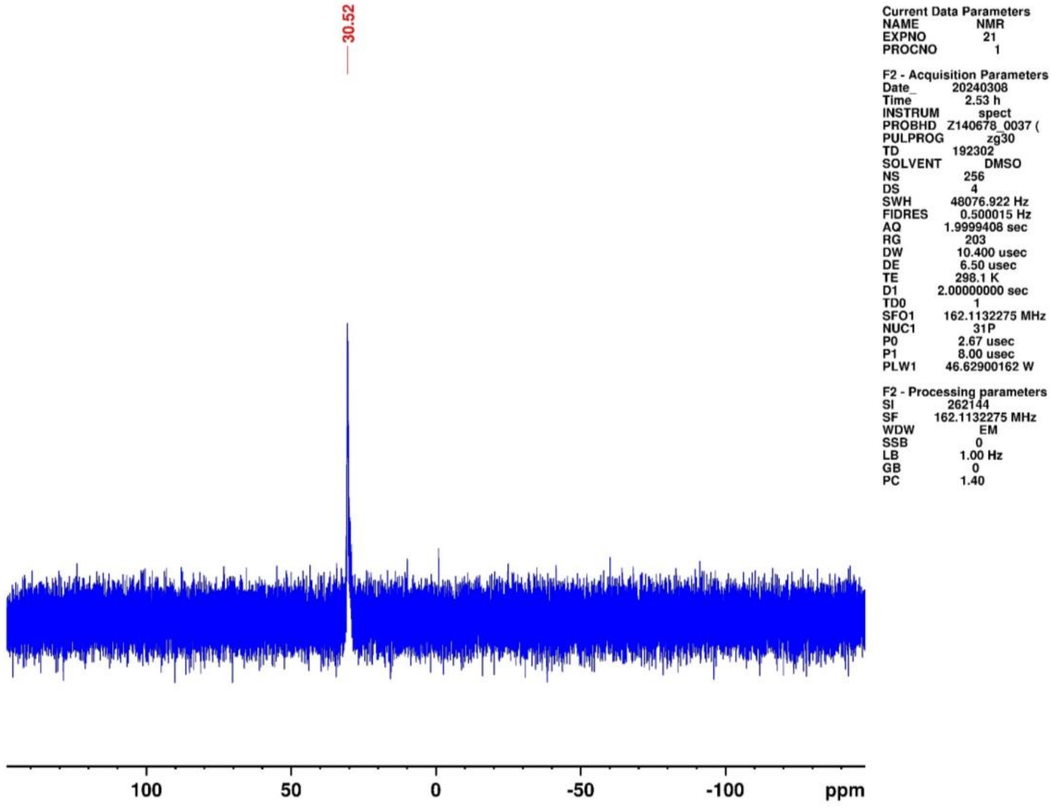

Figure S16: 162.11 MHz ^{31}P NMR spectrum of **12** in DMSO-d_6.

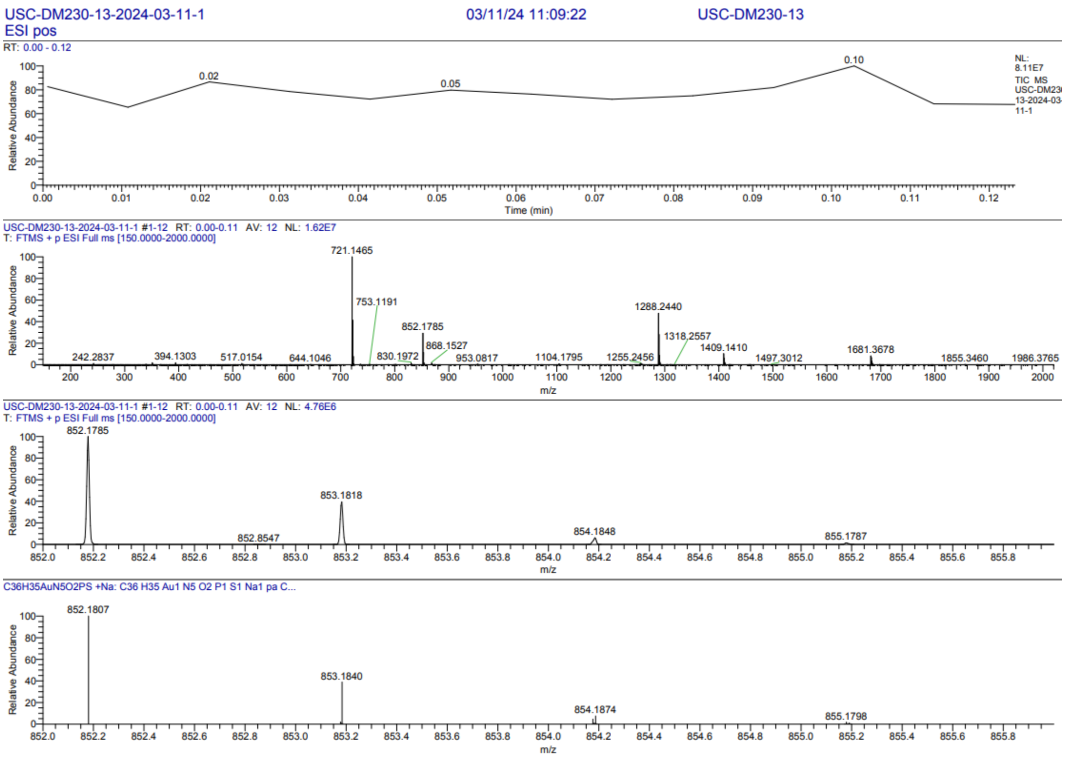

Figure S17: ESI positive mode mass spectrum of **12** in methanol.

Synthesis of [Pt(triazolato^{C6H5,biotin})(dpb)] 14

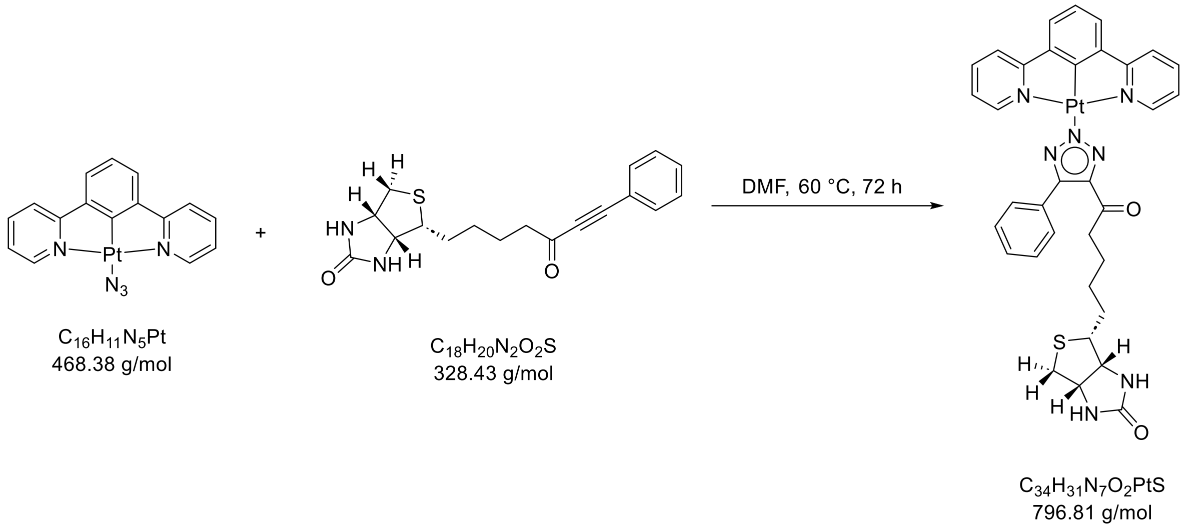

[Pt(N$_3$)(dpb)] (60.0 mg, 0.13 mmol) was dissolved in *N*,*N*-dimethylformamide (10 mL), followed by addition of 7-[(3aS,4S,6aR)-2-oxohexahydro-1H-thieno-[3,4-d]imidazol-4-yl]-1-phenyl-1-heptyn-3-one (128.1 mg, 0.39 mmol). The mixture was stirred at 60 °C for 72 h. Then, the solvent was removed under reduced pressure and acetone (10 mL) added. The mixture was added dropwise to diethyl ether (50 mL). The resulting precipitate was filtered off, washed with diethyl ether (2 × 20 mL), and dried under vacuum to obtain the product as an orange solid. Yield: 75% (76.2 mg, 0.10 mmol). **IR** (ATR): $\tilde{\nu}$ = 3068 (w), 2930 (w), 1700 (s), 1611 (m), 1453 (m), 1399 (m), 1159 (w), 760 (s) cm^{-1}; **^{1}H NMR** (400.47 MHz, DMSO-*d*$_6$): δ = 9.45 (d, 2H, $^3J_{H6',H5'/H6'',H5''}$ = 5.7 Hz, dpb-H6'/H6''), 8.24–8.16 (m, 4H, dpb-H3'/H3'' and dpb-H4'/H4''), 8.05 (d, 2H, $^3J_{H2,H3/H6,H5}$ = 7.3 Hz, phenyl-H2/H6), 7.79 (d, 2H, $^3J_{H3/H5,H4}$ = 7.7 Hz, dpb-H3/H5), 7.56 (t, 2H, $^3J_{H5',H4'/H6'//H5'',H4''/H6''}$ = 6.9 Hz, dpb-H5'/H5''), 7.46 (t, 2H, $^3J_{H3,H2/H4//H5,H4/H6}$ = 7.6 Hz, phenyl-H3/H5), 7.38 (t, 1H, $^3J_{H4,H3/H5}$ = 7.4 Hz, phenyl-H4), 7.31 (t, 1H, $^3J_{H4,H3/H5}$ = 7.7 Hz, dpb-H4), 6.46 (s, 1H, biotin-N*H*), 6.36 (s, 1H, biotin-N*H*'), 4.31−4.28 (m, 1H, biotin-H6a), 4.17−4.13 (m, 1H, biotin-H3a), 3.25−3.16 (m, 3H, biotin-H4/sidechain-H7), 2.82 (dd, 1H, $^2J_{H6,H6'}$ = 12.3 Hz, $^3J_{H6,H6a}$ = 5.1 Hz, biotin-H6), 2.58 (d, 1H, $^2J_{H6',H6}$ = 12.5 Hz, biotin-H6'), 1.80−1.41 (m, 6H, biotin-H4/H5/H6) ppm; **^{13}C NMR** (100.71 MHz, DMSO-*d*$_6$): δ = 195.65 (sidechain-CH$_2$*C*O), 167.23 (dpb-C2'/C2''), 162.71 (NH*C*ONH), 161.84 (dpb-C1), 151.98 (dpb-C6'/C6''), 147.19 (triazolato-C5), 142.51 (dpb-C2/C6), 141.82 (triazolato-C4), 140.73 (dpb-C4'/C4''), 132.37 (phenyl-C1), 128.69 (phenyl-C2/C6), 127.89 (phenyl-C3/C5), 127.76 (pheny-C4), 124.57 (dpb-C4), 124.10 (dpb-C5'/C5''), 123.91 (dpb-C3/C5), 120.56 (dpb-C3'/C3''), 61.09 (biotin-C3a), 59.17 (biotin-C6a), 55.48 (biotin-C4), 40.08 (sidechain-C4), 39.86 (biotin-C6), 28.46

(sidechain-C5), 28.25 (sidechain-C7), 24.25 (sidechain-C6) ppm; **^{195}Pt NMR** (85.77 MHz, DMSO-d_6): δ = -3686 ppm; **MS** (ESI$^+$, CH_3OH): m/z = 819.1788 $[M+H]^+$ **Elemental analysis**(%) calcd. for $C_{34}H_{31}N_7O_2PtS(H_2O)$: C 50.12, H 4.08, N 12.03, S 3.94; found: C 50.15, H 3.87, N 11.90, S 4.07.

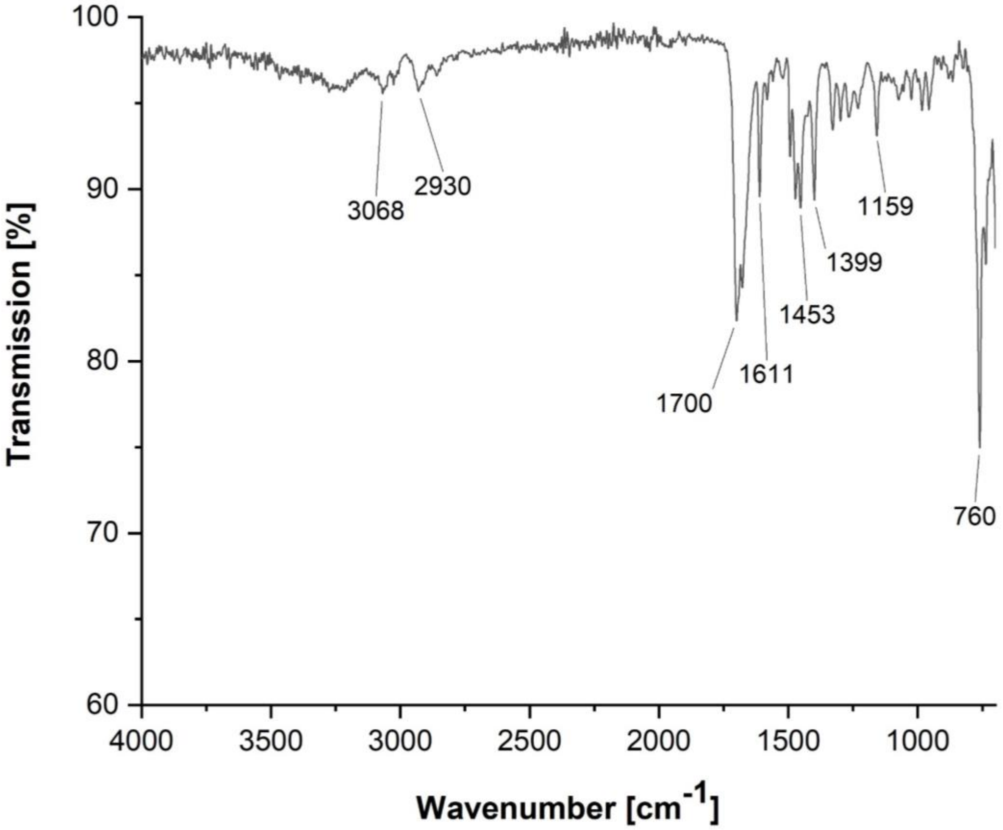

Figure S18: ATR IR spectrum of **14**.

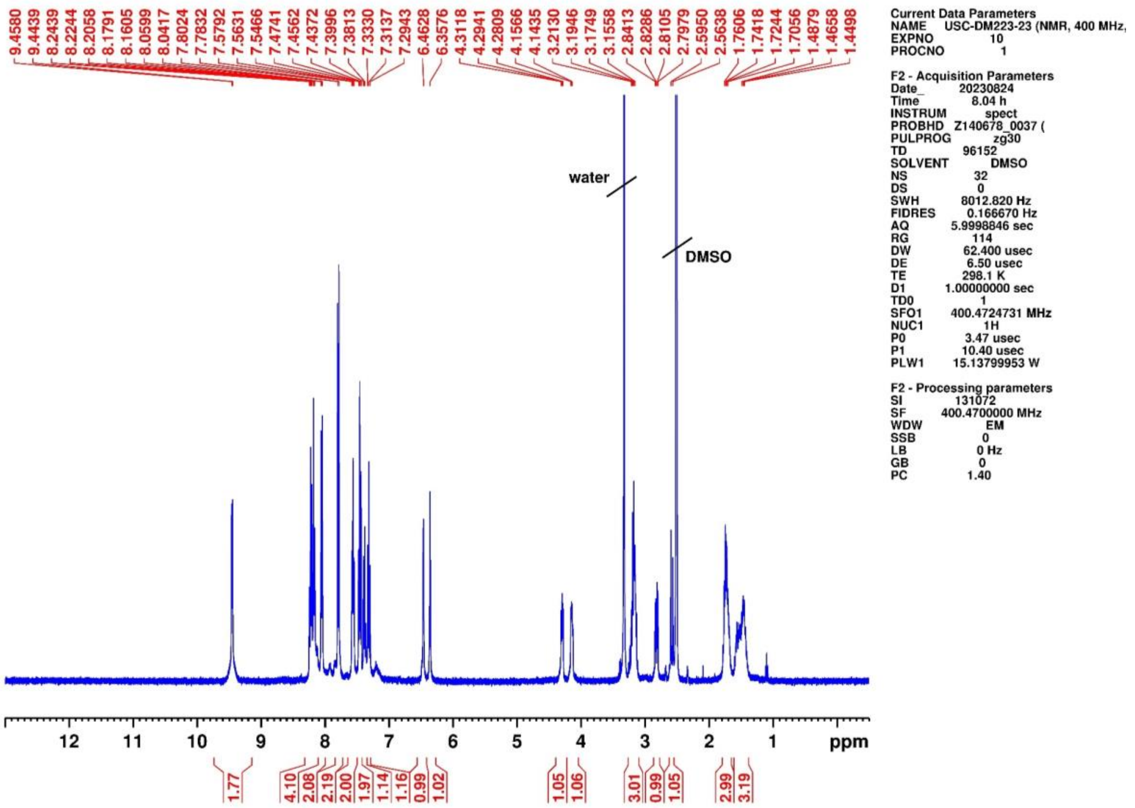

Figure S19: 400.47 MHz ^{1}H NMR spectrum of **14** in DMSO-d_6.

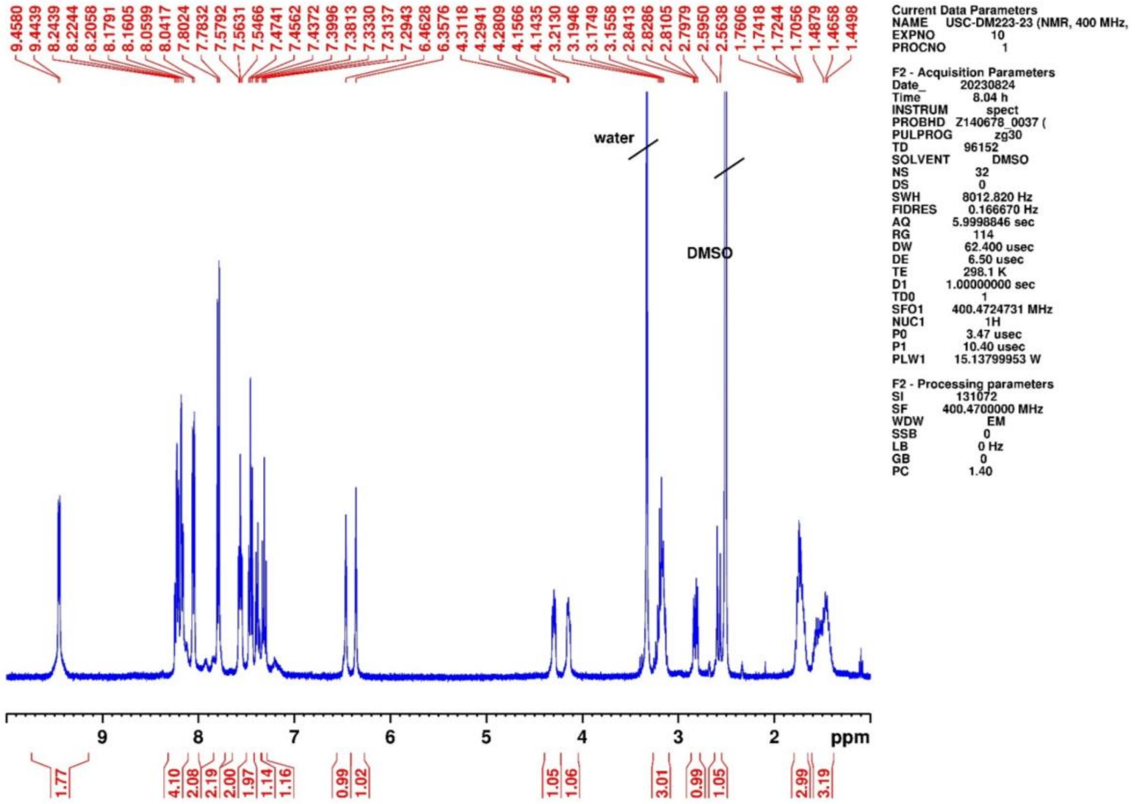

Figure S20: 400.47 MHz ^{1}H NMR spectrum of **14** in DMSO-d_6.

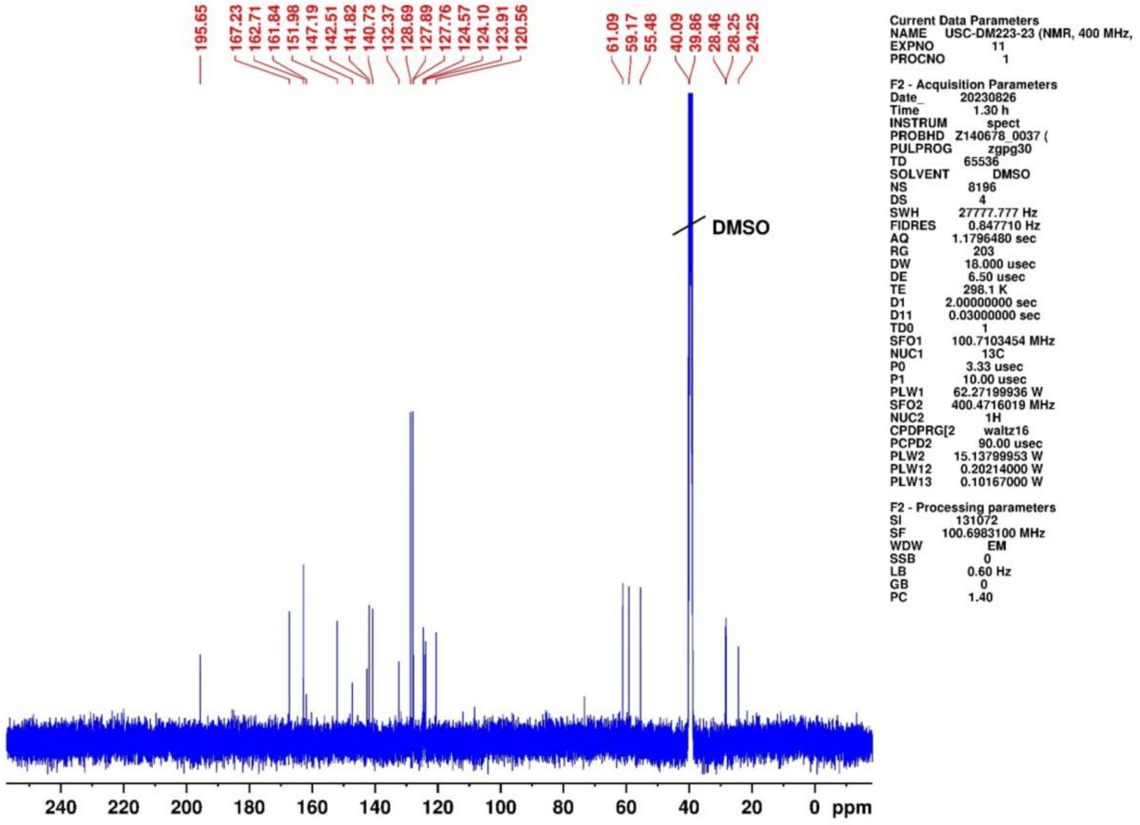

Figure S21: 100.71 MHz ^{13}C NMR spectrum of **14** in DMSO-d_6.

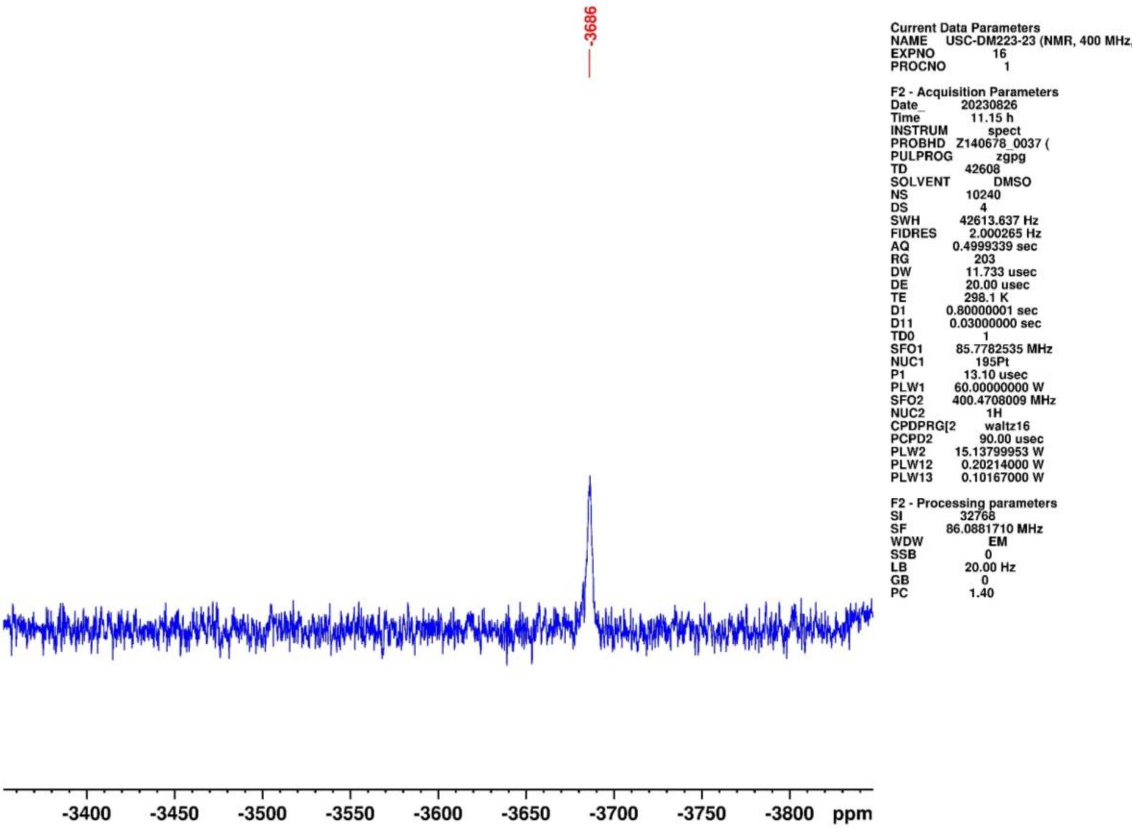

Figure S22: 85.76 MHz ^{195}Pt NMR spectrum of **14** in DMSO-d_6.

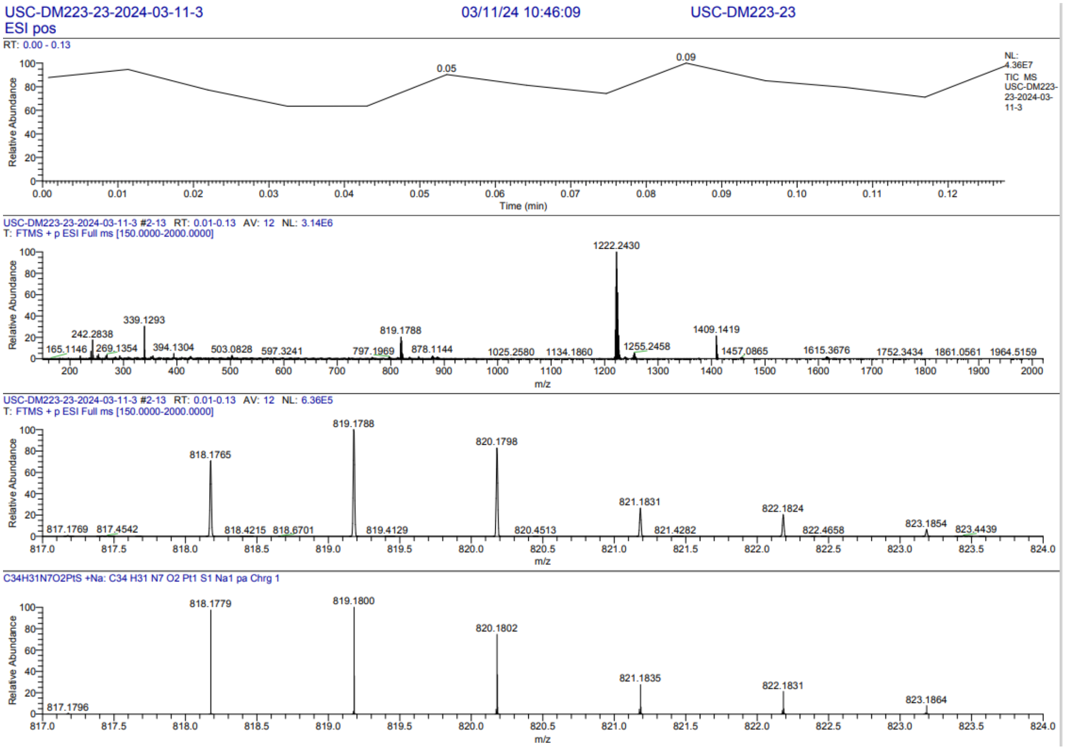

Figure S23: ESI positive mode mass spectrum of **14** in methanol.

Synthesis of [Pt(triazolato^{C6H5,biotin})(terpy)]PF_6 16

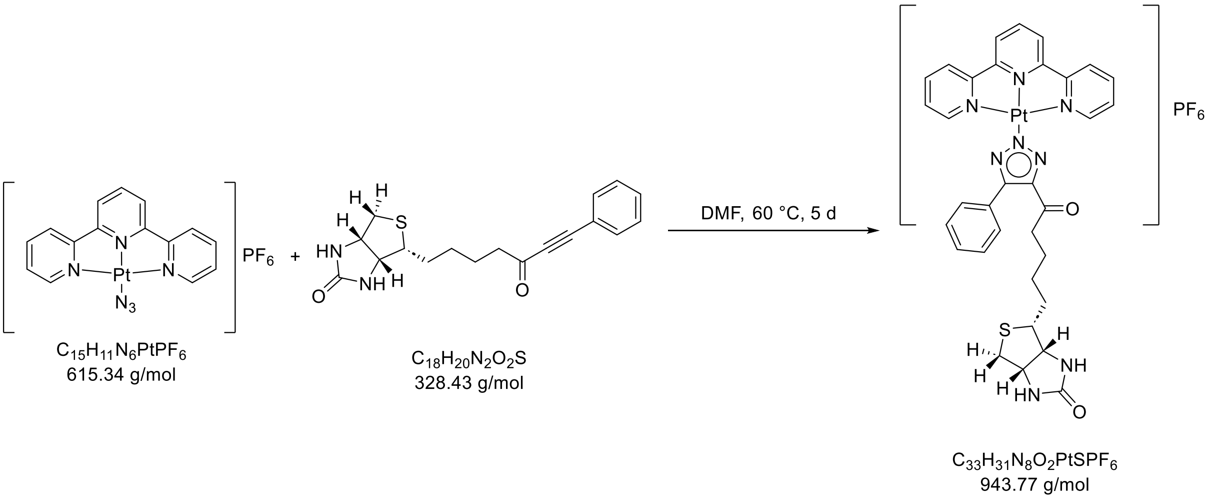

[Pt(N_3)(terpy)]PF_6 (60.0 mg, 0.10 mmol) was dissolved in *N*,*N*-dimethylformamide (10 mL) followed by addition of 7-[(3aS,4S,6aR)-2-oxohexahydro-1H-thieno-[3,4-d]imidazol-4-yl]-1-phenyl-1-heptyn-3-one (94.9 mg, 0.29 mmol). The mixture was stirred at 60 °C for 6 d. Then, the solvent was removed under reduced pressure and acetone (10 mL) added. The mixture was added dropwise to diethyl ether (50 mL). The resulting precipitate was filtered off, washed with diethyl ether (2 × 20 mL), and dried under vacuum to obtain the product as an orange solid. Yield: 51% (50.1 mg, 0.05 mmol). **IR** (ATR): $\tilde{\nu}$ = 3041 (w), 2945 (w), 1710 (s), 1680 (s), 1461 (s), 1253 (m), 1170 (w), 839 (vs), 767 (s) cm^{-1}; **^{1}H NMR** (400.47 MHz, DMSO-d_6): δ = 9.13 (d, 2H, $^3J_{H6/H6'',H5/H5''}$ = 5.9 Hz, terpy-H6/H6”), 8.65–8.57 (m, 5H, terpy-H3/H3’’ and terpy-H3’/H4’/H5’), 8.50 (dt, 2H, $^3J_{H4/H4'',H5/H5''}$ = 7.9 Hz, $^3J_{H4/H4'',H6/H6''}$ = 1.3 Hz, terpy-H4/H4”), 7.98–7.94 (m, 4H, terpy-H5/H5’’, phenyl-H2/H6), 7.51–7.41 (m, 3H, phenyl-H3/H4/H5), 6.44 (s, 1H, biotin-N*H*), 6.38 (s, 1H , biotin-N*H’*), 4.33−4.29 (m, 1H, biotin-H6a), 4.16−4.13 (m, 1H, biotin-H3a), 3.16−3.04 (m, 3H, biotin-H4/sidechain-H7), 2.82 (dd, 1H, $^2J_{H6,H6'}$ = 12.4 Hz, $^3J_{H6,H6a}$ = 5.1 Hz, biotin-H6), 2.57 (d, 1H, $^2J_{H6',H6}$ = 12.5 Hz, biotin-H6’), 1.73−1.32 (m, 6H, biotin-H4/H5/H6) ppm; **^{13}C NMR** (100.71 MHz, DMSO-d_6): δ = 194.87 (sidechain-CH_2*C*O), 162.74 (NH*C*OCH), 157.92 (terpy-C2/C2”), 154.46 (terpy-C2’/C6’), 152.01 (terpy-C6/C6”), 147.98 (triazolate-C5), 143.39 (triazolate-C4), 142.99 (terpy-C4’), 142.90 (terpy-C4/C4”), 130.94 (phenyl-C1), 128.86 (terpy-C5/C5”), 128.81 (phenyl-C2/C6), 128.49 (phenyl-C4), 128.05 (terpy-phenyl-C3/C5), 125.84 (terpy-C3/C3”), 124.17 (terpy-C3’/C5’), 61.12 (biotin-C3a), 59.22 (biotin-C6a), 55.49 (biotin-C4), 40.19 (sidechain-C4), 39.86 (biotin-C6), 28.34 (sidechain-C5), 28.36 (sidechain-C7), 23.71 (sidechain-C6) ppm; **^{195}Pt NMR** (85.77 MHz, DMSO-d_6):

δ = -2699 ppm; **MS** (ESI$^+$, CH_3OH): *m/z* = 798.1918 [M+H]$^+$ **Elemental analysis**(%) calcd. for $C_{33}H_{31}F_6N_8O_2PPtS$: C 42.00, H 3.31, N 11.87, S 3.40; found: C 42.26, H 3.32, N 11.56, S 3.39.

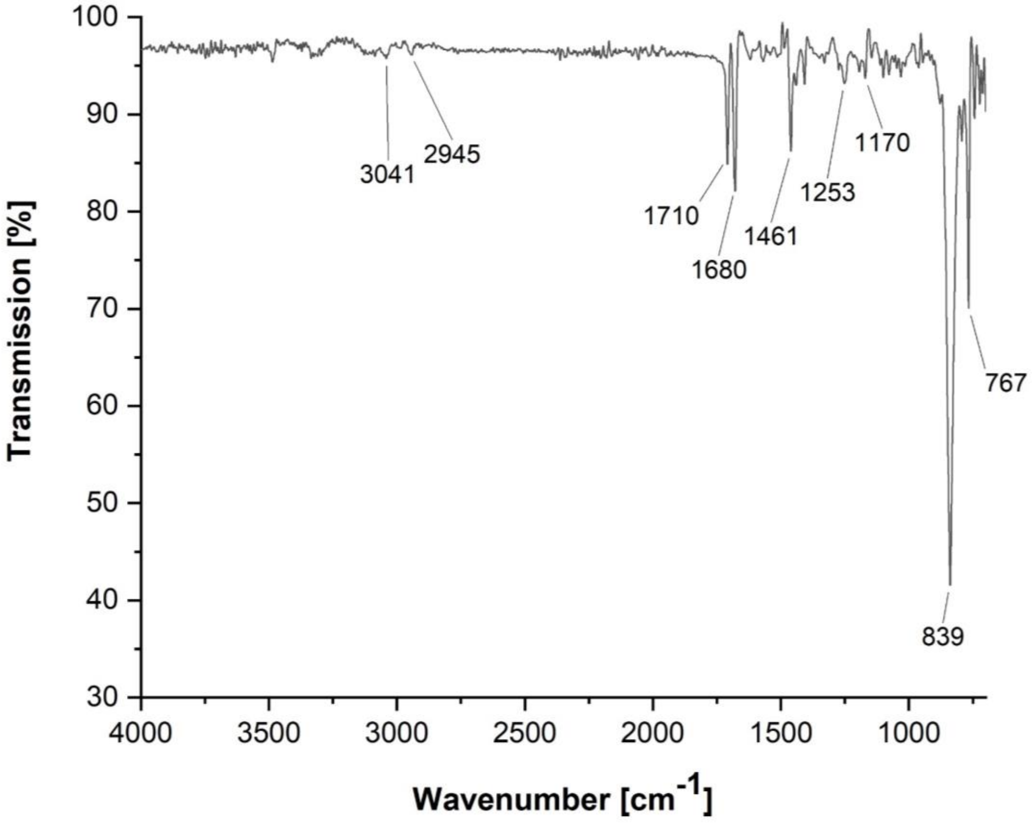

Figure S24: ATR IR spectrum of **16**.

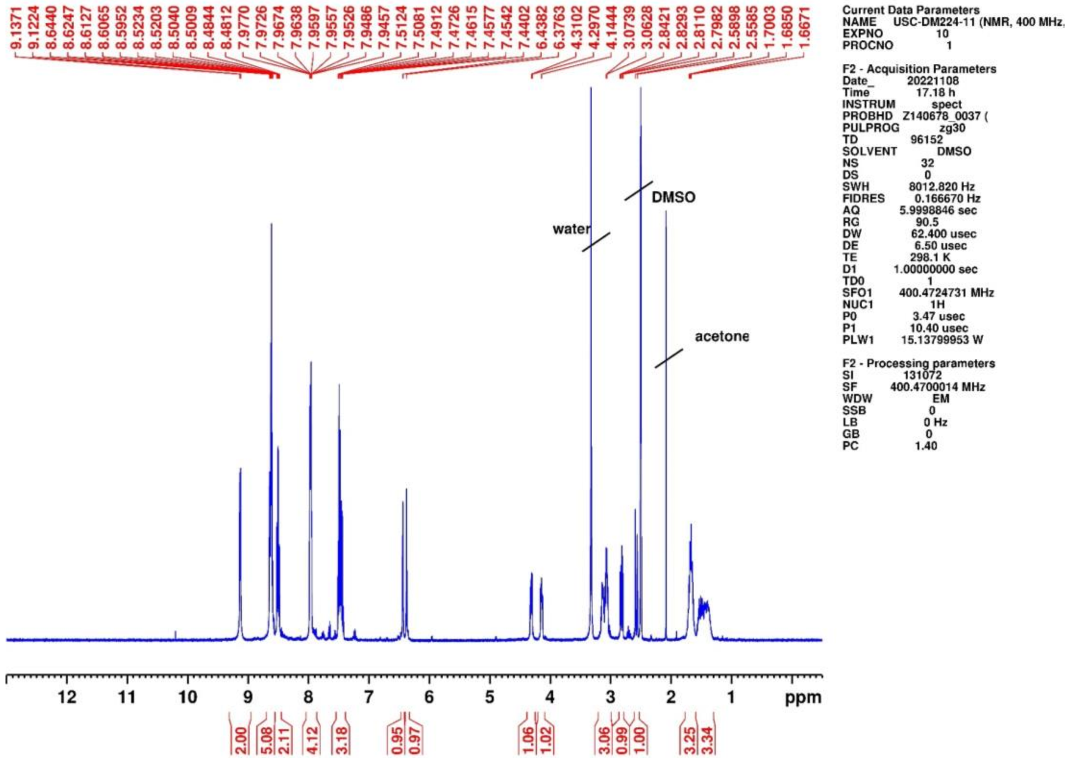

Figure S25: 400.47 MHz ^{1}H NMR spectrum of **16** in DMSO-d_6.

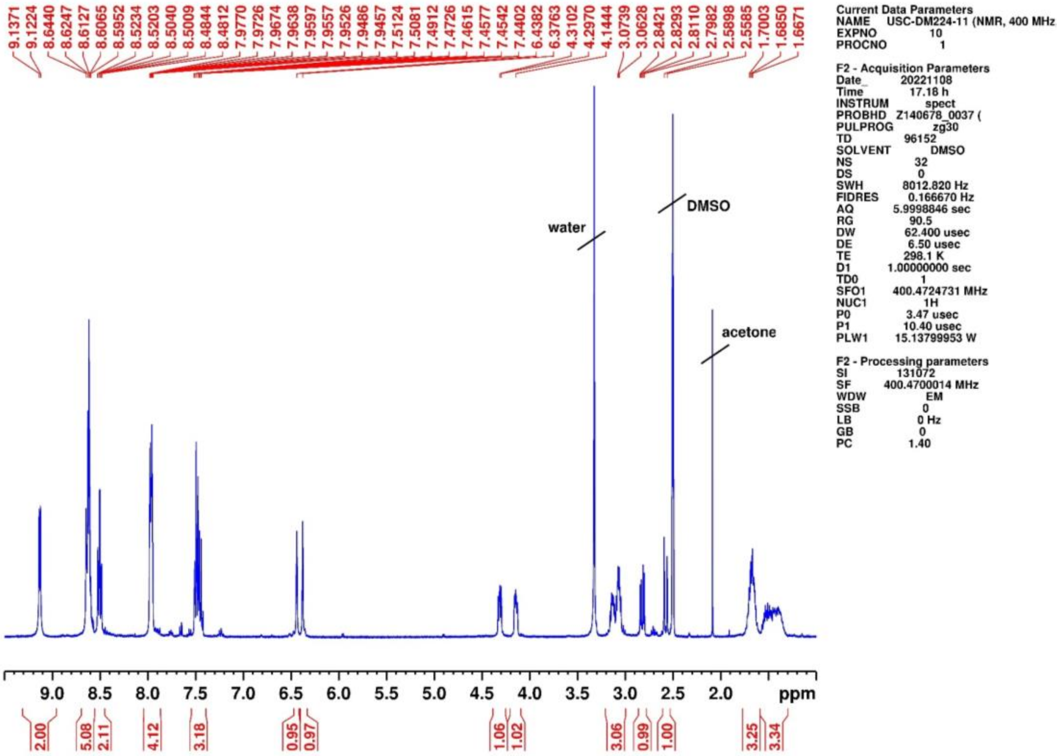

Figure S26: Enlarged view of the ^{1}H NMR spectrum of **16** in DMSO-d_6

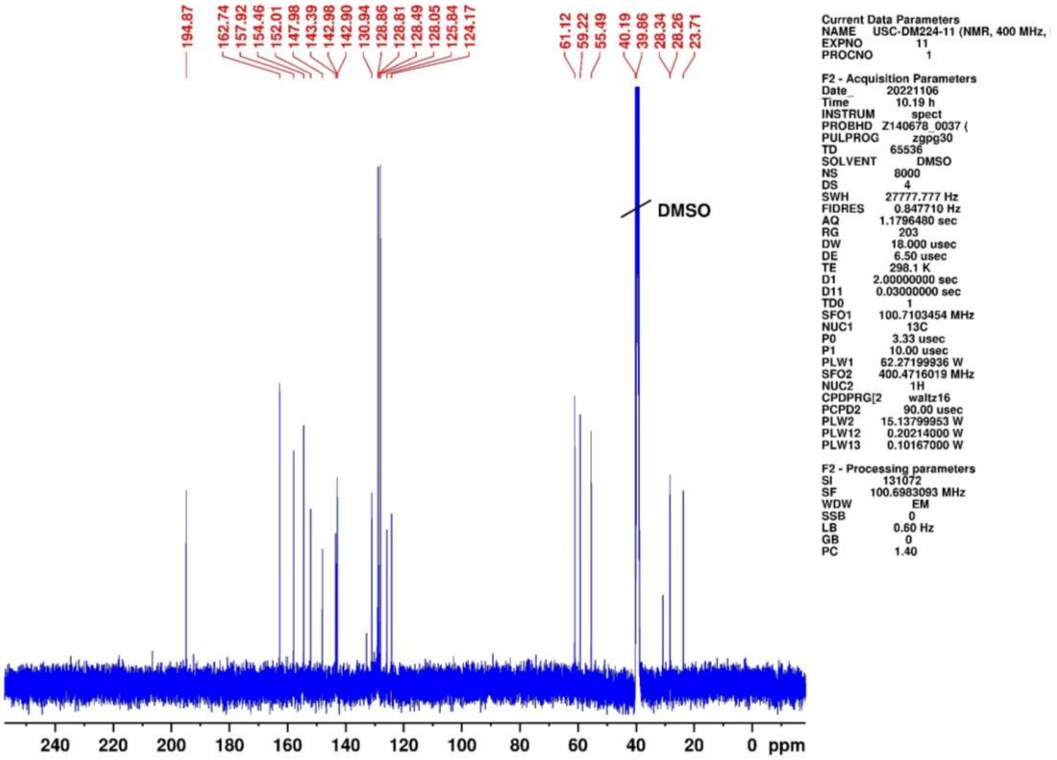

Figure S27: 100.71 MHz ^{13}C NMR spectrum of **16** in DMSO-d_6.

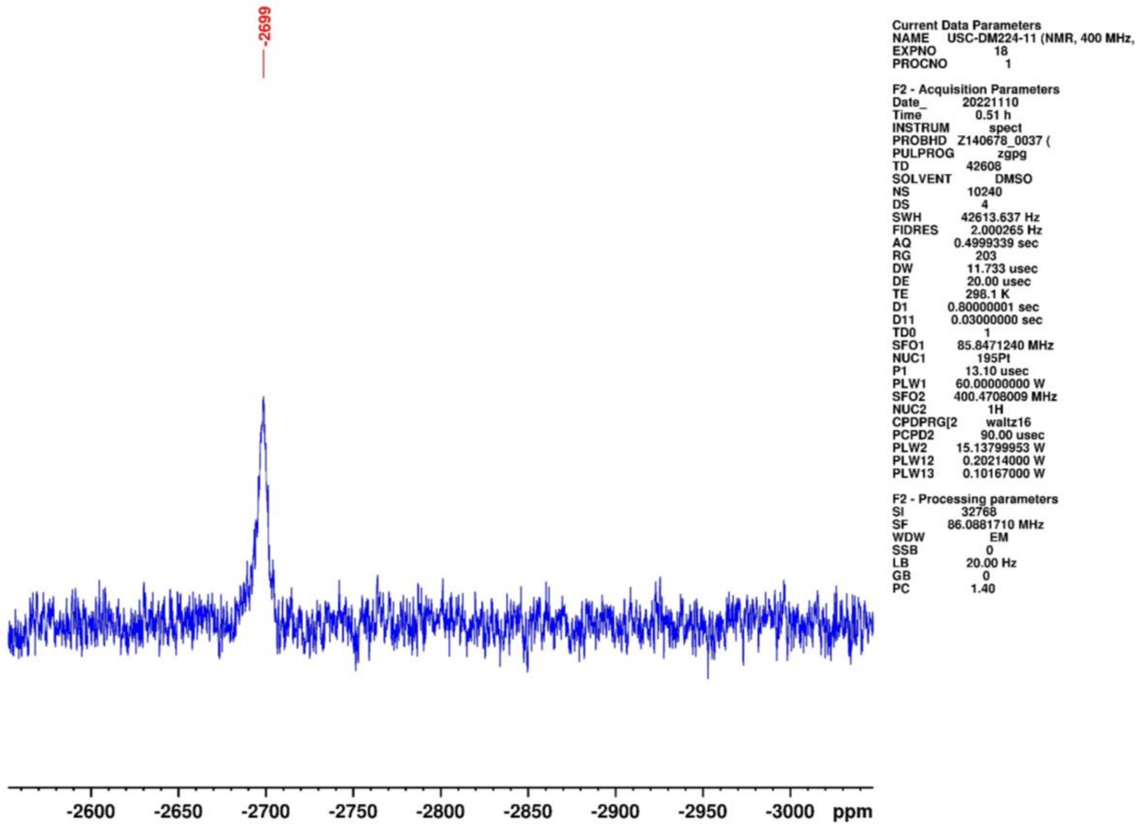

Figure S28: 85.76 MHz ^{195}Pt NMR spectrum of **16** in DMSO-d_6.

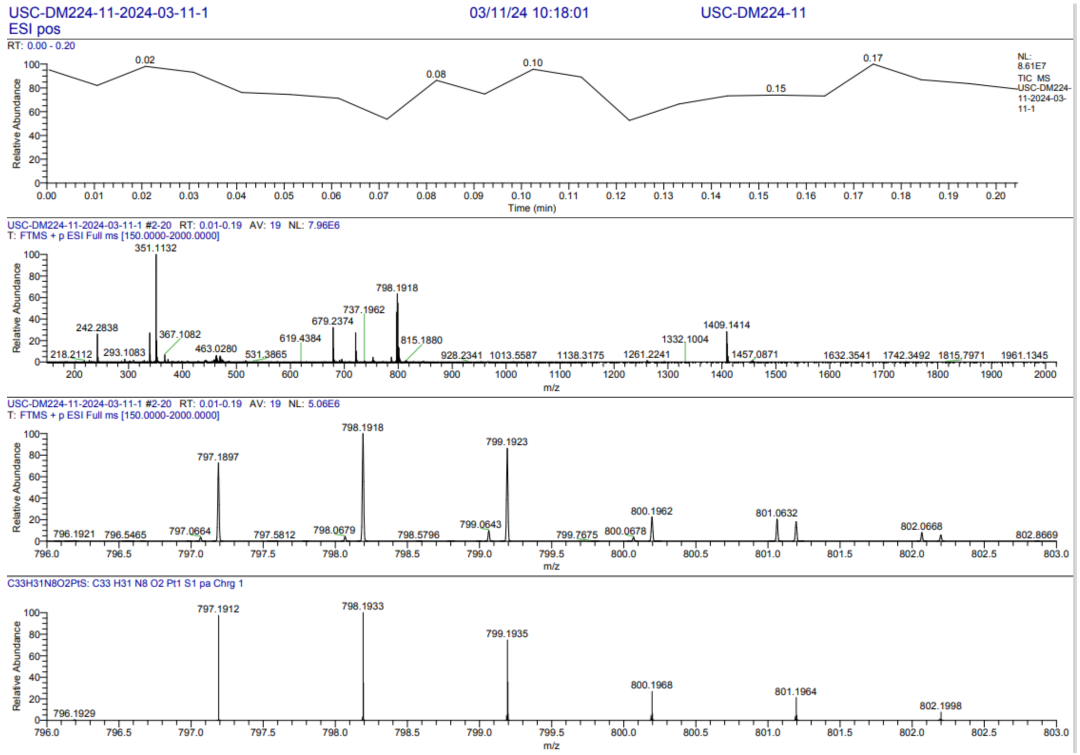

Figure S29: ESI positive mode mass spectrum of **16** in methanol.

Synthesis of [Ir(triazolato^{C6H5,biotin})(ppy)(terpy]PF_6 18

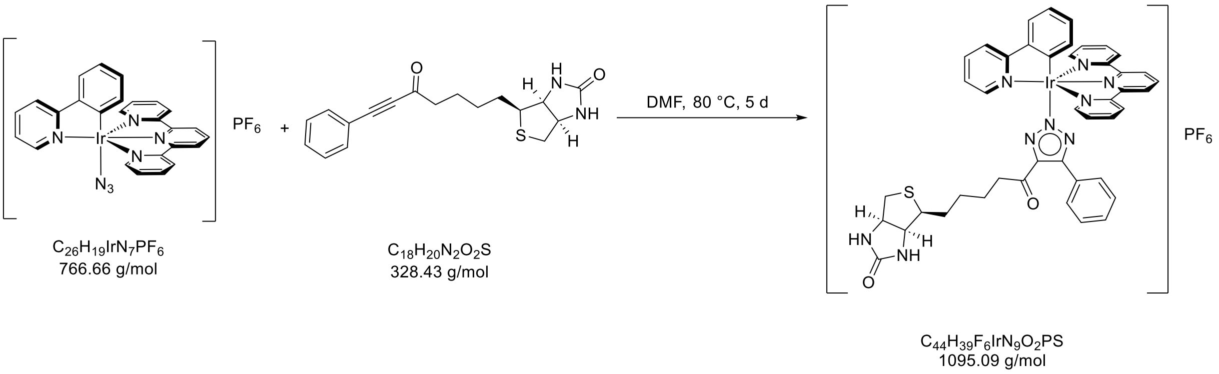

[Ir(N_3)(terpy)(ppy)]PF_6 (60.0 mg, 0.08 mmol) was dissolved in *N*,*N*-dimethylformamide (10 mL) followed by addition of 7-[(3aS,4S,6aR)-2-oxohexahydro-1H-thieno-[3,4-d]imidazol-4-yl]-1-phenyl-1-heptyn-3-one (38.6 mg, 0.12 mmol). The mixture was stirred at 80 °C for 5 d. Then, the solvent was removed under reduced pressure and acetone (10 mL) added. The mixture was added dropwise to diethyl ether (50 mL). The resulting precipitate was filtered off, washed with diethyl ether (2 × 20 mL), and dried under vacuum. The crude mixture was recrystallized from ethyl acetate (10 mL) to obtain the product as a yellow solid. Yield: 63% (49.5 mg, 0.05 mmol). **IR** (ATR): $\tilde{\nu}$ = 1698 (m), 1440 (m), 1251 (w), 835 (vs), 769 (s) cm^{-1}; **^{1}H NMR** (500.13 MHz, DMSO-d_6): δ = 9.26 (d, 1H, $^3J_{H6,H5}$ = 6.0 Hz, ppy-H6), 8.92 (d, 2H, $^3J_{H3'/H5',H4'}$ = 8.3 Hz, terpy-H3'/H5'), 8.82–8.73 (m, 2H, terpy-H3/H3''), 8.59–8.55 (m, 2H, terpy-H4'/ppy-H3), 8.31 (t, 1H, $^3J_{H4,H3/H5}$ = 7.8 Hz, ppy-H4), 8.26 (t, 2H, $^3J_{H4,H3/H5//H4',H3''/H5''}$ = 7.8 Hz, terpy-H4/H4''), 8.02 (d, 1H, $^3J_{H3',H4'}$ = 7.8 Hz, ppy-H3'), 7.88 (d, 2H, $^3J_{H6,H5/H6'',H5''}$ = 5.5 Hz, terpy-H6/H6''), 7.79 (t, 1H, $^3J_{H5,H4/H6}$ = 6.1 Hz, ppy-H5), 7.65–7.60 (m, 4H, terpy-H5/H5'' and phenyl-H2/H6), 7.28–7.21 (m, 3H, phenyl-H3/H4/H5), 7.00 (t, 1H, $^3J_{H4',H3'/H5'}$ = 7.5 Hz, ppy-H4'), 6.84 (t, 1H, $^3J_{H5',H4'/H6'}$ = 7.5 Hz, ppy-H5'), 6.48 (s, 1H, biotin-N*H*), 6.44 (s, 1H , biotin-NH), 6.12 (d, 1H, $^3J_{H6',H5'}$ = 7.5 Hz, ppy-H6'), 4.36–4.32 (m, 1H, biotin-H6a), 4.16–4.13 (m, 1H, biotin-H3a), 3.09 (dt, 1H, $^3J_{H4,sidechain-H7}$ = 9.4 Hz, $^3J_{H4,H3a}$ = 4.7 Hz, biotin-H4), 2.84 (dd, 1H, $^2J_{H6,H6'}$ = 12.7 Hz, $^3J_{H6,H6a}$ = 5.0 Hz, biotin-H6), 2.62 (d, 1H, $^2J_{H6',H6}$ = 12.3 Hz, biotin-H6'), 2.00–1.07 (m, 8H, biotin-H4/H5/H6/H7) ppm; **^{13}C NMR** (125.76 MHz, DMSO-d_6): δ = 195.04 (CH_2*C*O), 165.88 (ppy-C2), 162.84 (NH*C*ONH), 158.00 (terpy-C2/C2''), 155.39 (terpy-C6'), 155.38 (terpy-C2'), 152.16 (terpy-C6/C6''), 150.41 (ppy-C6), 147.20 (triazolato-C5), 144.40 (ppy-C2'), 143.26 (ppy-C1'), 142.43 (triazolato-C4), 140.90 (terpy-C4'), 140.63 (terpy-C4/C4''), 140.38 (ppy-C4), 131.66 (phenyl-C1), 131.16 (ppy-C6'), 130.12 (ppy-C5'), 129.09 (terpy-C5/C5''), 128.15 (phenyl-C2/C6), 127.71 (phenyl-C3/C4/C5), 125.62

(terpy-C3/C3''), 125.20 (ppy-C3'), 124.72 (ppy-C5), 124.33 (terpy-C3'/C5'), 124.07 (ppy-C4'), 121.06 (ppy-C3), 61.16 (biotin-C3a), 59.25 (biotin-C6a), 55.48 (biotin-C4), 39.75 (biotin-C6), 39.61 (siedechain-C4), 28.39 (sidechain-C5), 28.16 (sidechain-C7), 24.49 (sidechain-C6) ppm; **MS** (ESI$^+$, CH_3OH): *m/z* = 950.2550 [M-PF_6]$^+$; **Elemental analysis**(%) calcd. for $C_{44}H_{39}F_6IrN_9O_2PS$: C 48.26, H 3.59, N 11.51, S 2.93; found: C 48.71, H 4.01, N 10.71, S 3.09.

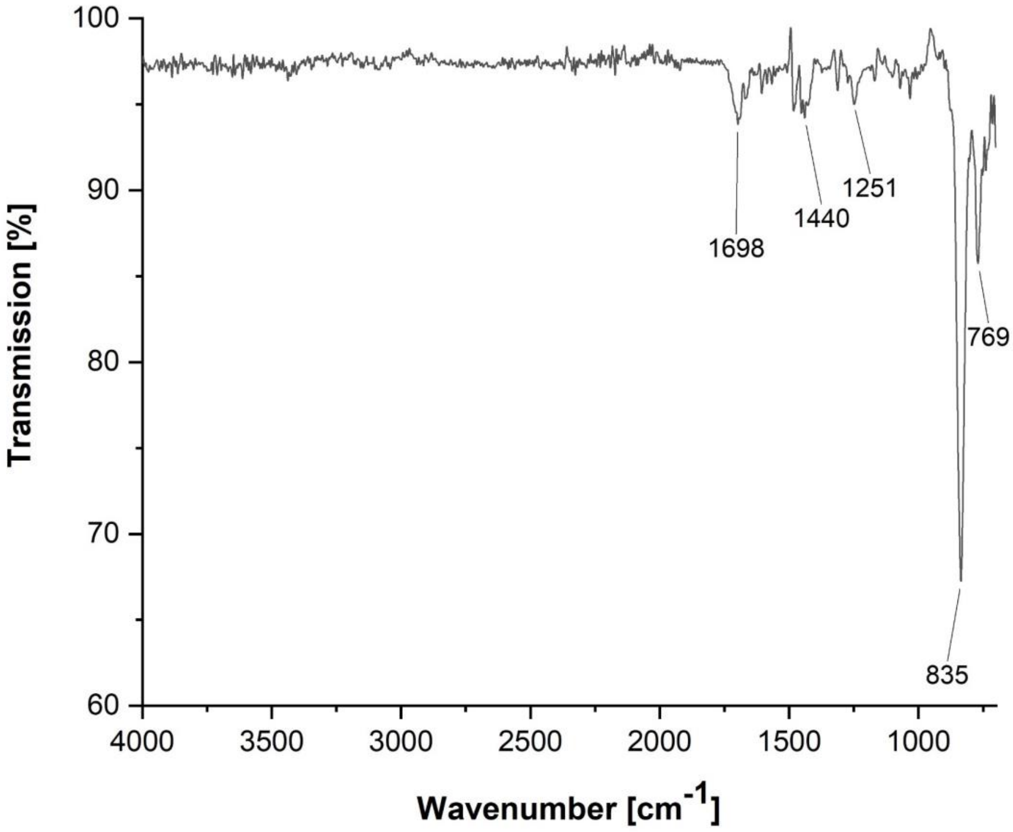

Figure S30: ATR IR spectrum of **18**.

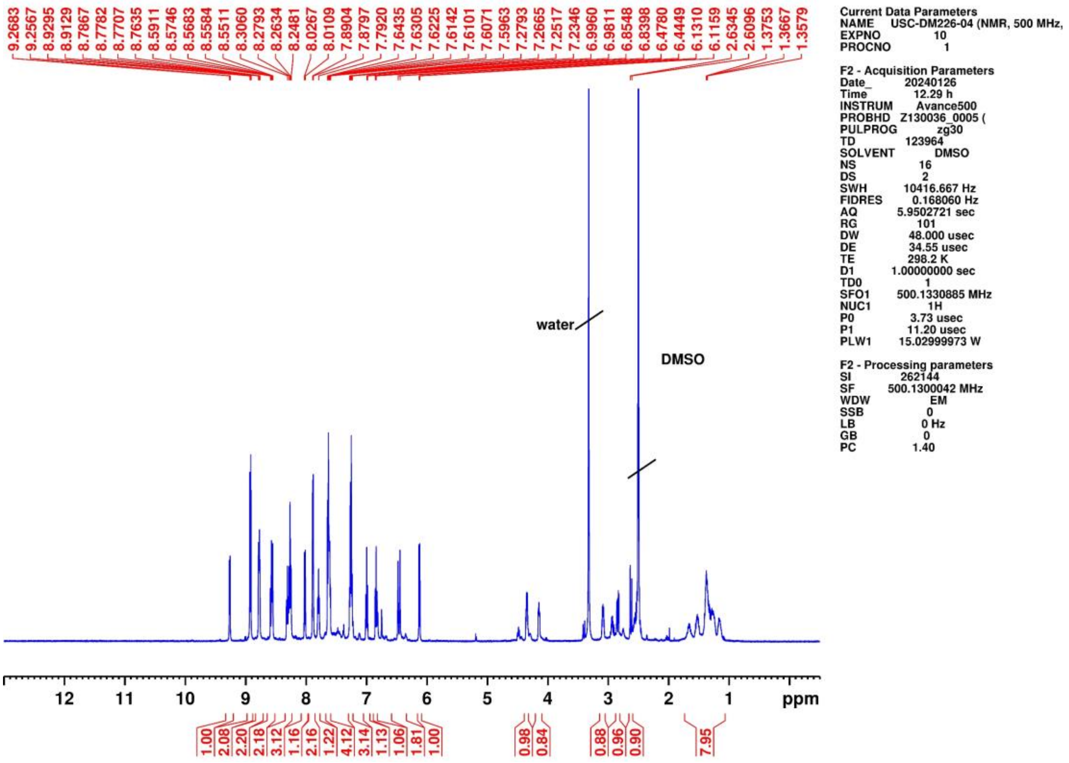

Figure S31: 500.13 MHz 1H NMR spectrum of **18** in DMSO-d_6**.**

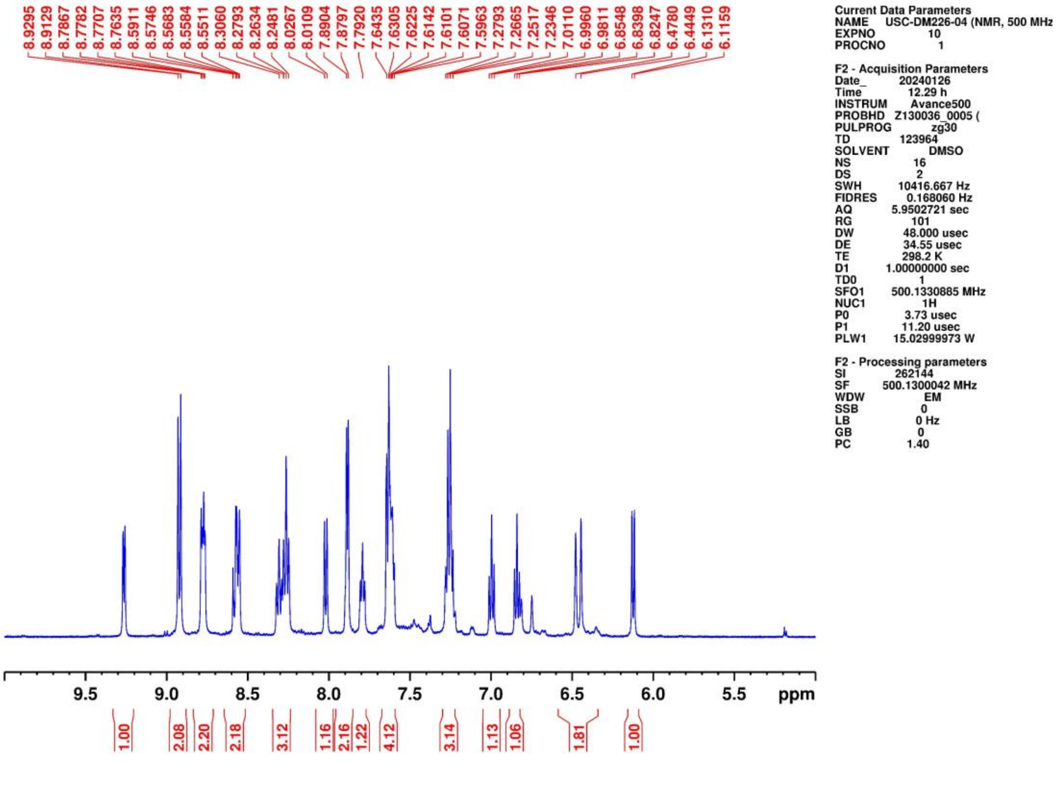

Figure S32: Enlarged view of the ^{1}H NMR spectrum of **18** in DMSO-d_6.

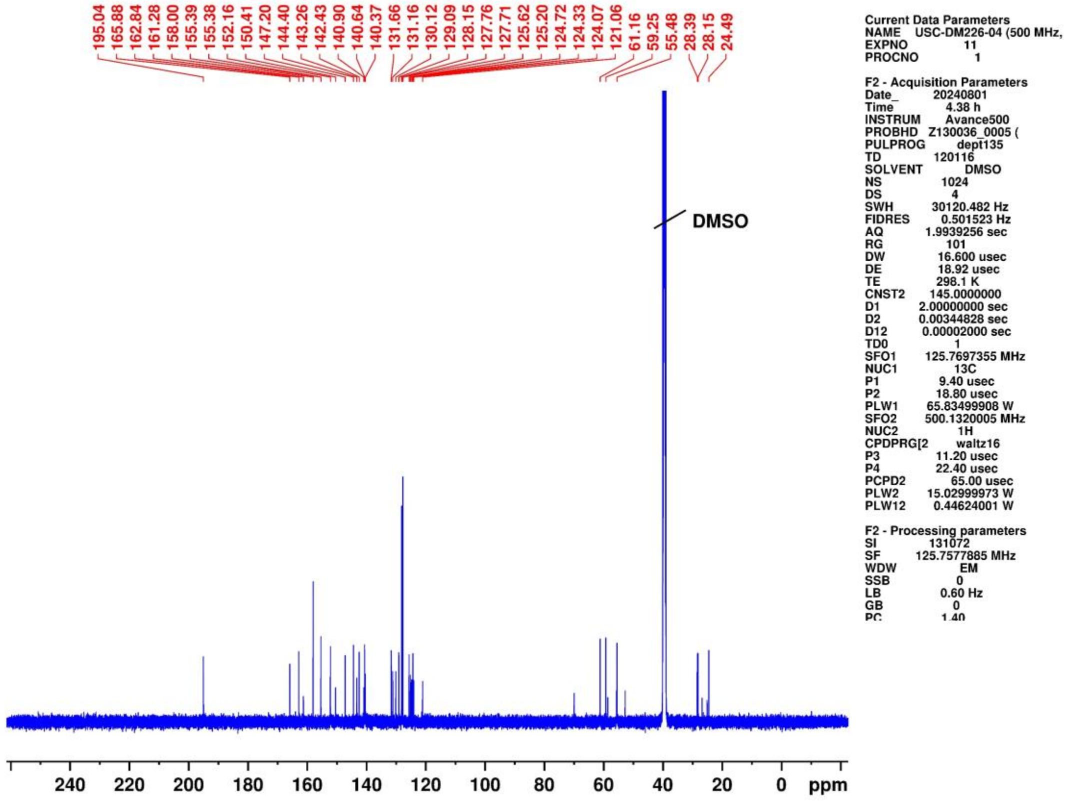

Figure S33: 125.76 MHz ^{13}C NMR spectrum of **18** in DMSO-d_6.

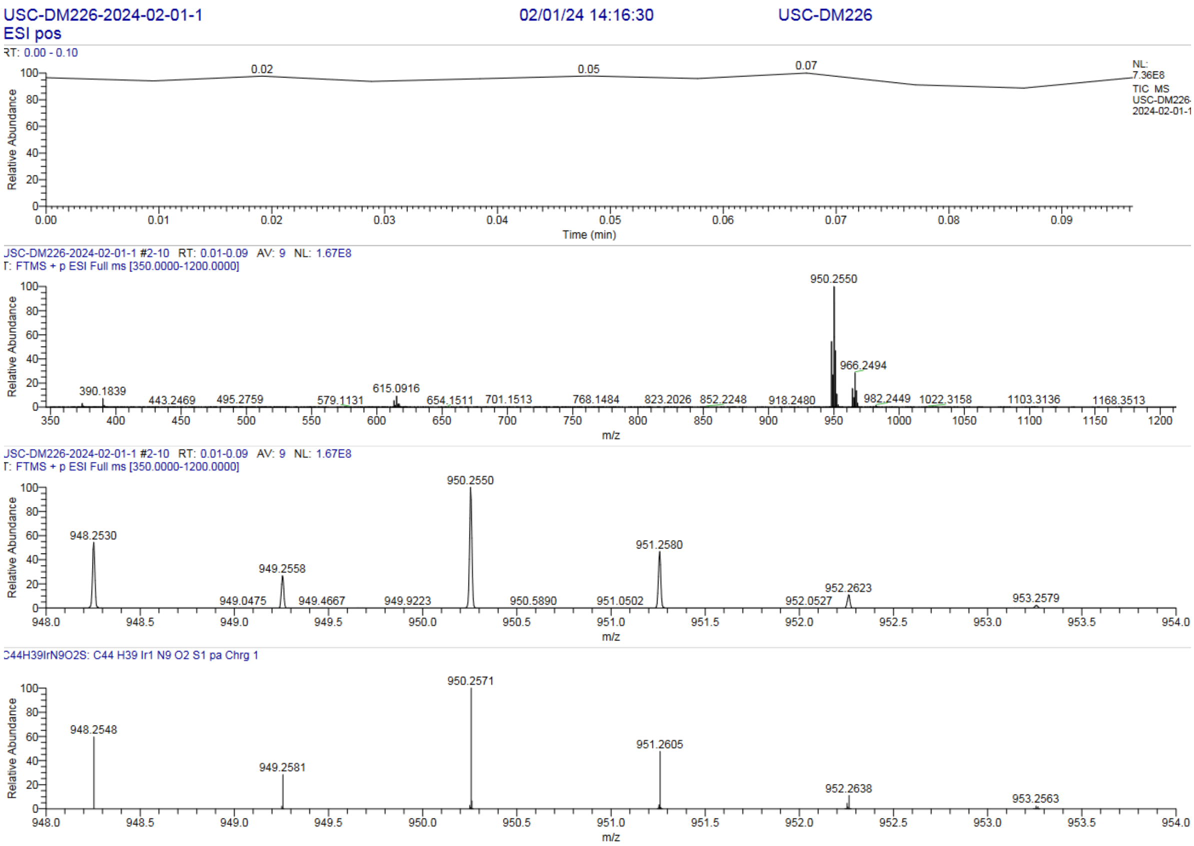

Figure S34: ESI positive mode mass spectrum of **18** in methanol.

Log*P* measurements

The distribution coefficient log*P* of compound **5** and complexes **11**–**18** was determined using the "shake-flask" method.[6],[7] In short, phosphate buffer (50 mM, pH 7.4) was saturated with *n*-octanol by shaking a 1:1 mixture of both solution on a laboratory shaker for 72 h, after which the phases were separated. Stock solutions of each complex were then prepared in dimethylsulfoxide at a concentration of 10 mM.

Compounds **15**–**18** had to be further diluted with phosphate buffer to give a 2 mL solution with a concentration of 100 μM. From this solution, 1000 μL were transferred into an Eppendorf tube and mixed with 1000 μL of *n*-octanol. After shaking for 15 min and centrifugation (5 min, 6000 rpm), the two phases were carefully separated, and the contact layer discarded. Then, the absorbance of each phase was recorded with an Agilent 8453 UV/Vis spectrometer.

Compounds **5** and complexes **11**–**14** had to be further diluted with *n*-octanol to give a 2 mL solution with a concentration of 100 μM. From this solution, 1000 μL were transferred into an Eppendorf tube and mixed with 1000 μL phosphate buffer. After shaking for 15 min and centrifugation (5 min, 6000 rpm), the phases were carefully separated, and the contact layer discarded. Then, the absorbance of each phase was recorded with an Agilent 8453 UV/Vis spectrometer.

HABA/Avidin Assay

Biotin alkyne **5** and triazolato complexes **11–18** were studied for their binding properties towards avidin using the HABA assay.[8] A mixture of avidin (6.8 μM, SIGMA-ALDRICH: **A9275**) and 4'-hydroxyazobenzene-2-carboxylic acid (HABA, 0.3 mM, SIGMA-ALDRICH: **H5126**) was dissolved in 50 mM sodium phosphate buffer (20 mL, pH 7.4) and allowed to equilibrate for 24 h at 4 °C. Stock solutions of each compound were prepared in dimethyl sulfoxide at a concentration of 1.1 mM. The binding was assessed by adding aliquots of the biotin compound stock solution (2 μL) to the prepared avidin/HABA mixture (1 mL) at room temperature at 5–10 min intervals until a stable absorbance value was observed at each addition of aliquot, and the absorbance then recorded with an Agilent 8453 UV/Vis spectrometer. The formation of the adduct was indicated by a decrease in the absorbance at 500 nm due to the displacement of HABA from the avidin. By plotting -ΔA500 nm *vs*. the molar concentration ratio *c*(compound):*c*(avidin), the binding stoichiometry of the compound to avidin was determined.

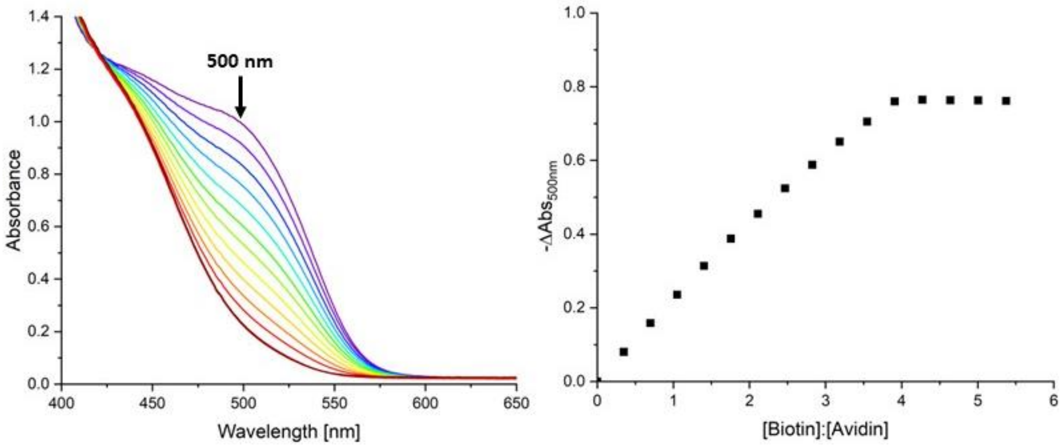

Figure S35: (left) Changes in the UV/Vis absorption spectrum upon titration of the avidin/HABA adduct with unmodified biotin; (right) -ΔAbs$_{500nm}$ *vs*. ratio of c(biotin):c(avidin).

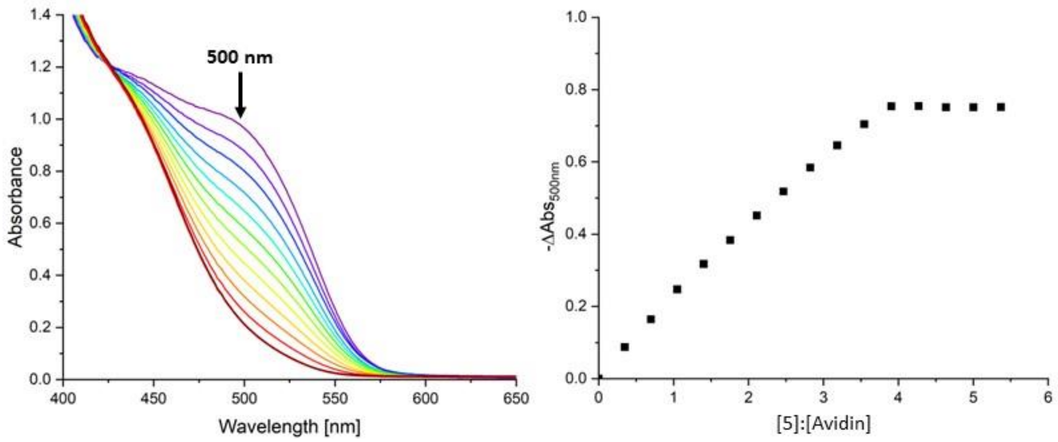

Figure S36: (left) Changes in the UV/Vis absorption spectrum upon titration of the avidin/HABA adduct with biotin-alkyne **5**; (right) -ΔAbs$_{500nm}$ *vs*. ratio of c(**5**):c(avidin).

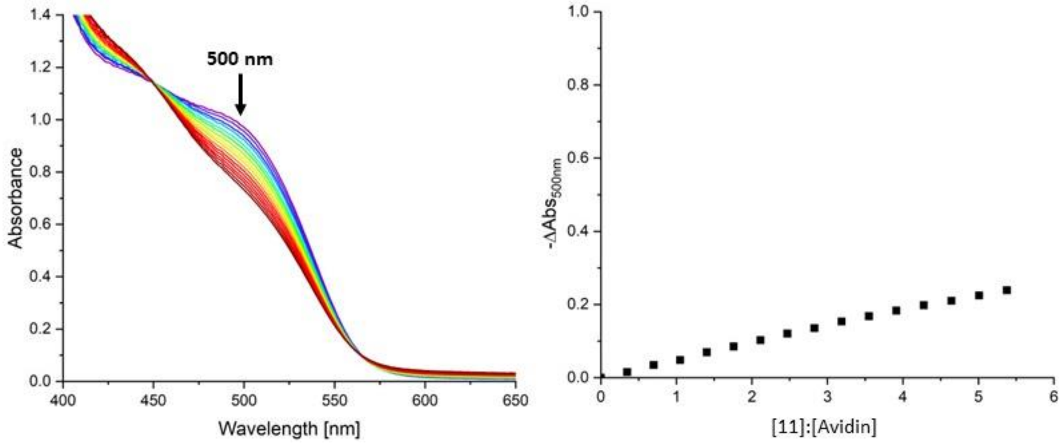

Figure S37: (left) Changes in the UV/Vis absorption spectrum upon titration of the avidin/HABA adduct with [Au(triazolato^{C6H5,COCH3})(PPh$_3$)] **11**; (right) -ΔAbs$_{500nm}$ *vs*. ratio of c(**11**):c(avidin).

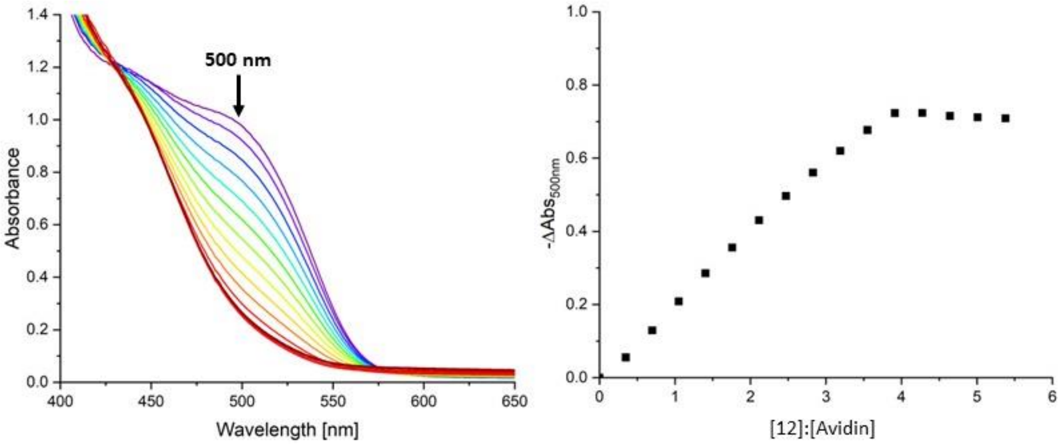

Figure S38: (left) Changes in the UV/Vis absorption spectrum upon titration of the avidin/HABA adduct with [Au(triazolato^{C6H5,biotin})(PPh_3)] **12**; (right) $-\Delta Abs_{500nm}$ *vs*. ratio of c(**12**):c(avidin).

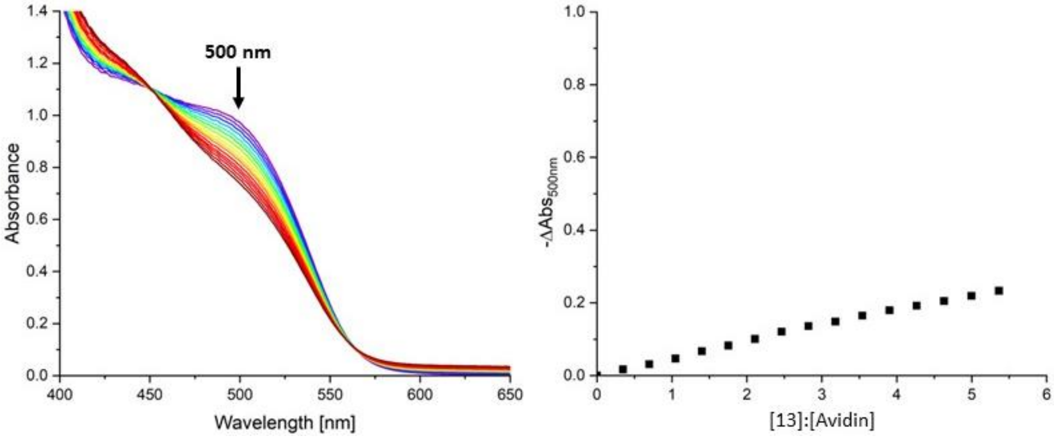

Figure S39: (left) Changes in the UV/Vis absorption spectrum upon titration of the avidin/HABA adduct with [Pt(triazolato^{COOCH3,COOCH3})(dpb)] **13**; (right) $-\Delta Abs_{500nm}$ *vs*. ratio of c(**13**):c(avidin).

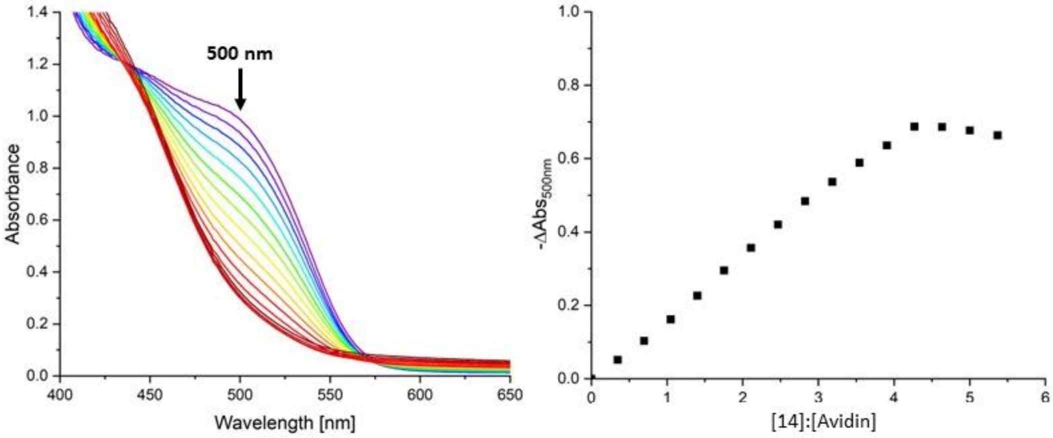

Figure S40: (left) Changes in the UV/Vis absorption spectrum upon titration of the avidin/HABA adduct with [Pt(triazolato^{C6H5,biotin})(dpb)] **14**; (right) $-\Delta Abs_{500nm}$ *vs*. ratio of c(**14**):c(avidin).

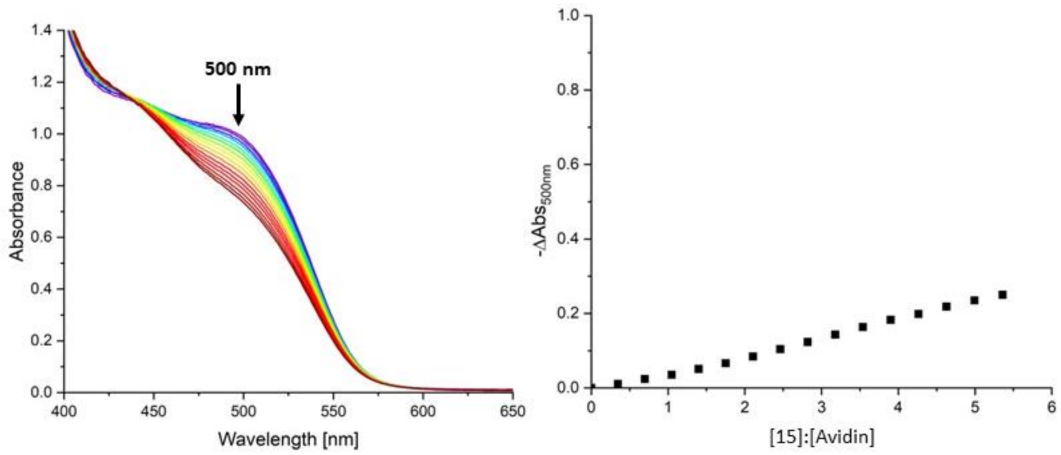

Figure S41: (left) Changes in the UV/Vis absorption spectrum upon titration of the avidin/HABA adduct with [Pt(triazolato^{COOCH3,COOCH3})(terpy)] **15**; (right) -ΔAbs$_{500nm}$ *vs*. ratio of c(**15**):c(avidin).

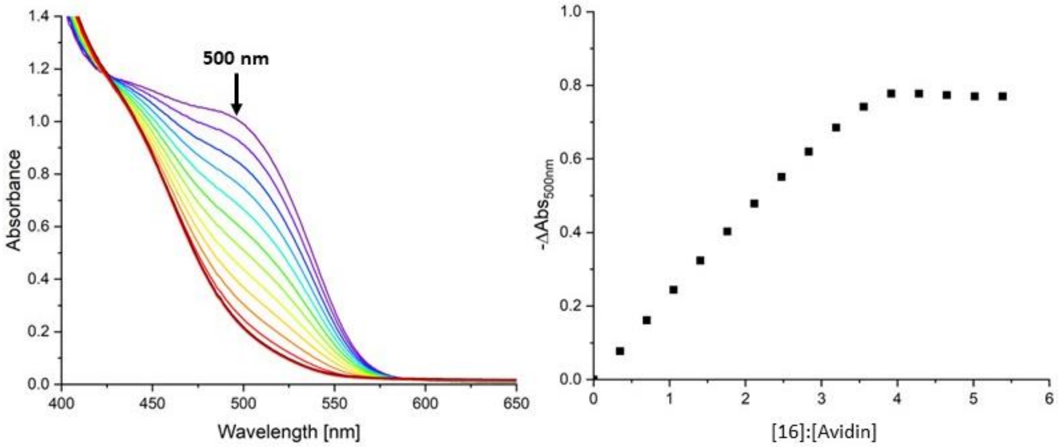

Figure S42: (left) Changes in the UV/Vis absorption spectrum upon titration of the avidin/HABA adduct with [Pt(triazolato^{C6H5,biotin})(terpy)] **16**; (right) -ΔAbs$_{500nm}$ *vs*. ratio of c(**16**):c(avidin).

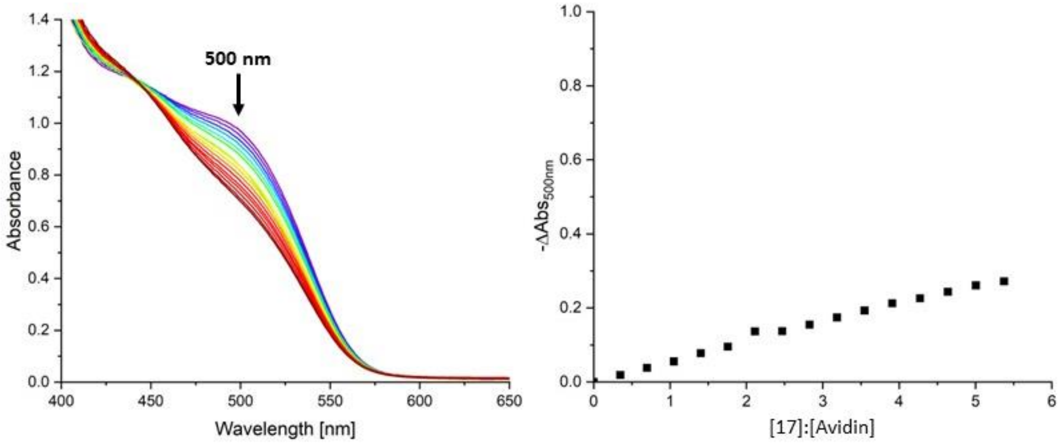

Figure S43: (left) Changes in the UV/Vis absorption spectrum upon titration of the avidin/HABA adduct with [Ir(triazolato^{COOCH3,COOCH3})(ppy)(terpy] **17**; (right) -ΔAbs$_{500nm}$ *vs*. ratio of c(**17**):c(avidin).

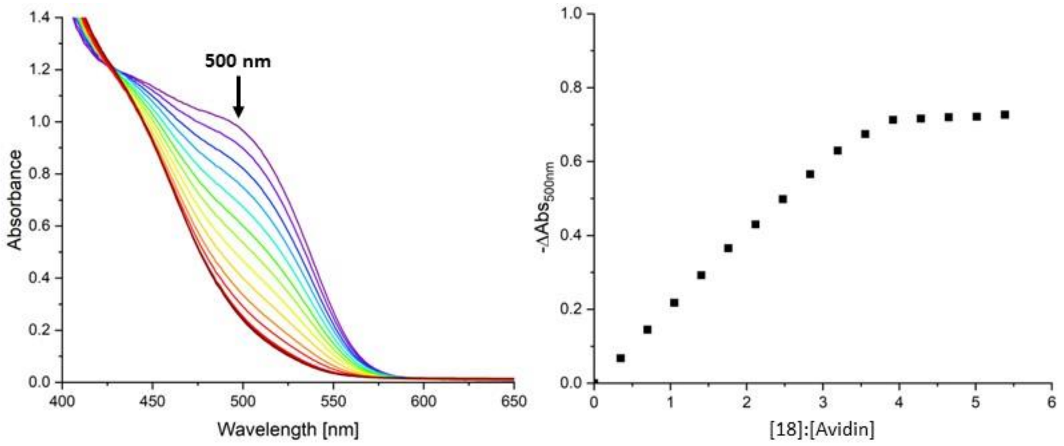

Figure S44: (left) Changes in the UV/Vis absorption spectrum upon titration of the avidin/HABA adduct with [Ir(triazolato^{C6H5,biotin})(ppy)(terpy] **18**; (right) $-\Delta Abs_{500nm}$ *vs*. ratio of c(**18**):c(avidin).

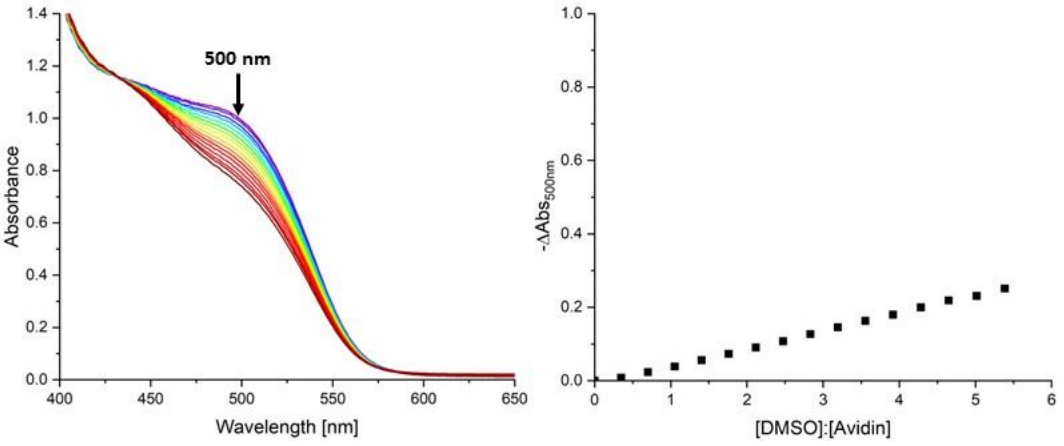

Figure S45: (left) Changes in the UV/Vis absorption spectrum upon titration of the avidin/HABA adduct with pure dimethylsulfoxide; (right) $-\Delta Abs_{500nm}$ *vs*. ratio of c(compound):c(avidin) as baseline.

Isothermal titration calorimetry

Streptavidin (20 µM in 10 mM HEPES plus 2% DMSO) was titrated stepwise with 2 uL injections of the compound (200 µM in 10 mM HEPES + 2% DMSO) at 25 °C, using a Malvern MicroCal PEAQ-ITC. Raw thermograms (top panels) and the binding isotherm from the integrated thermogram (bottom panels) were fit using the One Set of Sites model in the MicroCal PEAQ-ITC analysis software. Circles indicate the integrated heat, and the curve represents the best fit.

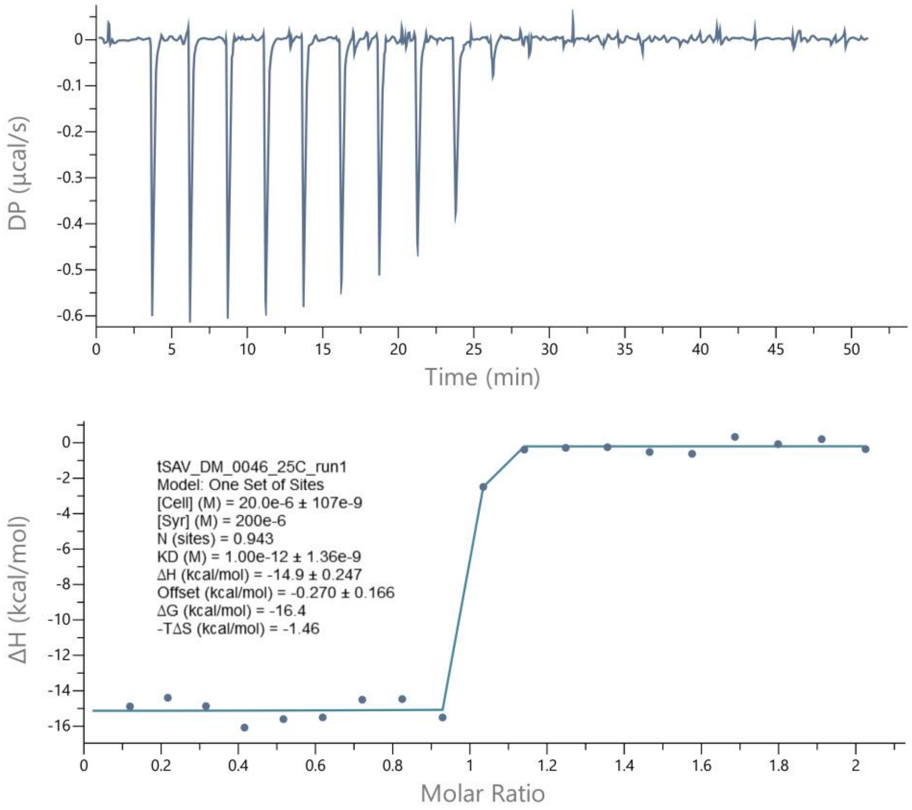

Figure S46: Isothermal titration calorimetry measurements of **5**.

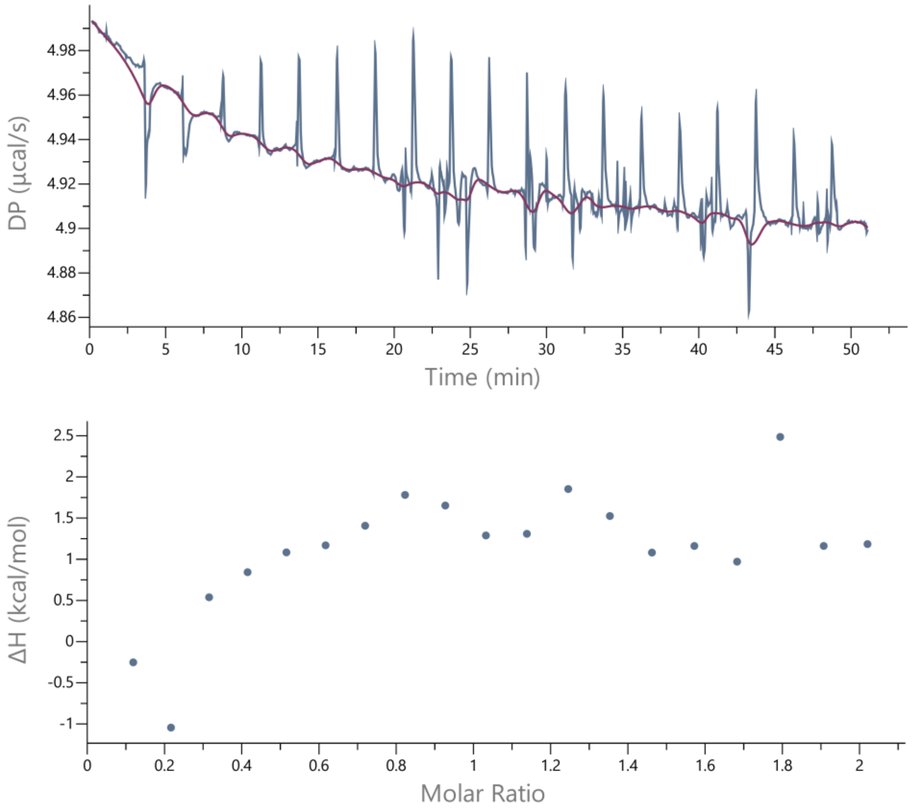

Figure S47: Isothermal titration calorimetry measurements of **15**.

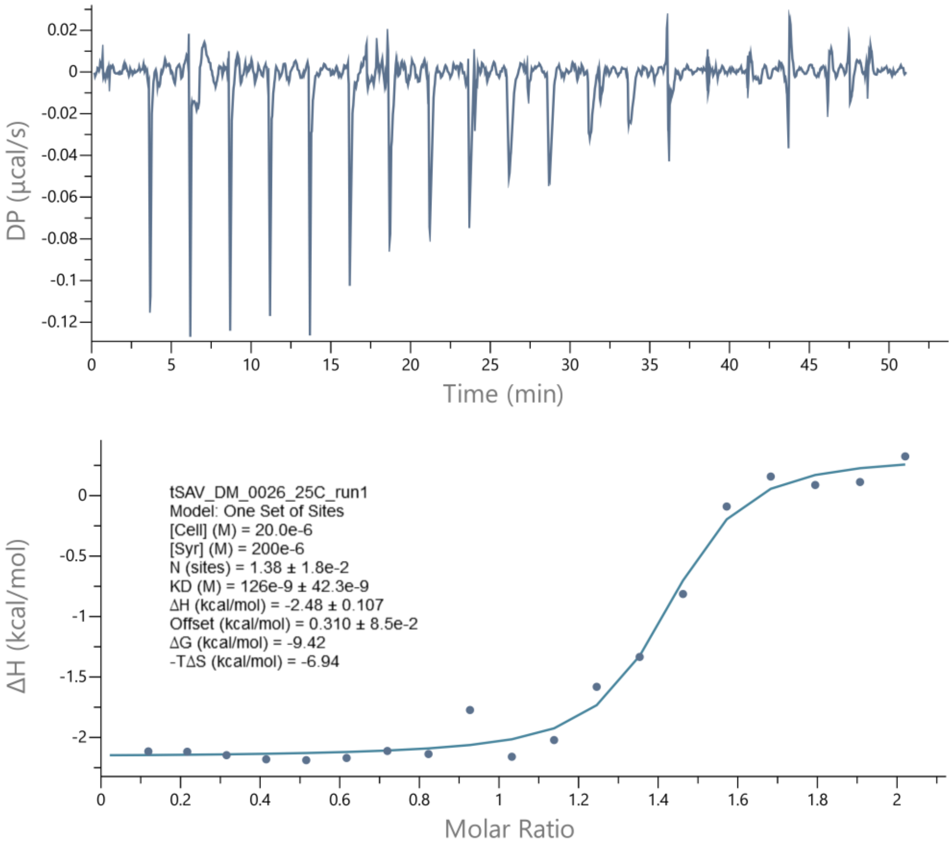

Figure S48: Isothermal titration calorimetry measurements of **16**.

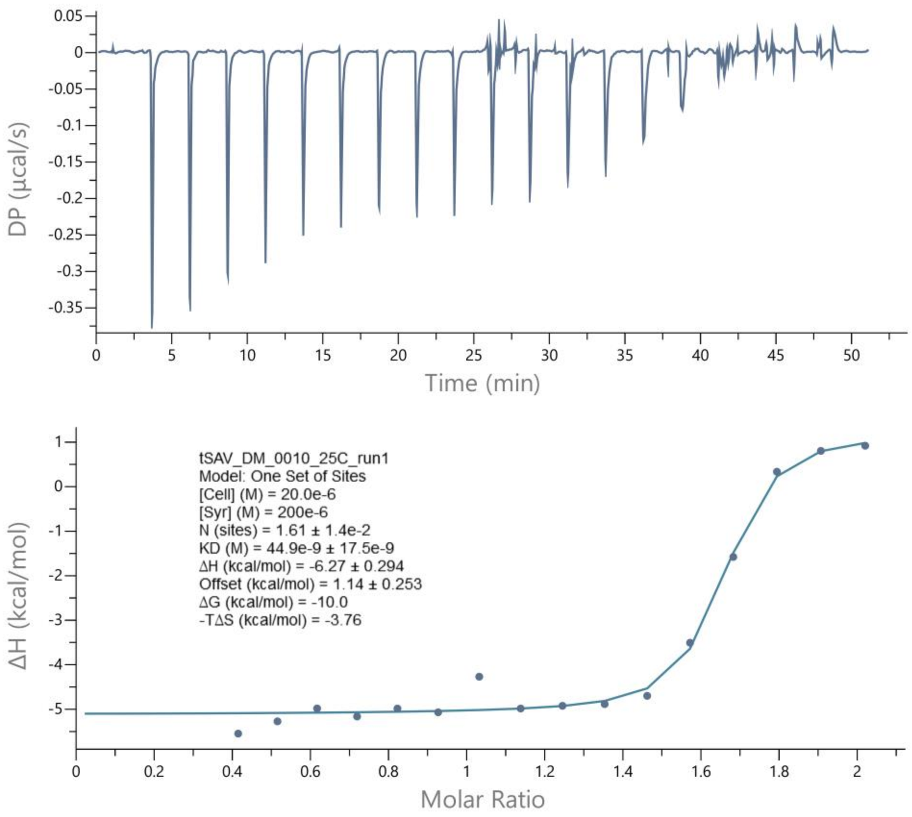

Figure S49: Isothermal titration calorimetry measurements of **18**.

Antibacterial activity assay

Rapid screen broth microdilution assay

5 × 125 mL Erlenmeyer flasks, each filled with 12.5 mL Mueller Hinton broth II (MHBII), were inoculated with *Klebsiella pneumoniae* ATCC 13883, *Acinetobacter baumanii* ATCC 17978, *Psuedomonas aeruginosa* ATCC BAA-47, *Staphylococcus aureus* ATCC 29213, and *Escherichia coli* K12 ATCC 10798. 1 × 125 mL Erlenmeyer flask with 12.5 mL BHI broth was inoculated with *Enterococcus faecium* ATCC 19434. The cultures were incubated at 37 °C with shaking at 180 rpm for 16 h. The suspensions were diluted 100-fold with the requisite broth (*E. faecium*: 4-fold) and the resultant suspensions incubated for a further 3 h at 37 °C with shaking. The cultures had an OD of 0.8–1.2 after 3 h. Stock solutions of the title compounds were prepared in the requisite broth from 100 mM stocks in DMSO with the exceptions of carbenicillin, which was from a 100 mM stock in water and ciprofloxacin from a 10 mM stock in DMSO. 6 × 96 well plates were primed with a concentration gradient of the analytes from 200 to 3.13 μM, carbenicillin from 200 to 3.13 μM and ciprofloxacin from 10 to 0.16 μM. The cells were diluted 100-fold to an OD_{600} of 0.01. All plates were then inoculated with 50 μL per well bacterial culture in the requisite broth to 100 μL per well. Plates were then incubated at 37 °C for 24 h before reading OD_{600} on a BMG Clariostar platereader.

MIC broth microdilution assay

3 × 125 mL Erlenmeyer flasks, each filled with 12.5 mL MHBII, were inoculated with three different colonies of *S. aureus* NTCC 12973. 3 × 125 mL Erlenmeyer flasks, each filled with 12.5 mL BHI broth, were inoculated with three different colonies of *E. faecium ATCC 19434*. The cultures were incubated at 37 °C with shaking at 180 rpm for 16 h. *S. aureus* cultures had an OD_{600} of ~9.0. The resultant *E. faecium* cultures had an OD_{600} of ~2.5. The suspensions of *S. aureus* cultures were diluted 100-fold with MHBII while the *E. faecium* cultures were diluted 4-fold. The resultant suspensions were incubated for a further 3 h at 37 °C with shaking. The cultures had an OD of 0.8–1.2 after 3 h. Stock solutions of the title compounds were prepared in the requisite broth from 100 mM stocks in DMSO with the exception of carbenicillin, which was from a 100 mM stock in water and vancomycin from a 10 mM stock in DMSO. 15 × 96 well plates were primed with concentration gradients of the analytes and carbenicillin as shown in Figure S60 for *S. aureus*. 15 × 96 well plates were primed with the concentration gradients of the analytes and vancomycin as shown in Figure S61 for *E. faecium*. The cells were diluted 100-fold to an OD_{600} of 0.01. All plates were then inoculated with 50

μL per well of the relevant bacterial culture in the requisite broth to 100 μL per well. Plates were then incubated at 37 °C for 24 h before reading OD_{600} on a BMG Clariostar platereader. NB. The *E. faecium* colonies showed significant heterogeneity in response to **5** and **11–18** and the data is plotted separately in Figure S61.

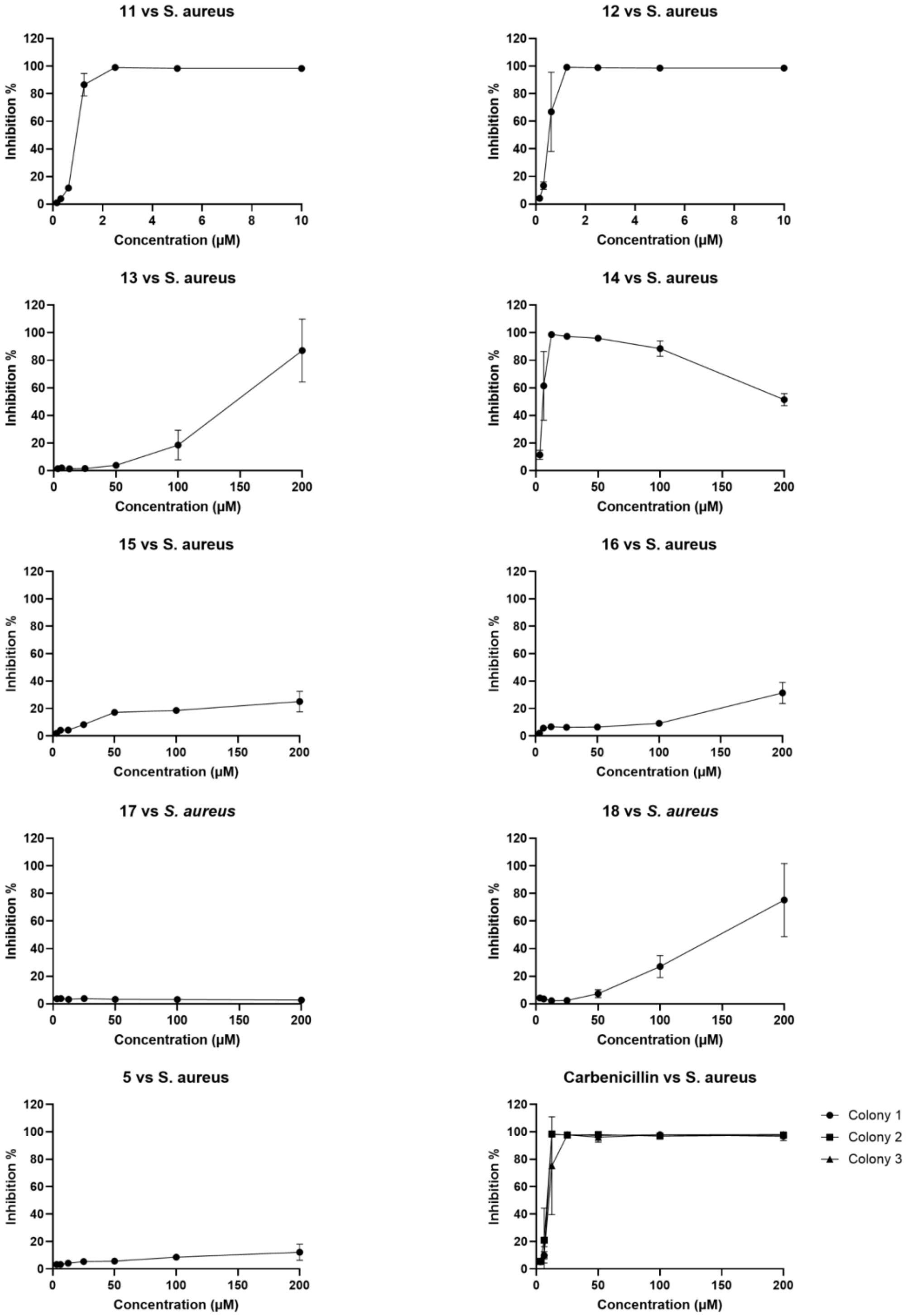

Figure S50: Dose response profiles of the title compounds against *S. aureus*.

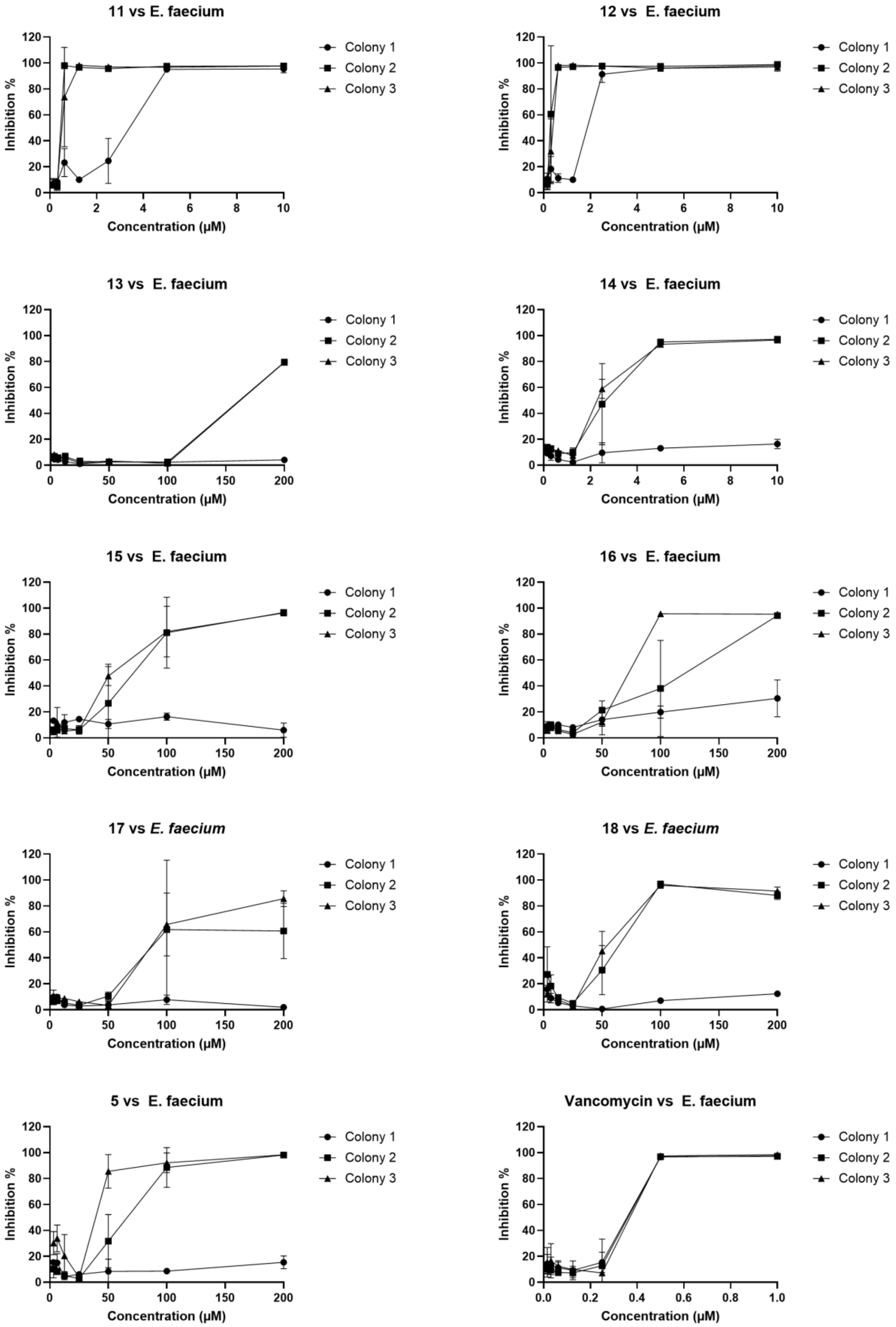

Figure S51: Dose response profiles of the title compounds against *E. faecium*.

Cell viability studies

Compound cytotoxicity was tested simultaneously on five representative cell lines (HaCaT, HeLa, HCT116, hTERT RPE-1, and BEAS-2B). All cells were cultured in DMEM Glutamax without pyruvate with 10% fetal bovine serum and 1% penicillin/streptomycin (Gibco). Cells were incubated with the compound for 24 or 48 h at 37 °C and 5% CO_2 (*v*/*v*). The tested concentration range for each compound was made of eight points, following a 3-fold dilution starting from 10 μM. The required amount of compound was distributed in each well of the 384-well assay plate (Bio-one, Greiner CELLSTAR® microplates) using the Echo 550 Acoustic Liquid Handler and wells were back-filled with DMSO when required to keep a final DMSO concentration of 0.5 %. Next, 50 μL of cell solution was added to the compound-containing wells using the Integra® Viafill Bulk dispenser. Cell solutions were prepared to get 5000 (24 h)/3500 (48 h) cells per well for HaCaT, 5000 (24hr)/2500 (48hr) cells per well for HeLa, 10000 (24 h)/5000 (48 h) cells per well for HCT116, 5000 (24 h)/1500 (48 h) cells per well for hTERT RPE-1, and 5000 (24 h)/2500 (48 h) cells per well for BEAS-2B. After incubation, cells were fixed with 4% PFA (Merck, F8775), permeabilized with 0.2% Triton X-100 (Merck, T8787) and stained to visualize their nuclei using 2 μg/mL DAPI (Merck, 10236276001). Viable cells were counted using 377 nm excitation and 447 nm emission filters on the Celigo™ Image Cytometer. The number of nuclei per well was determined using the Direct Cell Count analysis from the CeligoPro Sofware (5.5.1.0). All conditions were performed in triplicate. Staurosporine (Cambridge Bioscience, s7600) was used as a positive control and DMSO only as a negative control. The percentage of cell viability was normalized to the DMSO-only control and IC_{50} values were extracted using an in-house Python script based on a 4-parameter logistic curve with the `scipy.optimize.curve_fit` function.

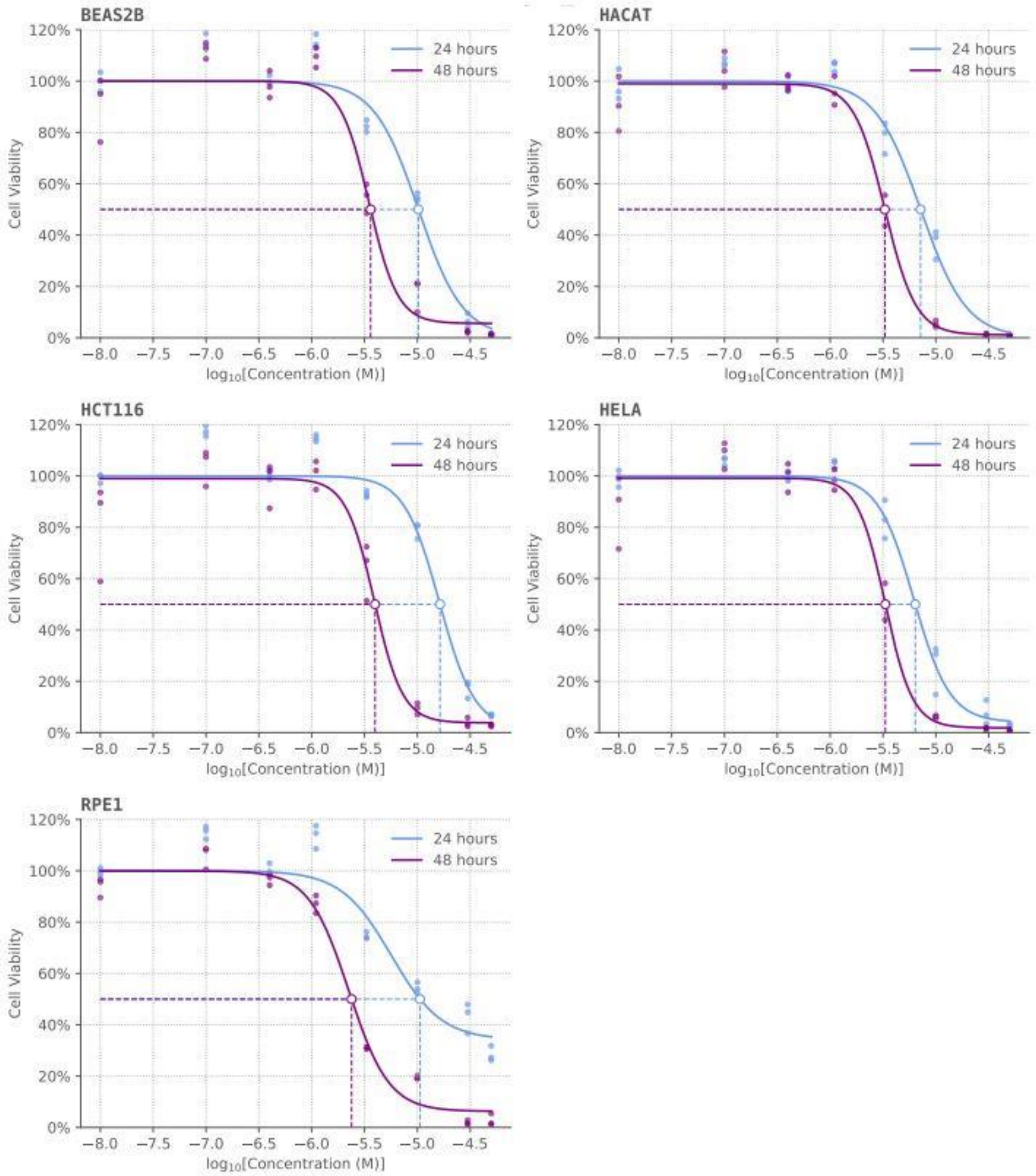

Figure S52: Dose-response curves of **5** for BEAS2B, HACAT, HCT116, HeLa and RPE1 cell lines.

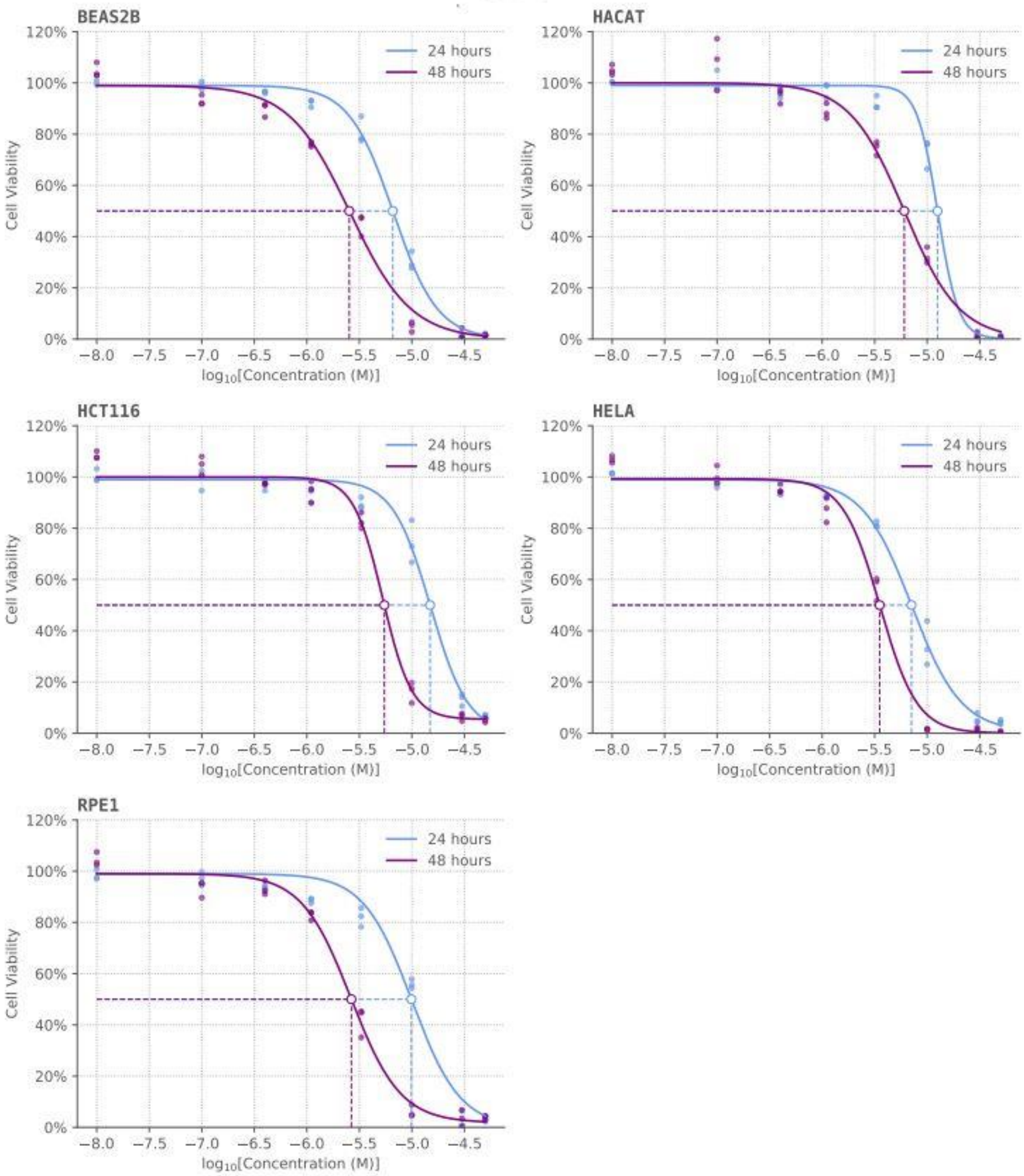

Figure S53: Dose-response curves of **11** for BEAS2B, HACAT, HCT116, HeLa and RPE1 cell lines.

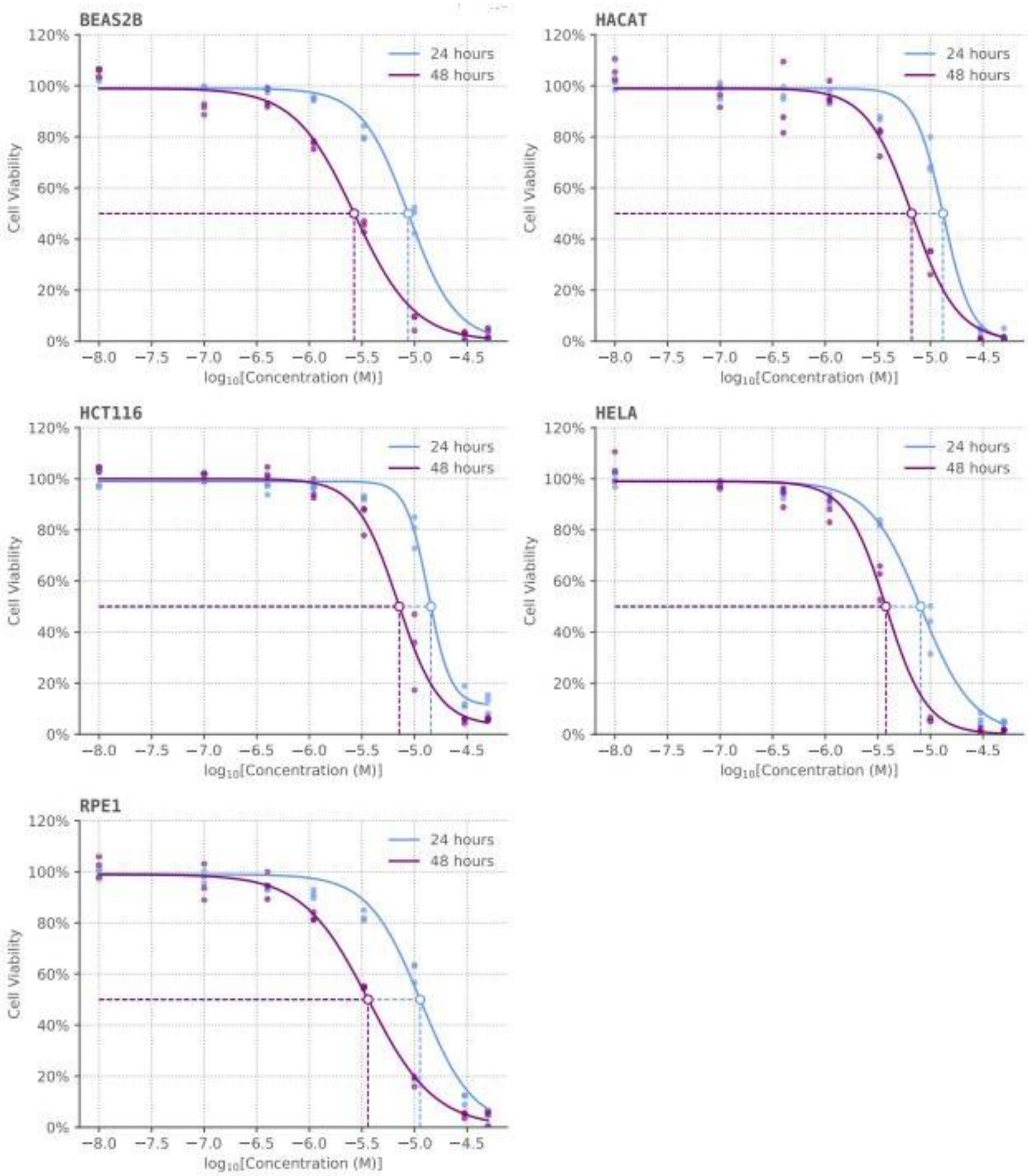

Figure S54: Dose-response curves of **12** for BEAS2B, HACAT, HCT116, HeLa and RPE1 cell lines.

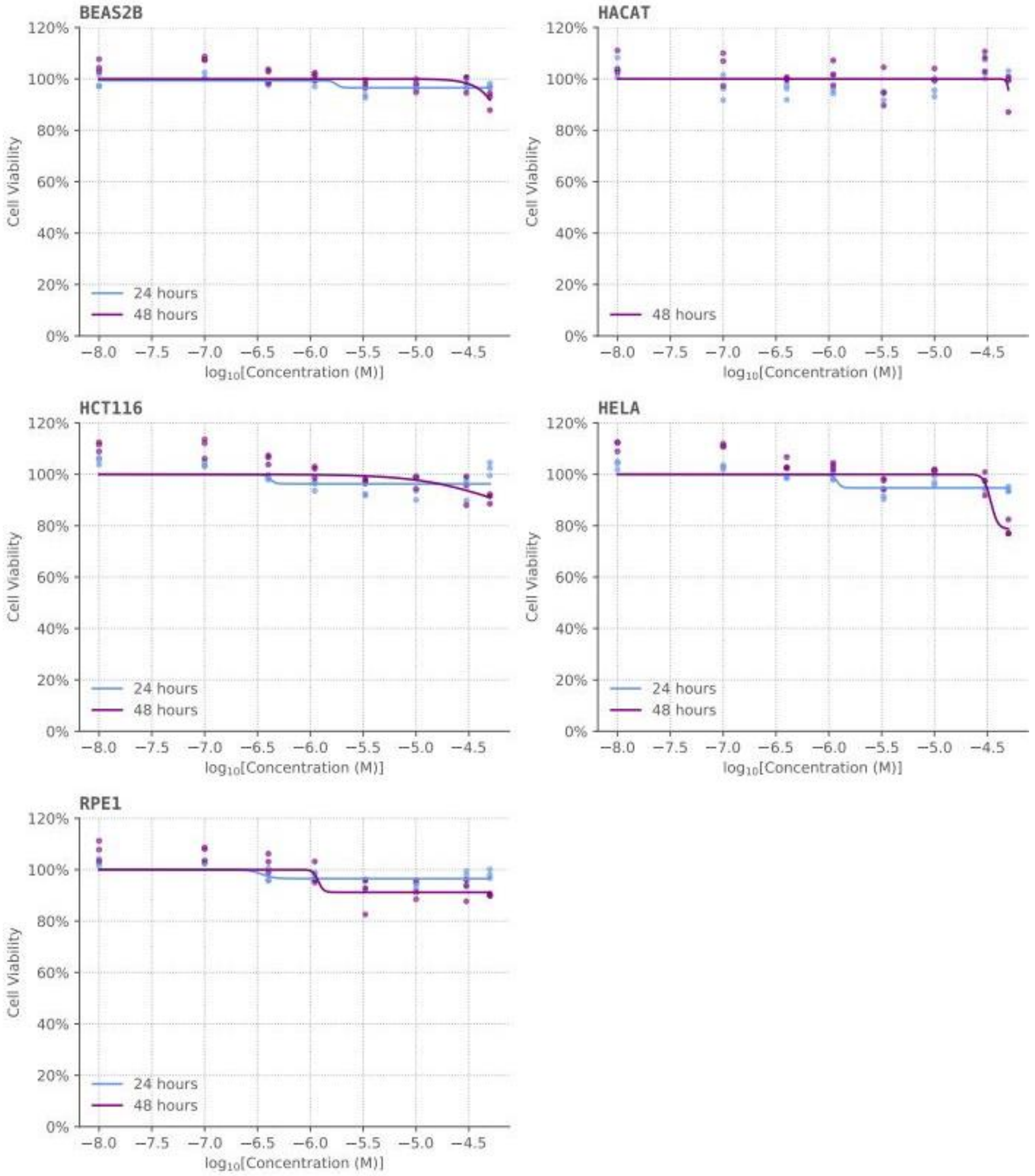

Figure S55: Dose-response curves of **13** for BEAS2B, HACAT, HCT116, HeLa and RPE1 cell lines.

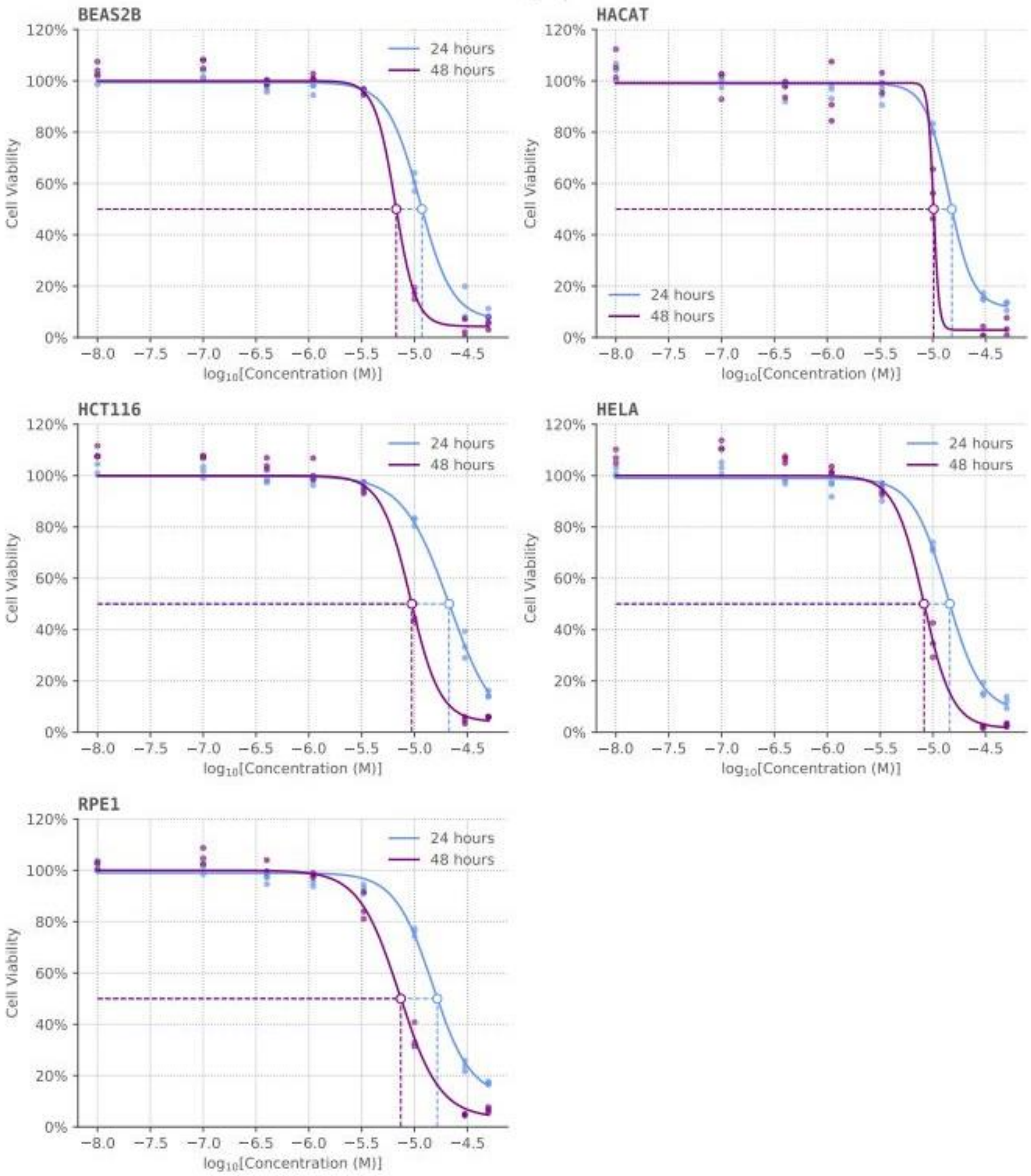

Figure S56: Dose-response curves of **14** for BEAS2B, HACAT, HCT116, HeLa and RPE1 cell lines.

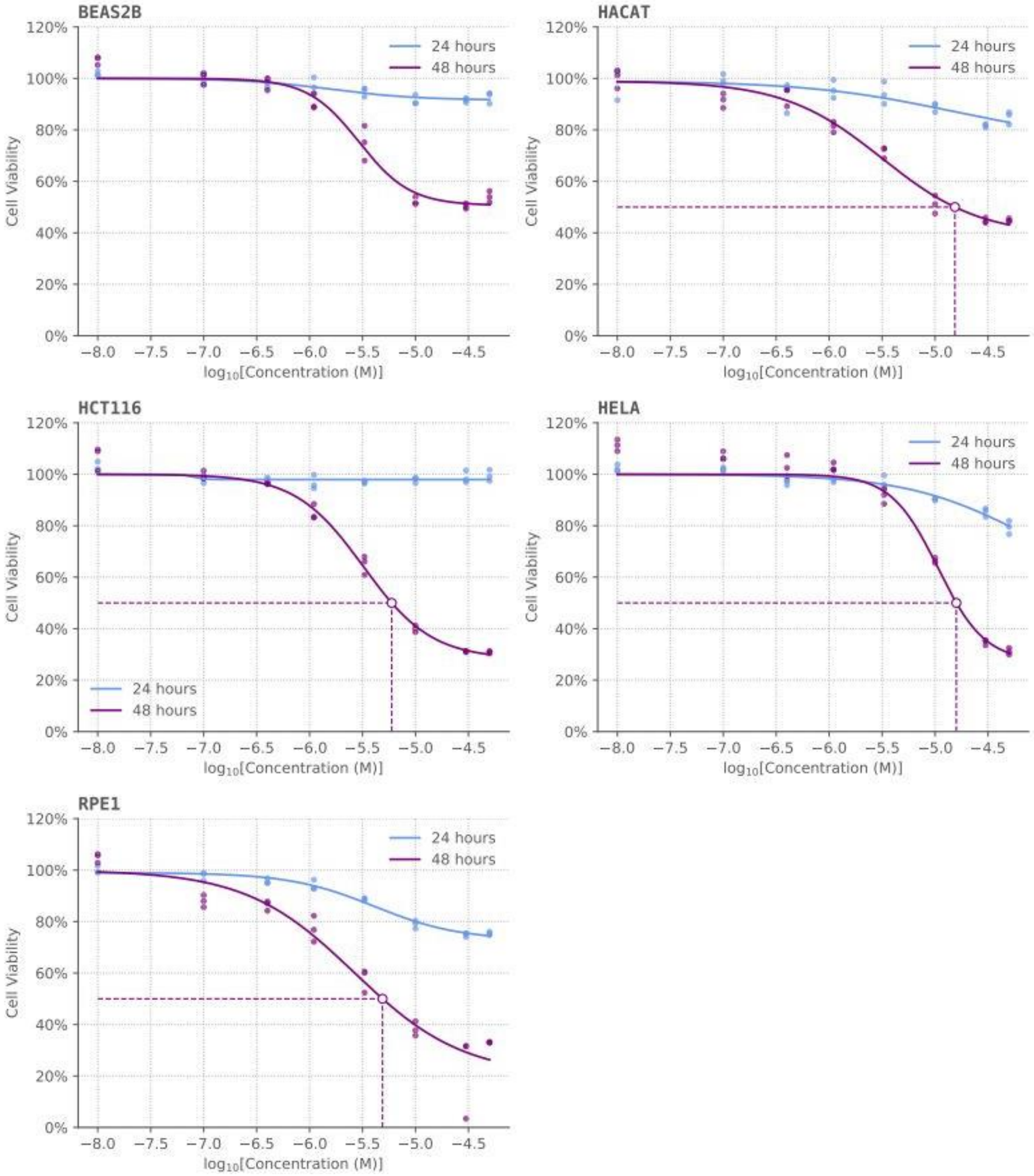

Figure S57: Dose-response curves of **15** for BEAS2B, HACAT, HCT116, HeLa and RPE1 cell lines.

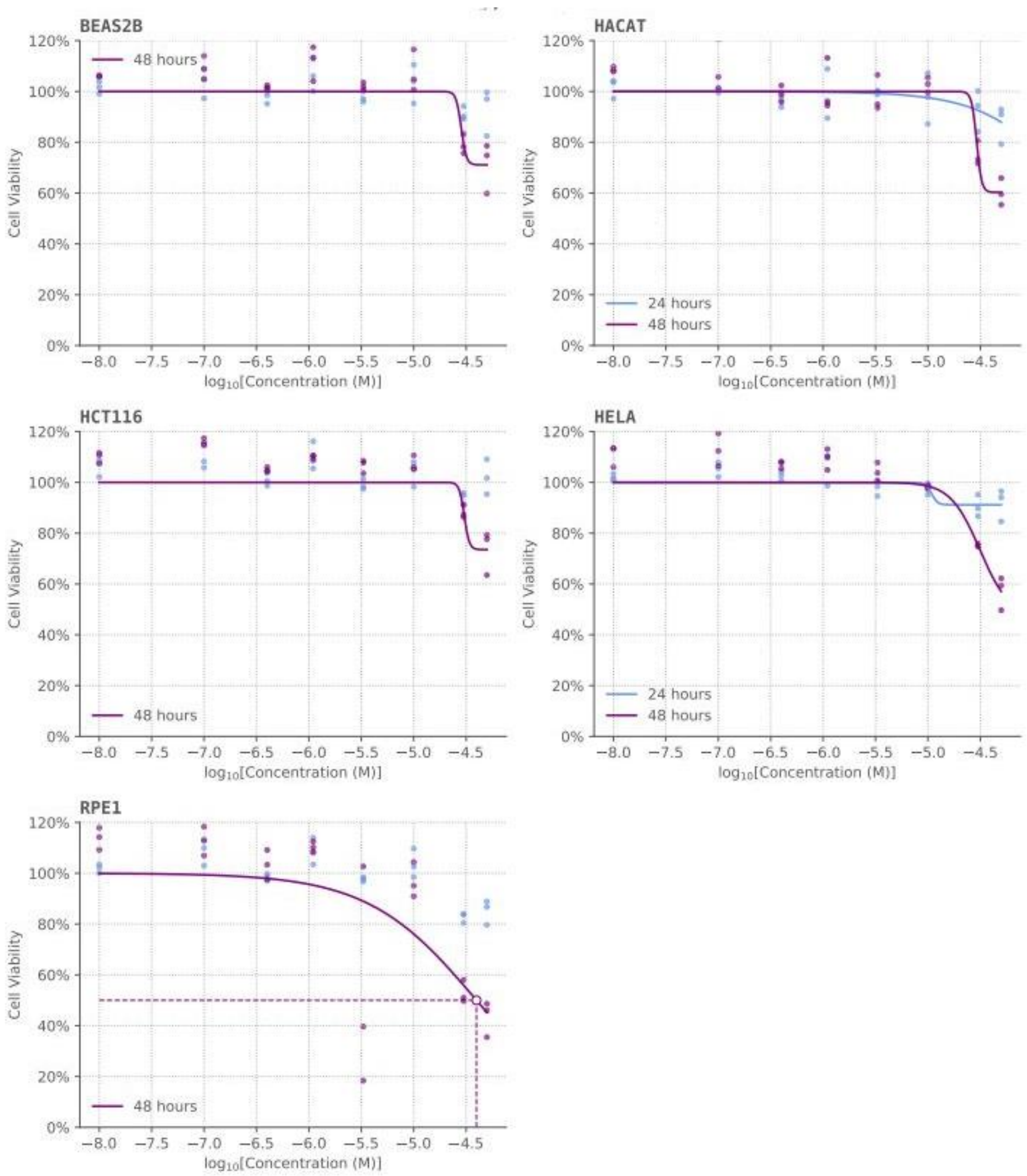

Figure S58: Dose-response curves of **16** for BEAS2B, HACAT, HCT116, HeLa and RPE1 cell lines.

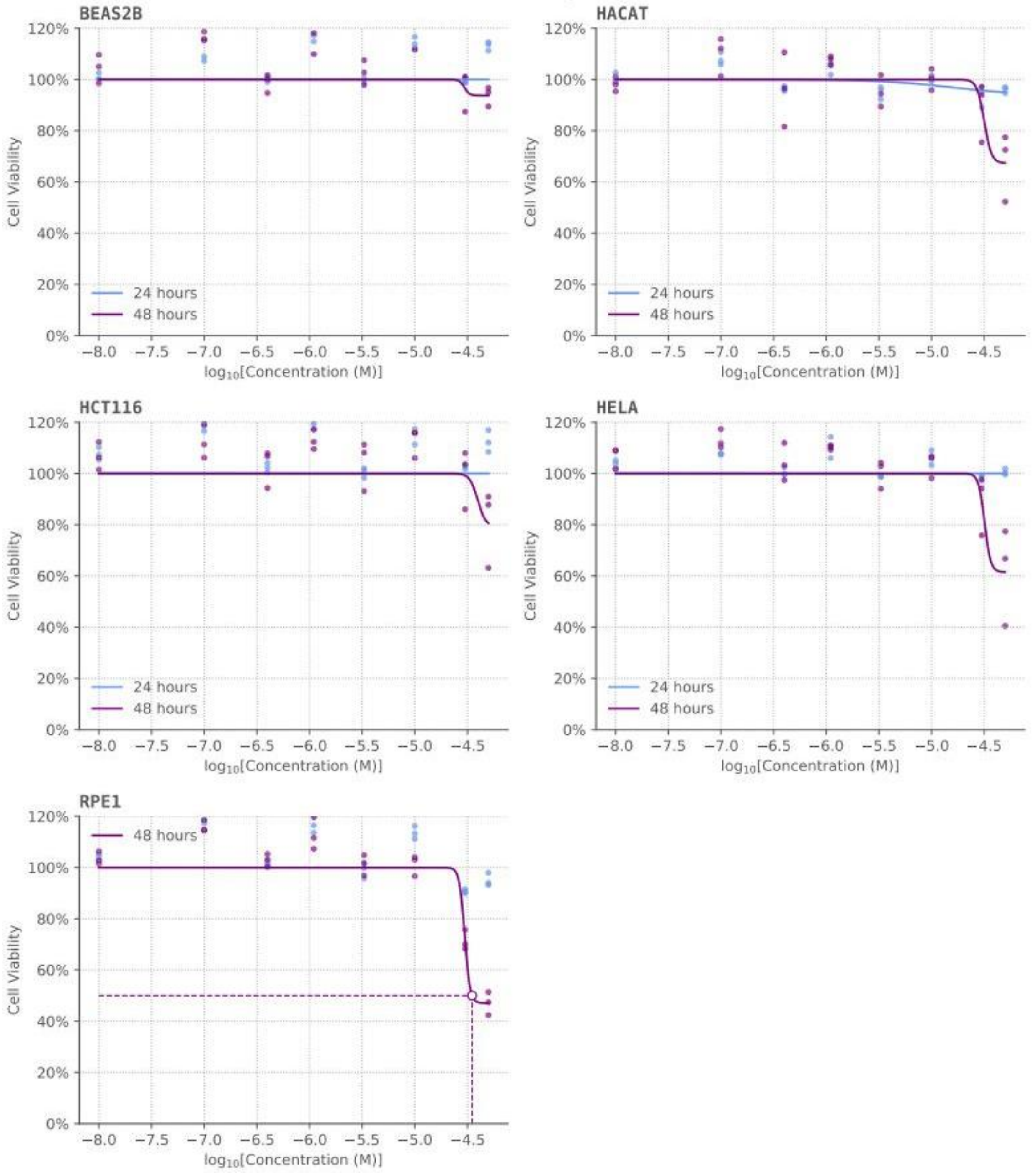

Figure S59: Dose-response curves of **17** for BEAS2B, HACAT, HCT116, HeLa and RPE1 cell lines.

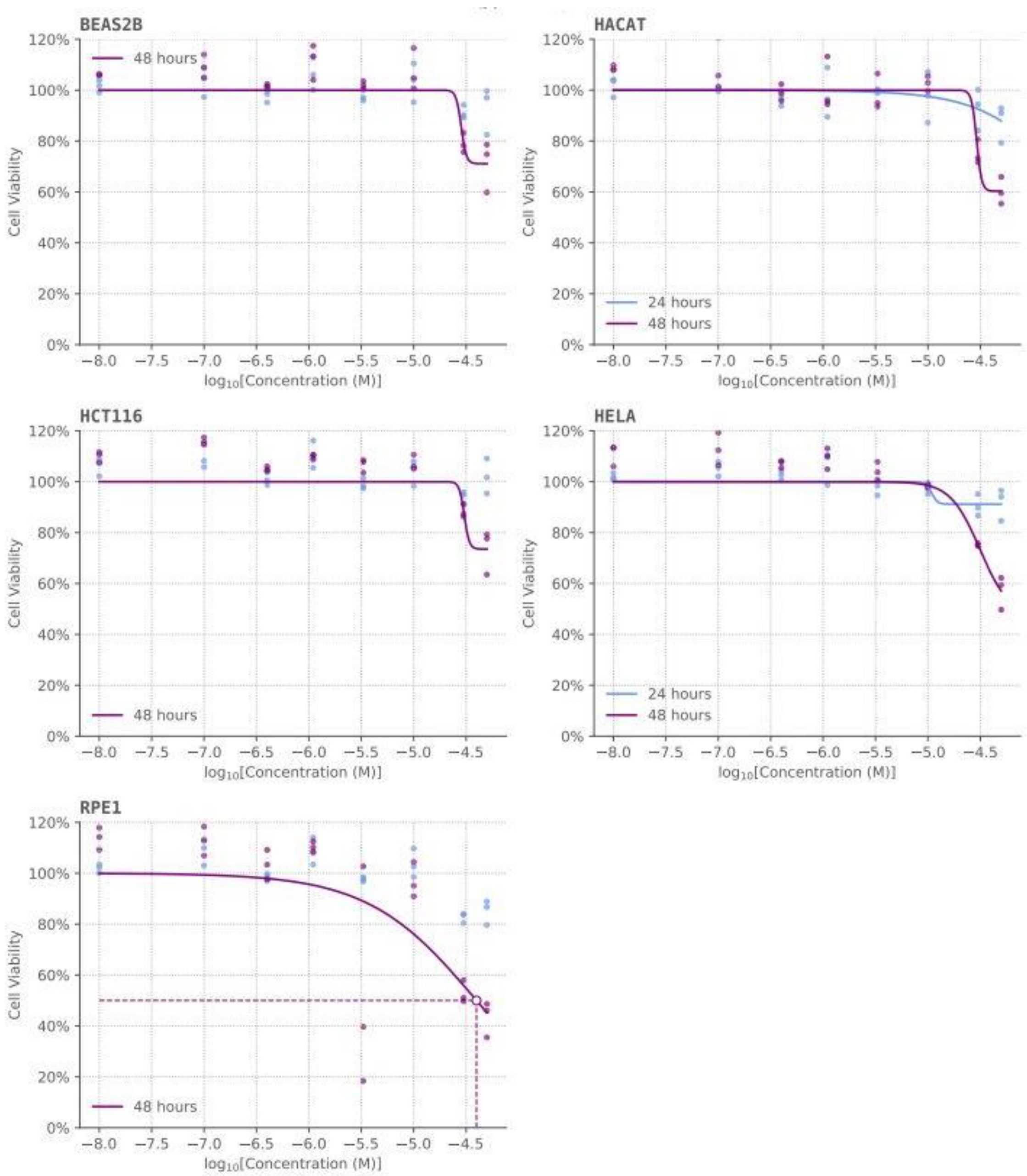

Figure S60: Dose-response curves of **18** for BEAS2B, HACAT, HCT116, HeLa and RPE1 cell lines.

References

1 Fulmer GR, Miller AJM, Sherden NH, Gottlieb HE, Nudelman A, Stoltz BM, Bercaw JE, Goldberg KI (2010) Nmr chemical shifts of trace impurities: Common laboratory solvents, organics, and gases in deuterated solvents relevant to the organometallic chemist. Organometallics 29:2176-2179. https://dx.doi.org/10.1021/om100106e

2 Del Castillo TJ, Sarkar S, Abboud KA, Veige AS (2011) 1,3-dipolar cycloaddition between a metal-azide (ph_3paun_3) and a metal acetylide ($ph_3pauccph$): An inorganic version of a click reaction. Dalton Trans 40:8140-8144. https://dx.doi.org/10.1039/c1dt10787a

3 Moreth D, Hörner G, Müller VVL, Geyer L, Schatzschneider U (2023) Isostructural series of ni(ii), pd(ii), pt(ii), and au(iii) azido complexes with a n^c^n pincer ligand to elucidate trends in the iclick reaction kinetics and structural parameters of the triazolato products. Inorg Chem 62:16000-16012. https://dx.doi.org/10.1021/acs.inorgchem.3c02122

4 Müller VVL, Simpson PV, Peng K, Basu U, Moreth D, Nagel C, Türck S, Oehninger L, Ott I, Schatzschneider U (2023) Taming the biological activity of pd(ii) and pt(ii) complexes with triazolato “protective” groups: ^{1}h, ^{77}se nuclear magnetic resonance and x-ray crystallographic model studies with selenocysteine to elucidate differential thioredoxin reductase inhibition. Inorg Chem 62:16203-16214. https://dx.doi.org/10.1021/acs.inorgchem.3c02701

5 Müller VVL, Moreth D, Kowalski K, Kowalczyk A, Gapińska M, Kutta RJ, Nuernberger P, Schatzschneider U (2024) Tuning the intracellular distribution of [3+2+1] iridium(iii) complexes in bacterial and mammalian cells by iclick reaction with biomolecular carriers functionalized with alkynone groups Chem Eur J:in print.

6 Leo A, Hansch C, Elkins D (1971) Partition coefficients and their uses. Chem Rev 71:525-616.

7 Simpson PV, Schmidt C, Ott I, Bruhn H, Schatzschneider U (2013) Synthesis, cellular uptake and biological activity against pathogenic microorganisms and cancer cells of rhodium and iridium n-heterocyclic carbene complexes bearing charged substituents. Eur J Inorg Chem 2013:5547-5554. https://dx.doi.org/https://doi.org/10.1002/ejic.201300820

8 Lo KK-W, Chan JS-W, Lui L-H, Chung C-K (2004) Novel luminescent cyclometalated iridium(iii) diimine complexes that contain a biotin moiety. Organometallics 23:3108-3116. https://dx.doi.org/10.1021/om0499355
